# A Novel Bioactive Component, Efoba, From Aged Garlic Targets CTSC to Suppress NET‐Driven Neuroinflammation and Ameliorate Secondary Brain Injury After Intracerebral Hemorrhage

**DOI:** 10.1002/advs.78538

**Published:** 2026-10-29

**Authors:** Junjun Meng, Jianhua Wang, Tao Zhang, Xiaofan Fan, Jinxiu Guo, Shiyuan Zhao, Wenxue Sun, Pei Jiang

**Affiliations:** ^1^ Translational Pharmaceutical Laboratory Jining NO.1 People's Hospital Shandong First Medical University Jining China

**Keywords:** aged garlic, CTSC, Efoba, ICH, NETs

## Abstract

Intracerebral hemorrhage (ICH) is a devastating neurological disorder with high mortality and disability, where neuroinflammation‐driven secondary brain injury is a key pathological hallmark. Neutrophil extracellular traps (NETs) serve as critical pathogenic mediators that amplify neuroinflammation, disrupt blood‐brain barrier integrity, and exacerbate brain damage after ICH. This study identifies Efoba, a novel bioactive monomer from aged garlic, and establishes a scalable total‑synthesis route for hundred‑gram‑scale high‑purity production, overcoming low‑yield and supply‑limiting bottlenecks for natural product research. In vivo and in vitro experiments show Efoba markedly ameliorates ICH‑induced neurological impairments and inhibits NET formation. Integrative analyses (Lip‐MS, virtual screening, proteomics, transcriptomics) identified cathepsin C (CTSC) as the direct binding target of Efoba. Efoba stabilizes microglial lysosomal membranes, inhibits CTSC release, blocks NET generation, and exerts neuroprotection against NETs‐driven neuroinflammation. CTSC knockout and CTSC overexpression assays further confirm the indispensable role of CTSC in mediating Efoba's neuroprotective and anti‑inflammatory activities. Collectively, this work demonstrates for the first time that Efoba targets CTSC to suppress NET‐mediated neuroinflammation, highlighting its therapeutic potential and the translational value of natural active components.

## Graphical abstract

Efoba, a novel bioactive component derived from aged garlic, confers neuroprotection against intracerebral hemorrhage. It acts on microglia to suppress CTSC release, inhibits NET‐mediated neural injury, and blocks pathological microglia‐neutrophil crosstalk. This study provides a highly promising lead compound for hemorrhagic‐stroke therapy.

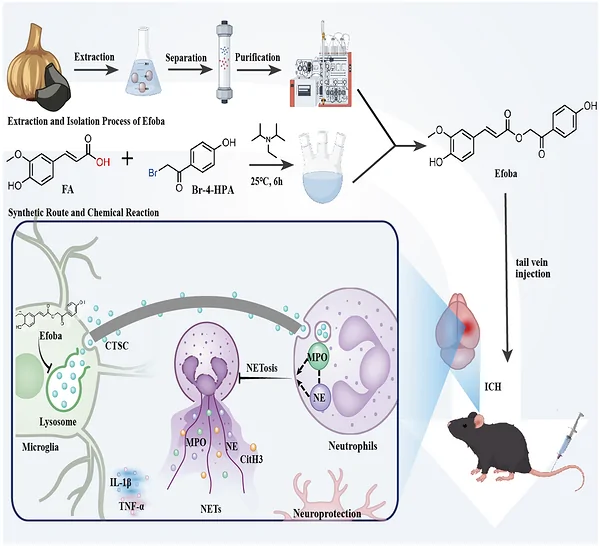

## 1 Introduction

Garlic is a traditional medicinal and edible plant. Mounting preclinical evidence confirms that garlic extracts exert anti‐inflammatory and neuroprotective effects in ICH, ischemic stroke, and Alzheimer's disease [1, 2, 3, 4, 5]. Nevertheless, its intense pungency and gastrointestinal irritation restrict clinical translational application. Aged garlic is produced by fermenting fresh garlic under conditions of 60°C–80°C and 70%–90% relative humidity for 60–90 consecutive days. This fermentation process removes irritant components and accumulates abundant bioactive small molecules, conferring stronger pharmacological activity compared with raw garlic [6, 7]. As a fermented derivative of raw garlic, aged garlic possesses improved pharmacological activity. Accumulating evidence has shown that aged garlic extract exerts neuroprotective effects in neurological disorders including stroke, largely through the modulation of oxidative stress, inflammation, and immune responses [8, 9]. Although the neuroprotective effects of aged garlic have been preliminarily confirmed, research on the specific components responsible for its neuroprotective activity remains insufficient. In particular, the key active constituents underlying its neuroprotective effects and their precise molecular mechanisms remain largely undefined, which severely hinders the development of aged garlic‐based innovative therapeutics.

Intracerebral hemorrhage (ICH) is clinically characterized by high mortality and high disability rates [10]. Its complex pathological process involves immune activation, oxidative stress, and inflammatory cascades triggered by hematoma formation, which eventually induce neuronal dysfunction and cell death [11]. Disturbed neuronal energy metabolism further aggravates tissue injury and necrosis and accelerates disease progression [12]. However, current therapeutic approaches are associated with a high risk of complications and limited efficacy in neurological functional recovery, and there remains a paucity of safe, low‐toxicity interventions that precisely target immune‐inflammatory pathways [13, 14]. Therefore, elucidating the underlying mechanisms of ICH and developing effective therapeutic strategies remain critical challenges that urgently need to be addressed.

Neutrophil extracellular traps (NETs) have recently been recognized as pivotal mediators driving neuroinflammation and secondary brain injury after ICH [15, 16, 17]. Excessive NET formation exacerbates inflammatory responses, disrupts the blood‐brain barrier (BBB), and impairs hematoma clearance, leading to unfavorable neurological outcomes [18, 19, 20]. Hence, suppressing NET‐triggered neuroinflammation represents a promising therapeutic strategy for ICH.

Exploring neuroprotective agents from natural products constitutes a promising strategy for the development of ICH therapeutics. In this study, a novel component, 2‐E‐feruloyloxybenzeneacetic acid ester (Efoba), was identified from aged garlic. To overcome the limited natural abundance of this compound for subsequent pharmacological studies, we developed a scalable chemical synthesis route. Through an integrated multi‐omics approach combining LiP‐MS, high‐throughput virtual screening, transcriptomics, and proteomics, we identified cathepsin C (CTSC) as a direct binding target of Efoba. CTSC serves as a key activator of neutrophil serine proteases (NE, MPO, and CitH3), which are essential for NETosis [21, 22, 23]. This makes CTSC a compelling upstream mediator linking microglial activation to NET‐related brain damage following ICH.

Given the established neuroprotective effects of aged garlic and the unmet demand for targeted anti‐inflammatory agents against ICH, this study systematically investigates the neuroprotective effects and molecular mechanism of Efoba. To elucidate the underlying mechanism, we adopt a systematic research framework integrating multi‐omics target screening, Transwell co‑culture assays, and in vivo validation using a mouse ICH model. Compared with previous investigations relying on crude garlic extracts, the present study not only achieves the isolation and structural identification of Efoba and establishes a stable, scalable synthetic route for this compound, but also identifies CTSC as the core functional target through which Efoba exerts neuroprotective effects. These findings provide crucial experimental evidence and a theoretical foundation for the translational application of neuroprotective agents originating from aged garlic.

## 2 Results

### 2.1 Discovery of the Novel Natural Ingredient Efoba

As a fermented product of garlic, the chemical components of aged garlic may undergo significant changes during the fermentation process. To compare the differences in metabolites between aged garlic and raw garlic, we first performed a non‐targeted metabolomics analysis (Figure 1A). Principal component analysis (PCA) revealed a clear separation between the garlic group and the aged garlic group, indicating distinct metabolic signatures (Figure 1B). Using the screening criteria of *p* < 0.05 and |log_2_FC| > 1, a total of 1030 differential metabolites were identified, among which 138 were down‐regulated, and 892 were up‐regulated in aged garlic compared to raw garlic (Figure 1C). The distribution of some significantly up‐regulated and down‐regulated differential metabolites is shown in Figure 1D. The types of up‐regulated and down‐regulated differential metabolites encompass various categories, including lipids, organic acids, alkaloids, and others (Figure 1E), indicating that aged garlic contains structurally diverse natural compounds. Kyoto Encyclopedia of Genes and Genomes (KEGG) pathway enrichment analysis of the differential metabolites revealed that these metabolites are significantly enriched in various metabolic pathways, including amino acid metabolism, terpenoid and quinone synthesis, and phenylpropanoid biosynthesis (Figure 1F). Furthermore, within the retention time range of 6–10 min, aged garlic exhibited more abundant chromatographic peaks compared to garlic, suggesting that the metabolic profiles within this interval are more diverse, with metabolites that are either unique to or present at higher abundance in aged garlic (Figure 1G).

**FIGURE 1:**
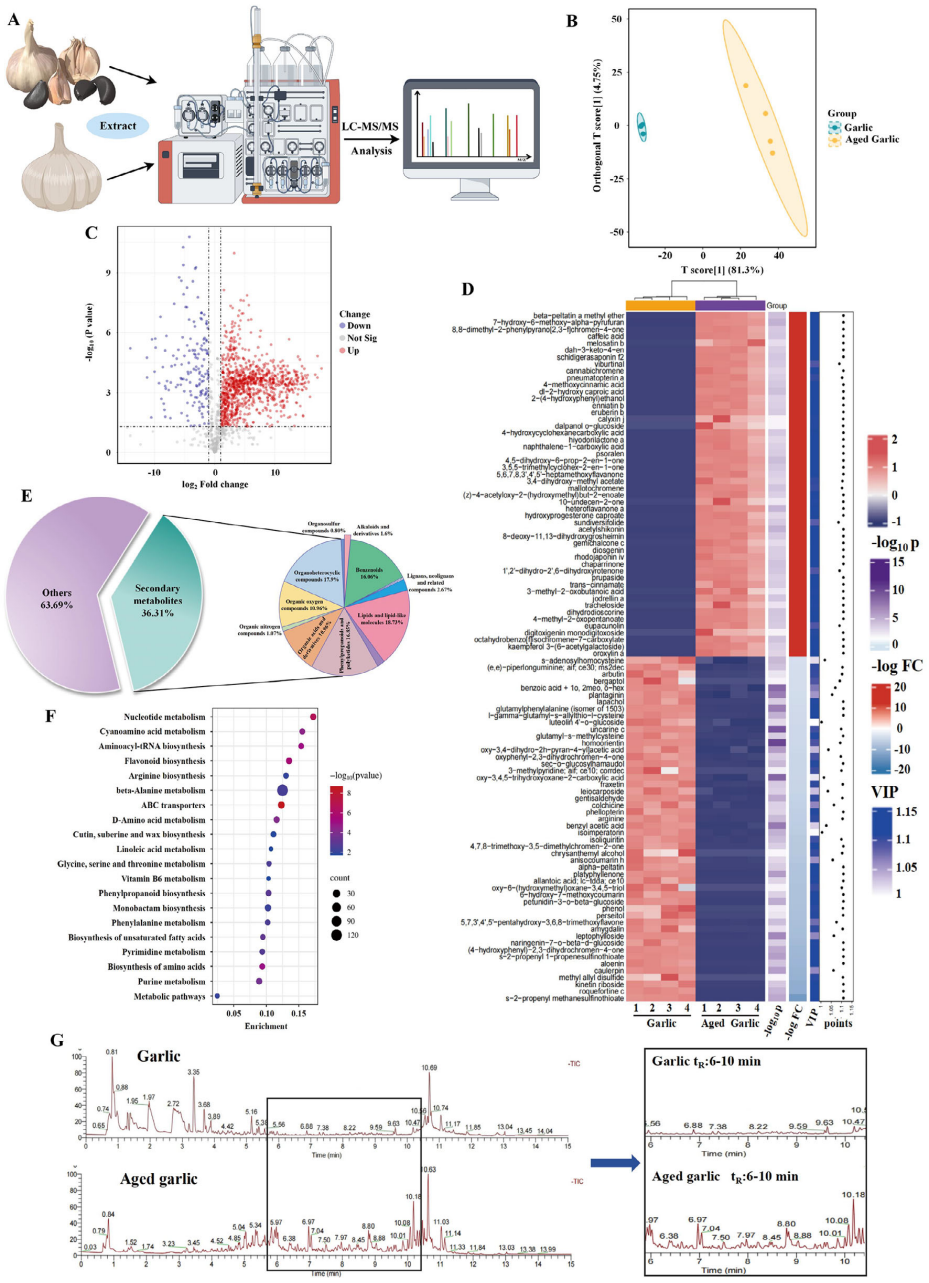
Differential metabolite analysis of aged garlic and garlic. (A) LC‐MS/MS‐based metabolomics workflow. (B) PCA analysis of the aged garlic group and the garlic group (n = 4 per group). (C) Volcano plot showing differences in metabolites between the black garlic and garlic groups (n = 4 per group). (D) Heatmap showing the distribution of differential metabolites between black garlic and garlic (n = 4 per group). (E) Classification of differential metabolites (n = 4 per group). (F) KEGG analysis of differential metabolites (n = 4 per group). (G) TIC (Total Ion Current) chromatograms of the aged garlic group and garlic group (n = 4 per group).

Based on the aforementioned differences, we systematically separated and purified the fractions enriched with differential metabolites from aged garlic using chromatographic methods. Following sequential silica gel column chromatography and semi‐preparative thin‐layer chromatography (TLC), a small‐molecule compound, named Efoba, with high purity was ultimately obtained via semi‐preparative liquid chromatography (Figure 2A). Its structurally related molecular data are presented in Figure S1. Efoba was obtained as an amorphous powder of pale yellow color. Its molecular formula was determined as C_18_H_15_O_6_ by HRESIMS (*m/z* 327.0869 [M‐H]^−^) in conjunction with NMR data, corresponding to eleven degrees of unsaturation (Figure S1A). The IR spectrum displayed characteristic absorption bands for hydroxy (3368, 3531 cm^−^^1^), ester carbonyl (1704 cm^−^^1^), and phenyl groups (1600, 1514 cm^−^^1^) (Figure S1B and Table S1). The chemical shifts and peak shapes of all protons are shown in Figure S1C. The ^1^ ^3^C NMR spectrum, with the aid of HSQC correlations, resolved 18 carbon resonances attributable to one methoxy (*δ*c 55.6), one methylene (*δ*c 65.7), nine olefinic/aromatic methines, and seven quaternary carbons (including three carbonyls at *δ*c 190.6, 166.0, and 163.7) (Figure S1D). After accounting for the unsaturations contributed by the carbonyl and olefinic groups, the remaining unsaturations indicated the presence of two benzene rings in the molecule. Analysis of the COSY spectrum identified three distinct spin systems: a trans‐configured double bond *δ*_H_ 7.60 (d, ^3^ *J*(H,H) = 15.9 Hz, H‐2) and 6.59 (d, ^3^ *J*(H,H) = 15.9 Hz, H‐3). An ABX system characteristic of a 1,3,4‐trisubstituted benzene ring *δ*_H_ 7.36 (br s, H‐5), 6.80 (d, ^3^ *J*(H,H) = 8.16 Hz, H‐8), and 7.15 (d, ^3^ *J*(H,H) = 8.16 Hz, H‐9), and an AA'BB' system indicative of a 1,4‐disubstituted benzene ring *δ*_H_ 7.83 (d, ^3^ *J*(H,H) = 8.58 Hz, H‐4'/H‐8') and 6.83 (d, ^3^ *J*(H,H) = 8.58 Hz, H‐5'/H‐7') (Figure S1E). The HSQC spectrum directly correlated these protons with their corresponding carbons (Figure S1F). The connectivity of the structural fragments was established by HMBC correlations (Figure S1G). The methoxy protons (*δ*_H_ 3.82) showed a correlation with *δ*c 147.9 (C‐6), confirming its attachment at the C‐6 position of the trisubstituted benzene ring. Correlations from H‐4'/H‐8' (*δ*_H_ 7.83) to the ketone carbonyl at *δ*c 190.6 (C‐2') confirmed the presence of a benzoyl moiety. The methylene protons H_2_‐1' (*δ*_H_ 5.45) exhibited key HMBC correlations to both the ester carbonyl at *δ*c 166.0 (C‐1) and the ketone carbonyl at *δ*c 190.6 (C‐2'), establishing a benzoyloxymethylene ester unit. Furthermore, the olefinic proton H‐3 (*δ*_H_ 7.60) correlated with C‐5 (*δ*c 111.2) and C‐9 (*δ*c 123.3) of the trisubstituted benzene ring, while H‐2 (*δ*_H_ 7.60) correlated with the ester carbonyl C‐1 (*δ*c 166.0). These connections demonstrated that the feruloyl moiety is linked via its carboxyl carbon to the oxygen atom of the methylene bridge, thereby connecting the feruloyl and benzoyl units (Figure 2B). Based on the vicinal coupling constant of 15.9 Hz between H‐2 and H‐3, which is characteristic of a trans configuration, the geometry of the double bond was determined to be E. The H‐H relationships in the structure were further analyzed by NOESY (Figure 2C and Figure S1H). Finally, the structure of Efoba was elucidated as 2‐E‐feruloyloxybenzeneacetic acid ester.

**FIGURE 2:**
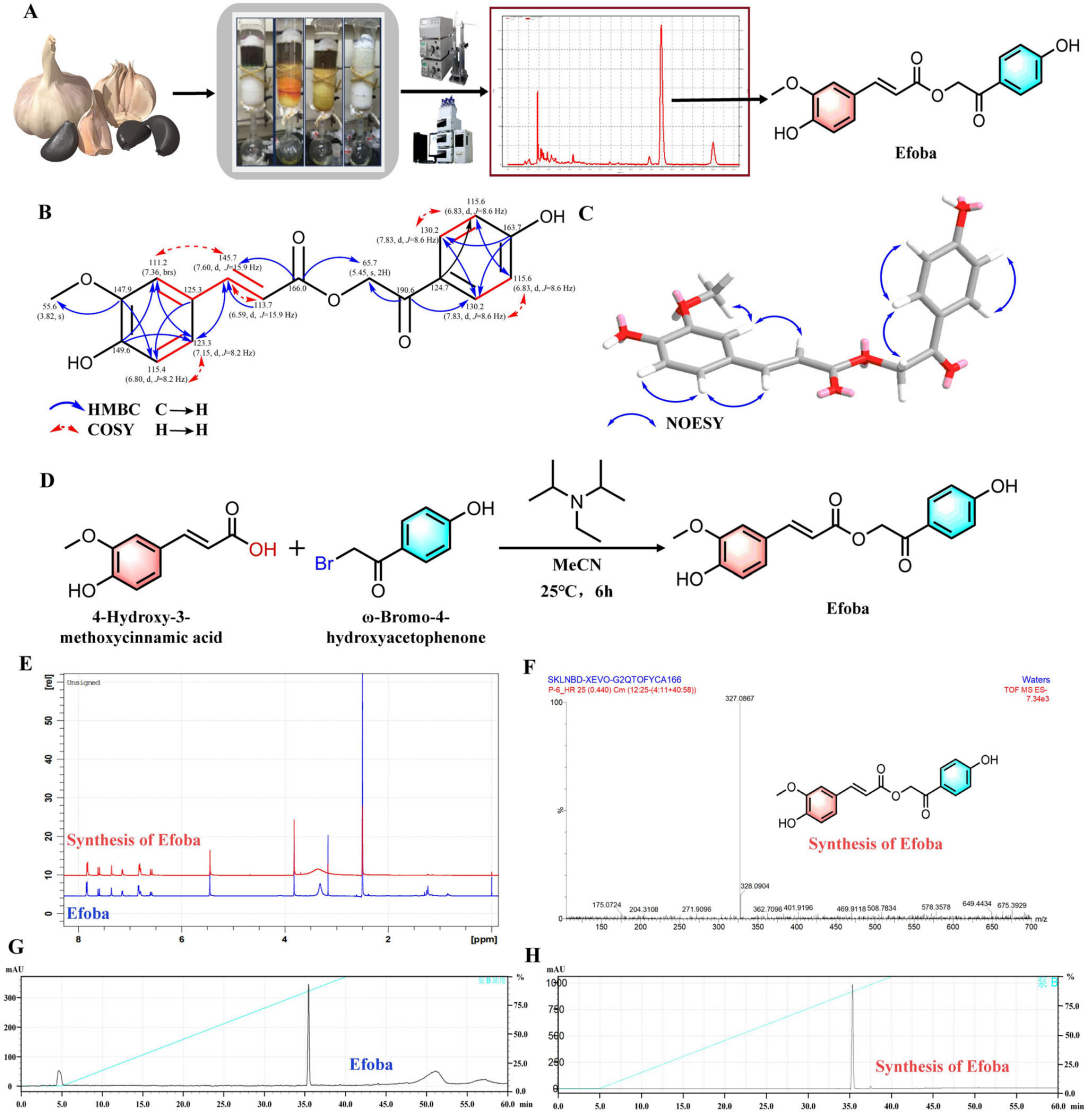
Isolation and chemical synthesis of Efoba. (A) Isolation, extraction, and purification of Efoba. (B) Key COSY and HMBC correlations of Efoba. (C) Key NOE relationships of Efoba. (D) Design of the chemical synthesis route for Efoba. (E) Comparison of ^1^H‐NMR spectra between naturally isolated and chemically synthesized Efoba. (F) Mass spectrometry analysis of synthesized Efoba. (G) HPLC chromatogram of naturally isolated Efoba for purity verification. (H) HPLC chromatogram of chemically synthesized Efoba for purity verification.

One of the major challenges in natural product research is the limited availability of active compounds from natural sources, which often restricts the amount of sample required for subsequent pharmacological studies and other investigations. To address this issue, we designed and developed a concise chemical total synthesis route for Efoba (Figure 2D). Through optimization of the molecular structure, systematic screening of reaction conditions, and continuous improvement of the separation and purification processes, the formation of by‐products was effectively reduced, and the reaction conversion rate was significantly enhanced. As a result, an efficient, robust, and scalable synthetic process was established. Currently, Efoba has been reliably prepared at a hundred‐gram scale with a purity exceeding 98%, and the structure was further confirmed by spectral comparison with naturally isolated Efoba, fully meeting the requirements for subsequent experimental studies (Figure 2E–H). The discovery of Efoba and the establishment of its synthetic system provide a solid material foundation for the subsequent mechanistic investigation and potential application development of this project.

### 2.2 Efoba, a Naturally Low‐Toxicity Compound Derived From Aged Garlic, Exerts Neuroprotective Effects in ICH Mice

Previous studies have demonstrated that aged garlic extract can alleviate neuroinflammation by regulating the NF‐κB signaling pathway [24]. To investigate whether its active component, Efoba, exerts a therapeutic effect in ICH, the safety of Efoba was first evaluated (Figure S2A). The experimental mice were divided into six groups: a control group and groups treated with Efoba at doses of 40, 80, 240, 480, and 960 mg kg^−1^. The acute toxicity results showed that no mortality or obvious signs of toxicity were observed in any of the treatment groups, indicating a favorable safety profile of the compound with no acute toxicity at the tested therapeutic concentrations. Furthermore, no significant differences in body weight were observed among all groups over the five‐week period (Figure S2B). At the end of the second week, mice from the control group and the 40, 480, and 960 mg kg^−1^ groups were selected, and hematological parameters were measured in the control group and Efoba‐treated groups. The vast majority of hematological parameters remained within normal physiological ranges across all dosage groups (Table S2). Serum biochemistry was applied to evaluate liver and kidney function (Table S3). Renal parameters including BUN, UA, and Cr showed no obvious abnormalities at all tested doses. Mild elevation of liver‐associated indices was observed at the highest doses of 480 and 960 mg kg^−1^, indicating mild hepatic stress under this extremely high dosage. The therapeutic dose adopted in our ICH experiments was far below this toxic dose, so no hepatotoxicity was expected under our experimental conditions. Coagulation profiles were determined in repeatedly dosed naive mice (Table S4). PT, APTT, TT, and FIB exhibited no significant changes after Efoba administration in mice. Further serum biochemical detection of circulating pro‑inflammatory cytokines (TNF‐α, IL‑6) revealed no obvious abnormalities among all groups. Collectively, these data indicate that Efoba exhibits low systemic toxicity and does not increase hemorrhagic risk under the present experimental conditions, suggesting that the dosages adopted in the present study are safe.

Then, various organs were collected for histopathological examination. The H&E staining results of the major organs from each group are presented in Figure S2C. Pathological analysis revealed that continuous administration of Efoba did not cause significant tissue abnormalities, including necrosis or inflammatory cell infiltration, in the examined organs (including the heart, liver, spleen, lung, kidneys, and brain). To clarify whether Efoba affects initial hematoma formation and hemorrhage volume, serial brain sections were prepared at 6 h after ICH induction for quantitative measurement of hematoma volume. No statistically significant difference in hematoma volume was found between the ICH group and the ICH+Efoba group (Figure S2D,E), confirming that Efoba does not aggravate early‐phase hemorrhage or alter the size of primary hematoma. After 14 consecutive days of administration, Efoba was administered to mice via tail vein injection, and its distribution in brain tissue was quantitatively detected using metabolomics technology. The results showed that Efoba could effectively cross the blood‐brain barrier and distribute into brain tissue (Figure S2F,G).

Next, we evaluated the neuroprotective effects of Efoba (Figure S3A). Administration of Efoba at three distinct concentrations all conferred therapeutic benefits in ICH mice, leading to varying degrees of improvement in survival status (Figure S3B). Moreover, Efoba dose‐dependently reduced hematoma volume in brain tissue and inhibited hematoma expansion (Figure 3A). Following ICH, compression by the hematoma can lead to cerebral ischemia and hypoxia, resulting in neurological damage. Cerebral blood flow monitoring results showed that blood flow in the cerebral cortex on the hemorrhagic side was significantly reduced in the ICH group and recovered slowly within 7 days. Efoba treatment significantly promoted the recovery of cortical blood flow and alleviated ischemia (Figure S3C,D). Histopathological changes in brain tissue were assessed using H&E and TUNEL staining. In the ICH group, a large number of homogeneous red substances (indicated by green circles) were observed in the brain tissue, along with neuronal shrinkage, nuclear pyknosis, and even disappearance. TUNEL staining revealed numerous apoptotic cells in the perihematomal region. Following treatment with different concentrations of Efoba, these pathological changes were significantly attenuated (Figure 3B).

**FIGURE 3:**
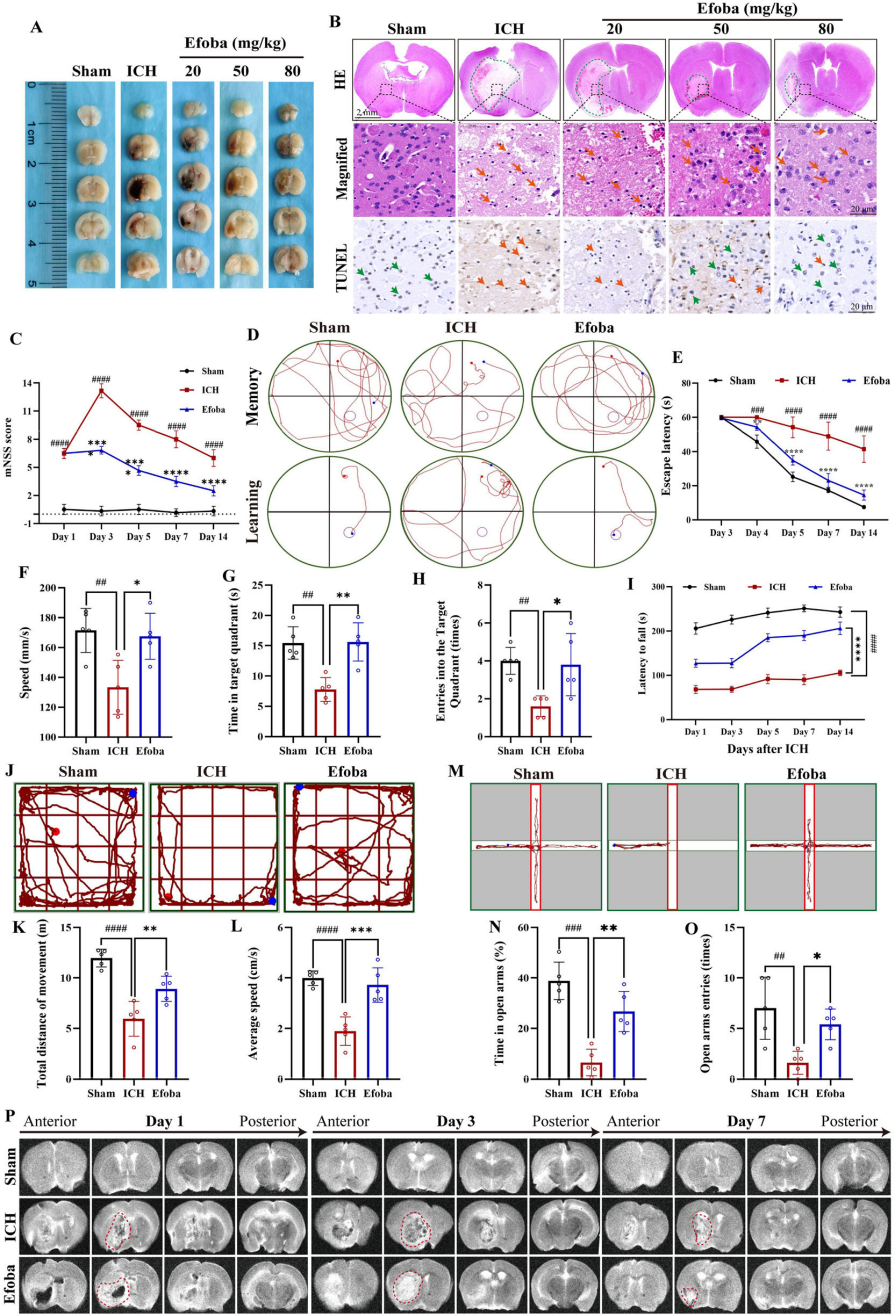
Neuroprotective effects of Efoba in ICH mice. (A) Observation of hematoma volume in brain tissue sections following ICH. (B) Evaluation of neuronal damage by H&E and TUNEL staining. H&E staining scale bar = 2 mm. H&E and TUNEL‐magnified apoptotic area. Scale bar = 20 µm. (C) Assessment of neurological deficits by mNSS scoring (n = 5 per group). (D–H) Morris water maze test demonstrating improved learning and memory abilities (n = 5 per group). (I) Rotarod test demonstrating improved motor coordination (n = 5 per group). (J–L) Open field test demonstrating amelioration of anxiety‐ and depression‐like behaviors (n = 5 per group). (M–O) Elevated plus maze test demonstrating amelioration of depression‐like behaviors (n = 5 per group). (P) MRI observation of hematoma resolution within 1–7 days post‐ICH. All Data are presented as mean ± SD. Statistical analysis was performed by one‐way ANOVA. ^#^ ICH group compared with control group; ^*^ Efoba‐treated group compared with ICH group. ^*/#^ *p* < 0.05, ^**/##^ *p* < 0.01, ^***/###^ *p* < 0.001, ^****/####^ *p* < 0.0001, “ns” means no significant difference (*p* >0.05).

Considering that ICH disrupts blood‐brain barrier integrity [25], we further assessed subsequent brain tissue damage and the therapeutic potential of Efoba in BBB protection. We examined the expression levels of tight junction proteins using western blotting. The results showed that, compared with the sham group, the expression of Occludin and ZO‐1 was significantly reduced 3 days post‐ICH, whereas Efoba treatment significantly increased the expression of these proteins (Figure S3E–G). To further validate the improvement in BBB function, we observed its ultrastructure using transmission electron microscopy. Representative images revealed that the tight junction structures in the perihematomal region were notably opened 3 days after ICH, a phenomenon that was markedly ameliorated in the Efoba‐treated group (Figure S3H). Concurrently, scanning electron microscope analysis also revealed that ICH induced myelin sheath damage (demyelination) and axonal injury (axonal atrophy). Efoba treatment significantly alleviated both types of damage, effectively promoting remyelination (as evidenced by a reduced g‐ratio) and preventing axonal atrophy, thereby restoring normal axonal diameter (Figure S3I–K).

To evaluate the overall behavioral performance of mice in each group, a series of behavioral tests were conducted. The modified Neurological Severity Score (mNSS), where higher scores indicate more severe impairment, was used to evaluate neurological function. ICH mice exhibited a significantly elevated mNSS, indicating severe deficits, whereas Efoba treatment markedly reduced this elevation, demonstrating significant neurological recovery (Figure 3C). The Morris water maze test showed that ICH mice exhibited significant impairments in spatial learning and memory, as evidenced by prolonged escape latency and reduced swimming speed during the training period, as well as decreased time spent in the target quadrant and fewer platform crossings during the probe trial. Efoba treatment significantly ameliorated these behavioral deficits (Figure 3D–H). The rotarod test demonstrated that ICH mice displayed marked deficits in motor coordination and balance, characterized by earlier fall latency and lower rotational speed compared to the sham‐operated group. In contrast, Efoba treatment significantly extended the time on the rotarod and increased the falling speed, indicating improved motor function (Figure 3I). ICH mice exhibited anxiety‐like and depression‐like behaviors, reflected by reduced central zone activity time, decreased total distance traveled, and lower locomotion speed in the open field test. Efoba intervention partially reversed these behavioral alterations, suggesting a recovery in spontaneous exploratory activity and emotional status (Figure 3J–L). Consistent with the open field results, ICH mice showed a significant reduction in both the number of entries into and the time spent in the open arms in the elevated plus maze test, further confirming the presence of anxiety‐like and depression‐like phenotypes. Notably, Efoba treatment effectively improved these parameters, indicating an anxiolytic‐like effect (Figure 3M–O). In summary, these behavioral results demonstrate that Efoba exerts multifaceted neuroprotective effects, ameliorating ICH‐induced deficits in spatial learning and memory, motor coordination, and emotional behavior.

Finally, dynamic T2‐weighted MRI was used to observe hematoma absorption in mouse brain tissue on days 1, 3, and 7 post‐ICH (Figure 3P). The results showed that within 3 days after ICH, severe hydrocephalus was observed, accompanied by a large hematoma volume and a slow absorption rate. After 7 days of Efoba treatment, hematoma was effectively controlled, absorption was accelerated, and the symptoms of intracerebral hemorrhage were significantly ameliorated. In combination with the measurement of brain tissue water content post‐ICH, the water content in the ipsilateral brain tissue was found to be elevated in the ICH group, whereas Efoba treatment alleviated this elevation relative to the ICH group. These findings further demonstrate that Efoba can mitigate brain tissue edema following ICH (Figure S3L).

### 2.3 Efoba Exerts Immunomodulatory Effects by Inhibiting Microglial Activation Following ICH

To investigate the mechanism underlying the neuroprotective effects of Efoba in ICH mice, RNA‐seq was performed on brain tissues from different groups of mice. The results showed that 1179 genes were significantly up‐regulated in the brain tissues after ICH (Figure S4A). KEGG enrichment analysis of these significantly up‐regulated genes revealed that they were mainly involved in immune‐related pathways, such as cytokine‐associated pathways, immune cell activation and inflammatory pathways, as well as chemotaxis and immune cell migration pathways (Figure S4B). The differentially expressed genes (DEGs) participating in immune pathways are shown in Figure S4C. After Efoba treatment, the DEGs are presented in Figure 4A; compared with the ICH group, 1239 genes were significantly down‐regulated (Figure 4B). The top enriched KEGG pathways with statistical significance (*p* < 0.05) are listed in Figure 4C, including cytokine–cytokine receptor interaction, NF‐κB signaling pathway, neutrophil extracellular trap formation, TNF signaling pathway, and lysosome, among others. Multiple core innate immune and pro‐inflammatory signaling pathways governing microglial polarization were significantly enriched in the down‐regulated gene set: NF‐κB, NOD‐like receptor, Toll‐like receptor, TNF and IL‐17 axes, as well as global cytokine‐receptor crosstalk. All these signaling cascades are well‐documented master regulators of microglial inflammatory responses following ICH. Transcriptomic analysis revealed broad transcriptional repression of these inflammatory signaling networks at the pathway level upon Efoba treatment, demonstrating that Efoba powerfully blunts microglial hyperactivation.

**FIGURE 4:**
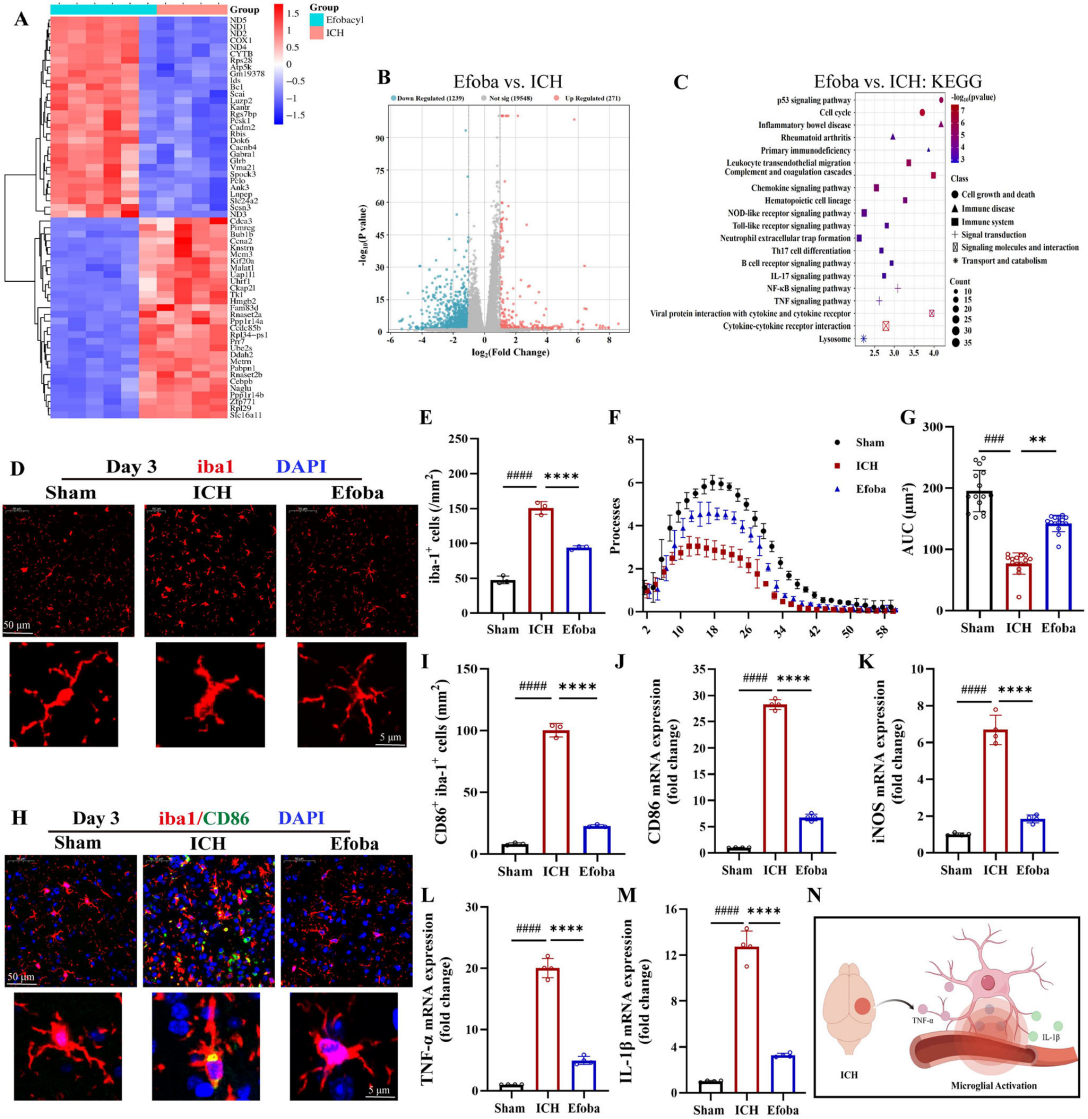
Efoba exerts immunomodulatory effects by inhibiting microglial activation following ICH. (A) Heatmap showing gene expression patterns across the Efoba group and ICH group based on mRNA sequencing data (n = 5 per group). (B) Volcano plot of differentially expressed genes between the Efoba group and ICH group (n = 5 per group). (C) KEGG enrichment analysis of differential genes between the Efoba group and ICH group (n = 5 per group). (D, E) Representative immunofluorescence staining for Iba1 (red) in the cortex at 3 days after ICH (n = 3 per group). Scale bar = 50 µm; scale bar = 5 µm (for magnified images). (F) Sholl analysis shows the mean number of microglial processes at distances from the cell center (n = 3 per group). (G) Area under the curve. (H, I) Representative double immunofluorescence staining for Iba1 (red) and CD86 (green) in the cortex at 3 days after ICH (n = 3 per group). Nuclei were stained with DAPI (blue). Scale bar = 50 µm; scale bar = 5 µm (for magnified images). (J–M) Pro‐inflammatory microglial activation‐like phenotype‐associated mRNA levels were evaluated, including CD86, iNOs, IL‐1β, and TNF‐α (n = 4 per group). (N) Schematic diagram of pro‐inflammatory microglial activation in brain tissue following ICH. All Data are presented as mean ± SD. Statistical analysis was performed by one‐way ANOVA. ^#^ ICH group compared with control group; ^*^ Efoba‐treated group compared with ICH group. ^*/#^ *p* < 0.05, ^**/##^ *p* < 0.01, ^***/###^ *p* < 0.001, ^****/####^ *p* < 0.0001, “ns” means no significant difference (*p* >0.05).

Transcriptomic findings indicated that Efoba represses excessive inflammatory activation of microglia after ICH. Since activated microglia secrete multiple chemotactic signals that drive recruitment of peripheral immune cells into brain parenchyma, we performed immunofluorescence staining with quantitative analysis to explore the effect of Efoba on post‑ICH immune‑cell infiltration. Substantial neutrophil infiltration was observed in brain tissues of ICH mice, and this phenotype was markedly attenuated upon Efoba treatment (Figure S4D,E). In normal brain tissue, microglia exhibited the most abundant branching and the most complex morphology. Following ICH, microglia were activated, displaying typical characteristics of pro‐inflammatory microglial activation, including a substantial reduction in branches, soma enlargement, and process retraction (Figure 4D,E). Efoba treatment inhibited the transformation of microglia into the activated state and partially preserved their branched morphology. To quantitatively assess the morphological changes of microglia, Sholl analysis was performed, and the area under the curve was calculated [26]. As shown in Figure 4F, the morphological distribution curve of the ICH group became short and flat compared with the high and broad peak‐shaped curve typical of the sham group, with the number of branches decreasing sharply at all radial distances. In striking contrast, the curve height and profile of the Efoba‐treated group were significantly restored, falling between those of the sham and ICH groups. Meanwhile, compared with the sham group, the area under the curve value of microglia in the ICH group was significantly decreased, indicating that the branch complexity and extension range were severely impaired, and the cell morphology transformed into the activated amoeboid form. After Efoba treatment, the AUC value was significantly increased relative to the ICH group (Figure 4G). Collectively, these results indicate that ICH induces severe process retraction of microglia, leading to their activation, whereas Efoba can effectively counteract this process and partially maintain the complex morphology of the cells.

Dual immunofluorescence staining and qPCR were employed to verify whether Efoba regulates microglial activation. The results showed that, compared with the sham group, microglia were significantly activated 3 days post‐ICH, with a substantial accumulation of double‐positive cells exhibiting colocalization of CD86 and Iba1, indicating polarization of microglia toward the pro‐inflammatory microglial activation‐like phenotype. Following Efoba administration, the number of activated microglia was significantly reduced, their soma morphology was restored, and the number of CD86^+^/Iba1^+^ double‐positive cells was significantly decreased (Figure 4H,I). qPCR results revealed that Efoba treatment significantly down‐regulated the mRNA expression levels of CD86 (Figure 4J), iNOS (Figure 4K), as well as the inflammation‐related factors TNF‐α (Figure 4L) and IL‐1β (Figure 4M). Additionally, we observed a significant increase in IL‐1β expression in microglia following ICH. However, compared with the ICH group, Efoba treatment markedly reduced the number of IL‐1β‐positive cells (Figure S4F,G). In summary, these findings demonstrate that microglia are activated after ICH and release pro‐inflammatory factors such as TNF‐α and IL‐1β, suggesting that microglia‐mediated neuroinflammation is a critical link in brain injury following ICH (Figure 4N). Furthermore, Efoba treatment effectively inhibits microglial activation and the subsequent neuroinflammatory response.

### 2.4 Efoba Exerts Neuroprotective Effects by Targeting CTSC

The proteomic results revealed that numerous proteins underwent significant changes following ICH, indicating that marked changes occurred in the mouse brain tissue after ICH (Figure S5A). Specifically, 265 proteins were significantly up‐regulated, and 104 proteins were significantly down‐regulated, suggesting that ICH induces extensive and substantial perturbations in brain protein expression, representing a core molecular feature of the pathological state (Figure S5B). GO functional enrichment analysis indicated a strong association with the immune system, revealing that catalytic metabolism and enzymatic regulation were robustly activated in brain tissue following ICH (Figure S5C). Differential proteins were significantly enriched in multiple pathways, including the complement and coagulation cascade, lysosome, and glutathione metabolism (Figure S5D). Following Efoba treatment, marked changes in protein expression were observed. The heatmap clearly distinguished the Efoba‐treated group from the ICH group, with altered clustering patterns, indicating that Efoba intervention effectively reversed ICH‐induced aberrant protein expression and exerted a significant regulatory effect (Figure S5E). Specifically, 123 proteins were significantly up‐regulated, and 157 proteins were significantly down‐regulated after Efoba treatment (Figure 5A). GO functional enrichment analysis showed that Efoba corrects ICH‐induced proteomic dysregulation and restores cellular homeostasis and metabolic balance by modulating core functions such as catalytic metabolism, substance transport, molecular binding, and enzymatic regulation (Figure S5F). KEGG enrichment analysis of differential proteins revealed that the complement and coagulation cascade was the most significantly enriched immune‐related pathway. The lysosome pathway was significantly enriched among Efoba‐suppressed proteins (Figure 5B). Meanwhile, according to the RNA‐seq data, the lysosome pathway was significantly enriched among Efoba‐suppressed genes (Figure 4F).

**FIGURE 5:**
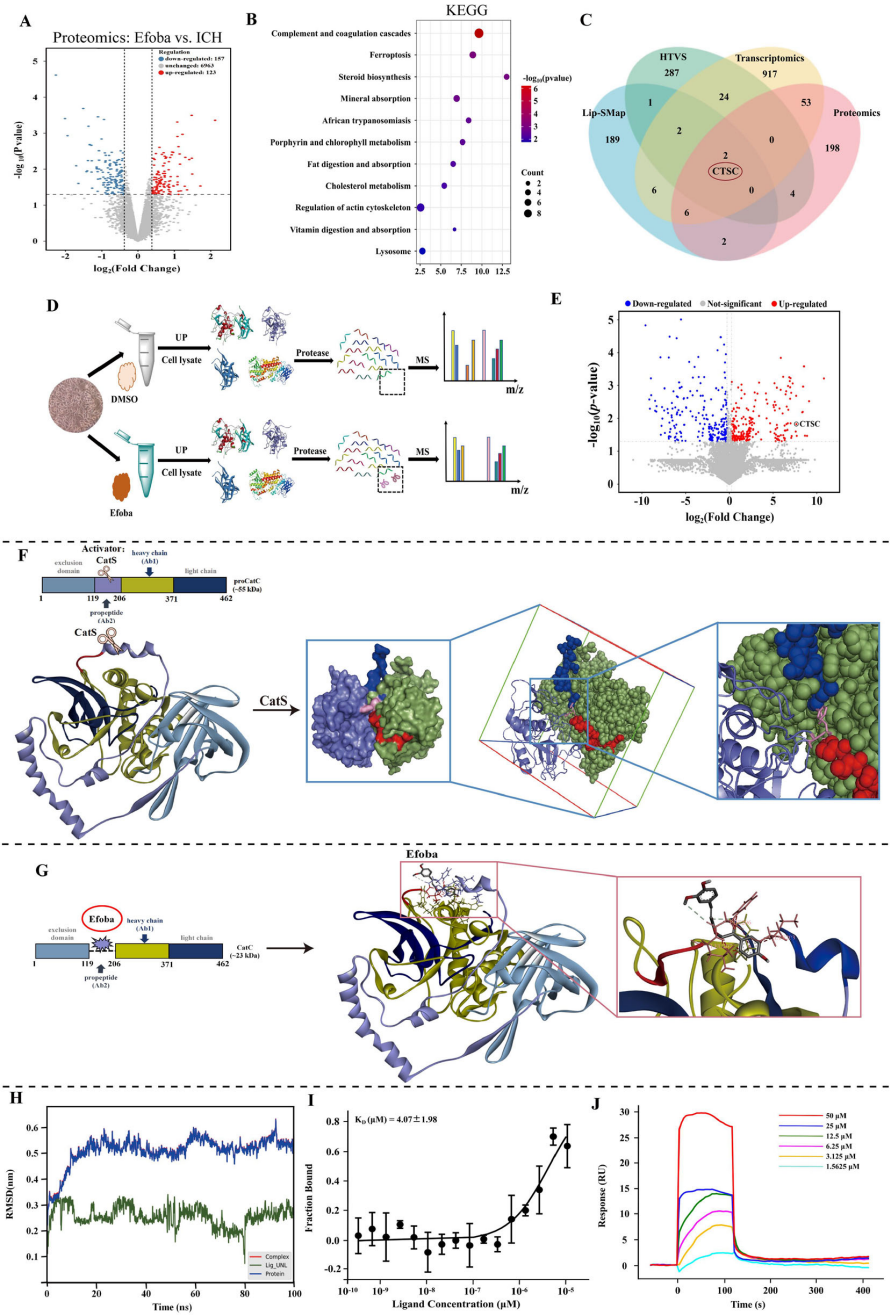
Target Identification and Validation of Efoba. (A) Volcano plot of differential proteins between the Efoba group and ICH group (n = 3 per group). (B) KEGG enrichment analysis of differential proteins between the Efoba group and the ICH group (n = 3 per group). (C) Intersection of common targets identified by multiple target‐finding methods. (D) Workflow for Lip‐MS. (E) Volcano plot of Lip‐MS results. (F) Binding site diagram of CatS (the activating enzyme of pro‐CTSC) on pro‐CTSC. (G) Efoba competes with CatS for the binding site on pro‐CTSC. (H) Molecular dynamics simulation results show that Efoba remains stable with the CTSC system. (I) MST analysis shows that the binding constant of Efoba with CTSC is 4.695 µм. (J) SPR shows that the binding constant of Efoba with CTSC is 1.79 µм. Data are shown as the mean values ± SD. Statistical significance was determined by ANOVA. ^#^ICH group compared with control group; ^*^ Efoba‐treated group compared with ICH group. ^*/#^ *p* < 0.05, ^**/##^ *p* < 0.01, ^***/###^ *p* < 0.001, ^****/####^ *p* < 0.0001, “ns” means no significant difference (*p* >0.05).

To identify the molecular targets through which Efoba exerts its neuroprotective effects, we employed Limited Proteolysis‐Mass Spectrometry (Lip‐MS) to detect protein‐small molecule interactions by monitoring changes in the local protease susceptibility of proteins induced by small molecule binding. This was combined with high‐throughput virtual screening (HTVS), RNA‐seq, and proteomic analysis to search for potential targets. We generated a Venn diagram for candidate targets identified via multiple screening approaches, and two intersecting targets, CTSC and CTSS, were obtained (Figure 5C). CTSC and CTSS are lysosomal resident proteases [27]. According to LiP‐MS results, CTSC showed more pronounced alterations than CTSS (Figure 5D,E). CTSC exhibited the most robust binding signal in LiP‐MS (Fold Change = 166.75, *p* = 0.0157) and the highest predicted affinity in HTVS (similarity score = 0.6328, predicted activity = 790 nM), compared to CTSS (Fold Change = 3.85, *p* = 0.0456, similarity score = 0.6154, predicted activity = 1600 nM) (Tables S1 and S2). Proteomic data revealed that Efoba treatment reversed the protein abundance of CTSC (log_2_FC = −0.658, *p* = 0.0338), while CTSS showed no significant alteration at the protein level upon Efoba intervention (log_2_FC = −0.578, *p* = 0.164). RNA‐seq data revealed distinct transcriptional regulatory patterns for these two genes. At the transcriptomic level, Efoba treatment significantly reversed the ICH‐induced upregulation of both CTSC (log_2_FC = −1.1941, *p* = 5.44e‐05) and CTSS (log_2_FC = −0.737, *p* = 0.0439). Therefore, based on the above‐mentioned comparison of screening parameters, CTSC was selected as the target for further investigation.

Given its significant changes in the aforementioned analyses, we hypothesized that Efoba may exert neuroprotective effects by targeting CTSC. Protein structure analysis indicated that the differential peptides were located within the activation region of the CTSC precursor (pro‐CatC), a region regulated by cathepsin S (CatS) (Figure 5F). Molecular docking simulations suggested that Efoba binds to this site, thereby hindering CatS‐mediated activation of pro‐CatC and inhibiting the release of mature CTSC (Figure 5G). To validate this interaction, we further performed molecular dynamics simulations, surface plasmon resonance, and microscale thermophoresis (MST) assays. Molecular dynamics simulations showed that the Root Mean Square Deviation (RMSD) of the Efoba‐CTSC complex remained stable over a 100 ns simulation period, indicating good stability of the binding conformation (Figure 5H). MST experiments confirmed that Efoba directly binds to CTSC in lysates of OxyHb‐induced BV2 cells (Figure 5I). SPR analysis further demonstrated that Efoba binds to CTSC protein in a concentration‐dependent manner under in vitro conditions, with a dissociation constant of 1.79 µм (Figure 5J). Collectively, these multiple lines of evidence consistently demonstrate that Efoba exerts its neuroprotective effects against ICH by directly binding to CTSC and inhibiting its activation.

### 2.5 Efoba Inhibits the Release of Microglia‐Derived CTSC by Stabilizing the Lysosomal Membrane

Based on the above findings, we detected the expression changes of CTSC in mouse brain tissue following ICH. The results showed that CTSC expression was significantly upregulated in the brain of ICH mice, whereas this upregulation was markedly suppressed by Efoba treatment (Figure 6A,B). To further precisely identify the cellular source of CTSC, we performed immunofluorescence co‑staining using cell‑type‑specific markers covering multiple central nervous system cell populations. After ICH, prominent CTSC co‑localization was exclusively observed in TMEM119^+^ resident microglia. By contrast, negligible overlap was detected between CTSC and MPO^+^ neutrophils, CD31^+^ vascular endothelial cells, Olig2^+^ oligodendrocytes, NeuN^+^ neurons, or GFAP^+^ astrocytes (Figure 6C). Linear scanning of fluorescence intensity along the designated tissue path yielded highly overlapping intensity profiles for TMEM119 and CTSC signals (Figure 6D). Quantitative mean analysis verified that microglia exhibited substantially higher CTSC levels compared with neutrophils, vascular endothelial cells, oligodendrocytes, astrocytes, and neurons (Figure 6E), further corroborating widespread co‑localization between microglial signals and CTSC. Immunohistochemistry (IHC) staining showed that the ICH group exhibited abundant brown CTSC‐positive staining in the hemorrhagic core and perihematomal regions, with dense signals and high intensity. In contrast, CTSC‐positive staining was significantly reduced in the Efoba‐treated group, with only sparse distribution and markedly weaker intensity compared to the ICH group (Figure S6A). Collectively, these in vivo data demonstrate that activated resident microglia are the predominant cellular source of elevated CTSC in ICH‑injured brain tissue.

**FIGURE 6:**
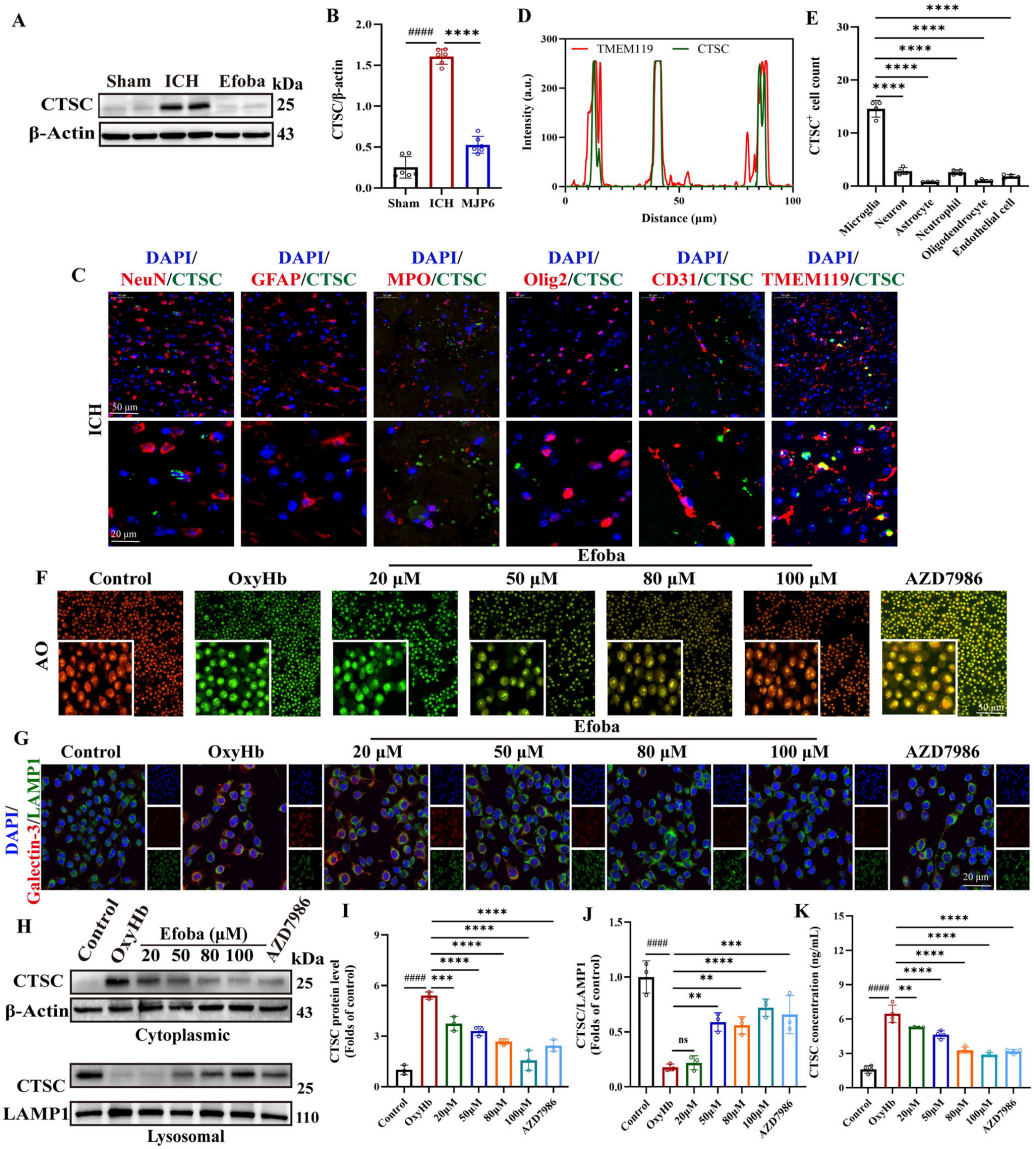
CTSC is enriched in microglia and CTSC secretion through LMP. (A,B) Western blot analysis of the expression of CTSC in mouse brain after ICH (n = 3 per group). (C–E) Representative double immunofluorescence staining for neurons (red), astrocytes (red), MPO (red), Olig2 (red), CD31 (red), TEME119 (red), and CTSC (green) in the cortex at 3 days after ICH (n = 3 per group). Nuclei were stained with DAPI (blue). Scale bar = 50 µm; scale bar = 20 µm (for magnified images). (F) Representative AO staining images of BV2 cells. Scale Bar = 50 µm. (G) Representative immunofluorescence images depicting markers for lysosomes (LMP1) co‐localized with Galectin‐3 in BV2 cells. Scale Bar = 20 µm. (H–J) Protein levels of CTSC in the cytoplasm and lysosome of BV2 cells were detected using western blot (n = 3 per group). (K) Concentrations of CTSC in the supernatant of BV2 cells were measured using ELISA (n = 4 per group). Data are shown as the mean ± SD. Statistical significance was determined by ANOVA. ^#^ ICH group compared with control group; ^*^ Efoba‐treated group compared with ICH group. ^*/#^ *p* < 0.05, ^**/##^ *p* < 0.01, ^***/###^ *p* < 0.001, ^****/####^ *p* < 0.0001, “ns” means no significant difference (*p* > 0.05).

To further investigate the secretion mechanism of CTSC in microglia, we first assessed the cytotoxicity of Efoba on microglia using the MTT assay. The results showed that within a certain concentration range, Efoba had minimal impact on cell viability, confirming its low toxicity (Figure S6B,C). Immunofluorescence analysis revealed that CTSC expression was significantly elevated in the OxyHb group, whereas Efoba inhibited its expression in a concentration‐dependent manner (Figure S6D,E). RNA‐seq results indicated that CTSC was significantly up‐regulated following ICH. In the ICH group compared to the sham group, cellular component (CC) analysis of the up‐regulated genes revealed enrichment in terms related to lysosomes and neutrophil chemotaxis. CTSC was identified as a differential gene associated with these terms (Figure S5C). Based on the lysosomal localization of CTSC, we subsequently investigated lysosomal membrane integrity and CTSC release in BV2 cells.

Acridine orange (AO) staining was employed to assess lysosomal membrane permeability (LMP). Under conditions of intact lysosomal membranes, AO emits orange‐red fluorescence, whereas upon LMP, distinct green fluorescence becomes visible in the cytoplasm [28]. Following OxyHb induction, the OxyHb‐induced cells exhibited significantly enhanced green fluorescence, indicating exacerbated LMP. In contrast, the Efoba‐treated group predominantly displayed orange‐red fluorescence, suggesting maintained lysosomal membrane stability (Figure 6F). To further validate the occurrence of LMP, we examined the colocalization of Galectin‐3, a well‐established marker of lysosomal membrane damage [29]. Upon LMP, exposure of N‐acetylglucosamine residues on the lysosomal lumenal membrane promotes the recruitment of Galectin‐3, leading to its increased co‐localization with the lysosomal membrane marker LAMP1, as visualized by fluorescence microscopy. The OxyHb‐induced cells showed significantly increased colocalization of Galectin‐3 and LAMP1, confirming the occurrence of LMP, whereas Efoba treatment effectively reduced their colocalization (Figure 6G). By isolating lysosomal and cytosolic protein fractions, we confirmed that lysosomal rupture following ICH led to CTSC leakage into the cytoplasm. Efoba treatment preserved lysosomal membrane integrity and reduced the release of CTSC into the cytoplasm (Figure 6H–J). ELISA results further demonstrated that Efoba inhibited CTSC secretion (Figure 6K). Additionally, AZD7986, a specific CTSC inhibitor, was used as a positive control to further validate the effective inhibitory effect of Efoba on CTSC.

### 2.6 CTSC Expression is Associated With NET Formation Following ICH

To identify key pathological pathways activated in the brain following ICH, we conducted RNA‐seq, spatial proteomics, and scanning electron microscopy (Figure 7A). RNA‐seq KEGG enrichment analysis revealed that the neutrophil extracellular trap formation pathway was significantly up‐regulated after ICH. GSEA further revealed a significant enrichment of the neutrophil extracellular trap formation gene set in the ICH group (NES = 2.12, FDR < 0.05), indicating that this pathway plays a crucial role in the pathological process of ICH (Figure 7B) and suggesting increased NET formation in the brain following ICH. Analysis of the leading‐edge genes from this pathway highlighted CTSC as one of the most prominently up‐regulated genes (Figure 7C), suggesting a strong correlation between CTSC upregulation and the induction of NET formation. SEM observation of brain tissue provided ultrastructural evidence supporting this conclusion, revealing abundant NETs in the brain after ICH (Figure 7D). These results collectively indicate that the immune‐inflammatory response triggered by ICH significantly promotes NET formation.

**FIGURE 7:**
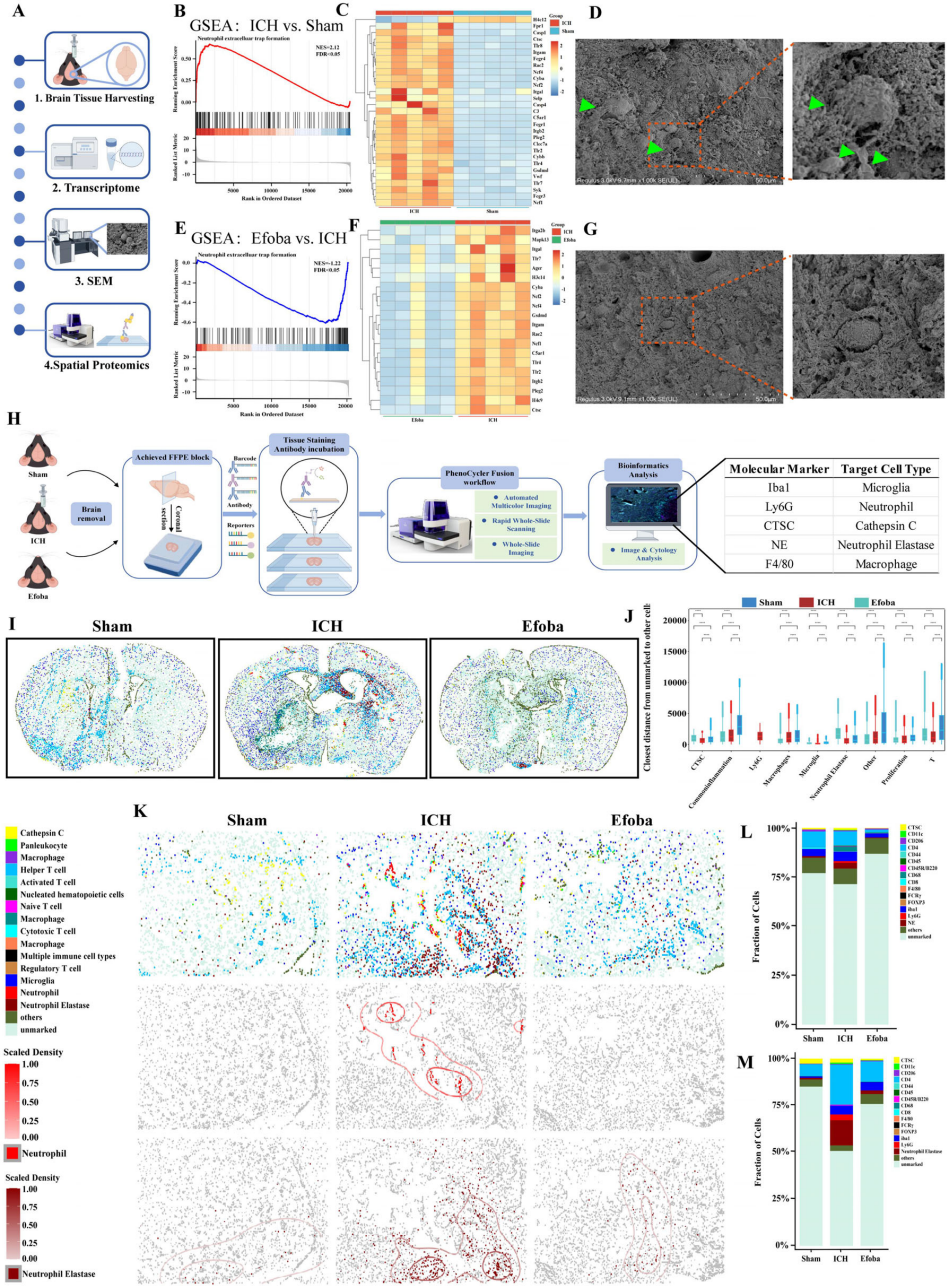
Association between CTSC expression and NET formation following ICH. (A) Experimental workflow. (B) Gene set enrichment analysis (GSEA) of brain tissue after ICH between ICH group and sham group (n = 5 per group). (C) Heatmap of differentially expressed genes (DEGs) in brain tissue after ICH between the ICH group and the sham group (n = 5 per group). (D) Scanning electron microscopy (SEM) images of brain tissue from the ICH group. (E) GSEA of brain tissue between Efoba group and ICH group (n = 5 per group). (F) Heatmap of DEGs in brain tissue after ICH between Efoba group and ICH group (n = 5 per group). (G) SEM images of brain tissue from the Efoba group. (H) Spatial proteomics workflow. (I) Representative spatial distribution of cellular populations in brain tissue across experimental groups. (J) Quantitative spatial abundance of molecular markers in brain tissue. (K) Representative distribution of biomarkers and NETs in selected brain region. (L) Proportion of various cellular markers in the entire brain tissue. (M) Proportion of various cellular markers in the selected brain region. Data are shown as the mean values ± SD. Statistical significance was determined by ANOVA. ^*/#^ *p* < 0.05, ^**/##^ *p* < 0.01, ^***/###^ *p* < 0.001, ^****/####^ *p* < 0.0001, “ns” means no significant difference (*p* > 0.05).

In the Efoba‐treated group, RNA‐seq analysis showed that pathways associated with NETs were significantly suppressed. GSEA revealed a significant downregulation of the Neutrophil extracellular trap formation gene set compared to the ICH group (NES = −1.22, FDR < 0.05), indicating that Efoba suppresses this pathological pathway (Figure 7E). Examination of the leading‐edge genes within this gene set showed that CTSC was among the significantly down‐regulated genes (Figure 7F), suggesting that the downregulation of CTSC may contribute to the inhibition of NET formation by Efoba. SEM analysis of brain tissue further confirmed that Efoba effectively reduced the formation of NETs at the ultrastructural level (Figure 7G). In summary, Efoba effectively suppresses NET formation by downregulating CTSC expression.

To further elucidate the spatial cellular and molecular characteristics following ICH in situ, we employed PhenoCycler spatial proteomics technology for multiplexed in situ imaging of brain tissue sections, constructing a spatial proteomic atlas of the mouse brain after ICH. This atlas enabled the comparison of cellular composition, distribution, and intercellular interactions across different regions. Based on this atlas, we systematically identified multiple cell subsets, including stromal cells, immune cells, and epithelial cells (Figure 7H). Spatial distribution analysis revealed a marked disorganization of cellular architecture following ICH. Abundant immune cell infiltration, predominantly neutrophils, was observed in the brain parenchyma, accompanied by NET formation and the development of focal inflammatory lesions. Following Efoba treatment, both the extent and density of immune cell infiltration were significantly reduced, along with decreased expression of NET‐associated markers, indicating that Efoba effectively suppresses ICH‐induced inflammatory cell recruitment and NET formation (Figure 7I,L). Molecular colocalization analysis demonstrated that the spatial distances among CTSC, neutrophil elastase (NE), and the neutrophil marker Ly6G were significantly shortened in the ICH group, indicating a high degree of colocalization of these molecules in the post‐ICH brain and suggesting a close spatial coupling between neutrophil activation and CTSC release. After Efoba intervention, the spatial distances among these molecules were significantly increased, indicating disruption of the spatial colocalization associated with the CTSC‐NET pathway (Figure 7J). Further nearest neighbor distance analysis showed that the nearest neighbor distances of inflammation‐related molecules such as CTSC, NE, Ly6G, F4/80, and CD86 were significantly shortened in the ICH group, whereas Efoba treatment significantly restored and extended these distances (Figure S7A). Enrichment analysis revealed that the odds ratios associated with CTSC, CTSC‐iba1 colocalization, common inflammation, and NE‐Ly6G colocalization were significantly higher in the ICH group compared to the sham group, indicating a significant enrichment of these inflammatory pathways and neutrophil‐related molecules following ICH, constituting core pathological features (Figure S7B). Cellular subpopulation composition analysis further revealed a significant remodeling of the cellular landscape in the brain after ICH, with a substantial increase in the proportions of neutrophil‐related subpopulations (NE^+^, Ly6G^+^) and cells associated with common inflammation, forming a pro‐inflammatory immune cell‐centered pathological microenvironment. Efoba treatment significantly reduced the proportions of these inflammatory subpopulations, shifting the brain cellular composition back toward physiological homeostasis (Figure S7C). Additionally, the relative proportions of CTSC, neutrophils (Ly6G), and NE were significantly increased in the ICH group, whereas these proportions were markedly reduced in the Efoba group, further confirming that Efoba specifically inhibits CTSC expression, neutrophil activation, and aggregation (Figure 7K,M).

In summary, spatial proteomics analysis demonstrates that ICH induces a dramatic spatial redistribution of immune cells in the mouse brain. Neutrophil‐dominated inflammatory cells extensively infiltrate the brain and display strong spatial colocalization, building a pro‐inflammatory microenvironment centered on the CTSC‐NET pathway, which constitutes a key pathological mechanism underlying post‐ICH brain injury. Efoba exerts neuroprotective effects via three distinct aspects, restoring orderly cellular spatial distribution to mitigate neutrophil infiltration and aggregation, disrupting molecular spatial co‐localization by increasing the spatial distances between key molecules (CTSC, NE and neutrophil markers), thereby blunting CTSC‐NET pathway activation and remodeling cellular subpopulation composition by lowering the fractions of pro‐inflammatory immune subsets and returning brain tissue cellular composition toward physiological homeostasis. These findings provide direct spatial evidence that Efoba alleviates post‐ICH inflammatory injury and confers neuroprotection by suppressing neutrophil activation and blocking the CTSC‐NET pathway.

### 2.7 Efoba Confers Neuroprotection by Inhibiting Microglia‐Derived CTSC‐Mediated NET Formation

CTSC, as a key activator of neutrophil serine proteases (NSPs), promotes the activation of myeloperoxidase (MPO) and NE, thereby inducing citrullinated histone H3 (CitH3) and ultimately driving the NETosis process and NET formation. To investigate the role of microglia‐derived CTSC in mediating NETosis, we established a Transwell co‐culture system. BV2 cells stimulated with OxyHb were co‐cultured with mouse peripheral blood‐derived neutrophils. Unstimulated BV2 cells were used as the negative control, and the CTSC‑specific inhibitor AZD7986 was applied as a pharmacological control. Immunofluorescence results showed that OxyHb‐induced BV2 cells significantly promoted NETosis through high expression of CTSC, leading to extensive NET formation. This was evidenced by a marked increase in SYTOX Green‐positive NET structures and enhanced colocalization of MPO/CitH3 (Figure S8A), indicating substantial NET generation in the co‐culture system.

Subsequently, primary microglia isolated from mouse brain tissue were induced with OxyHb and co‐cultured with neutrophils. SYTOX Green staining revealed a significant increase in positive NET structures after co‐culture. SEM provided ultrastructural evidence of NET formation, revealing the release of typical web‐like and filamentous structures. As expected, Efoba markedly suppressed NET‐like structures (Figure 8A). Additionally, the release of CTSC into the culture system was inhibited by Efoba treatment (Figure 8B). Quantitative statistical analysis of MPO and CitH3 double‐positive signals further validated the decreased NET abundance following Efoba intervention (Figure 8C). Following Efoba intervention, the NETosis process was hindered, and NET formation was significantly reduced. Given that double‐stranded DNA (dsDNA) and MPO‐DNA from NETs can be released into the circulatory system, we measured their concentrations in the co‐culture system. Co‐culture of neutrophils with OxyHb‐induced microglia triggered robust neutrophil NETosis with increased dsDNA (Figure 8D) and MPO‐DNA complexes (Figure 8E), which was greatly attenuated under Efoba treatment. Co‐culture with neutrophils subsequently led to a marked increase in the expression of NET markers, including CitH3 and PAD4. Western blot analysis showed that Efoba treatment effectively suppressed the expression of CitH3 and PAD4 proteins (Figure 8F–H).

**FIGURE 8:**
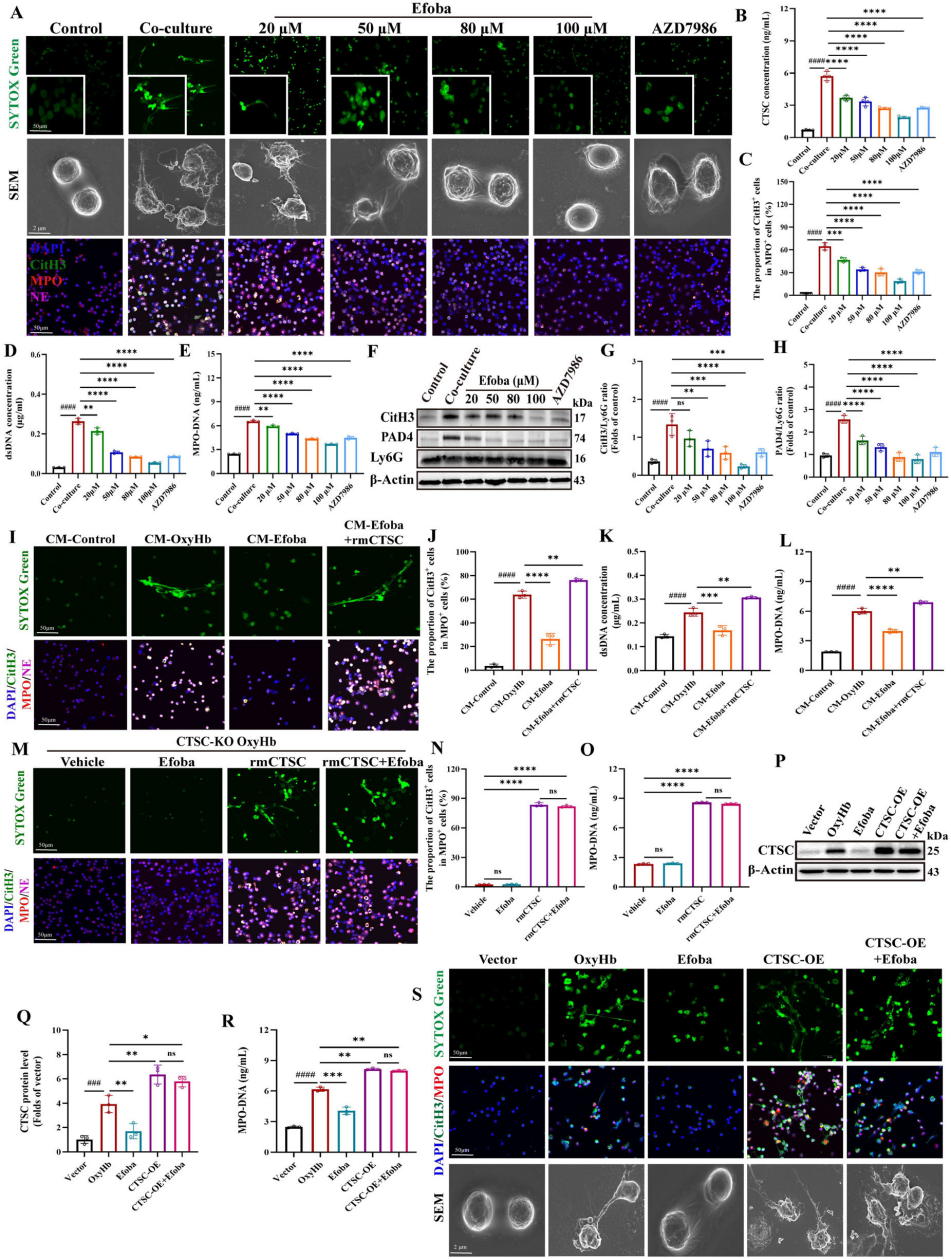
Efoba confers neuroprotection by inhibiting microglia‐derived CTSC‐mediated NET formation. (A,C) Representative images of NET formation stained with SYTOX Green, MPO (red), CitH3 (green), and NE (pink). Nuclei were counterstained with DAPI (blue). Scale bars: 50 µm (overview) and 2 µm (magnified, SEM images). (B) The levels of CTSC in the co‐cultured cells were detected using ELISA. (D) The levels of dsDNA in the co‐cultured cells were detected using ELISA. (E) The levels of MPO‐DNA in the co‐cultured cells were detected using ELISA. (F–H) Protein levels of CitH3, Ly6G, and PAD4 were detected using Western blot. (I,J) Representative images of NET formation stained with SYTOX Green, MPO (red), CitH3 (green), and NE (pink) from conditioned medium (CM) experiments. Nuclei were counterstained with DAPI (blue). Scale bars: 50 µm (overview). (K) The levels of dsDNA in the CM experiments were detected using ELISA. (L) The levels of MPO‐DNA in the CM experiments were detected using ELISA. (M,N) Representative images of NET formation stained with SYTOX Green, MPO (red), CitH3 (green), and NE (pink) of CTSC‐KO BV2 cells. Nuclei were counterstained with DAPI (blue). Scale bars: 50 µm (overview). (O) The levels of MPO‐DNA in the CTSC‐KO BV2 cells were detected using ELISA. (P,Q) Detection of CTSC in CTSC‐KO BV2 cells by Western blot. (R) The levels of MPO‐DNA in the CTSC‐KO BV2 cells were detected using ELISA. (S) Representative images of NET formation after CTSC overexpression, stained with SYTOX Green, MPO (red), and CitH3 (green). Nuclei were counterstained with DAPI (blue). Scale bars: 50 µm (overview) and 2 µm (magnified, SEM images). Data are shown as the mean ± SD (n = 3). Statistical significance was determined by ANOVA. # ICH group compared with control group; ^*^ Efoba‐treated group compared with ICH group. ^*/#^ *p* < 0.05, ^**/##^ *p* < 0.01, ^***/###^ *p* < 0.001, ^****/####^ *p* < 0.0001, “ns” means no significant difference (*p* >0.05).

To determine whether Efoba suppresses NETosis by acting on microglia or directly on neutrophils, we performed neutrophil monoculture experiments in the absence of microglia. Purified primary neutrophils were stimulated with PMA (a classic NETosis inducer) and OxyHb at the same concentration used in the co‐culture system, with gradient concentrations of Efoba administered for intervention. CCK‐8 assays revealed that Efoba exerted no obvious cytotoxic effect on neutrophil viability (Figure S8C). CCK‐8 assay showed that PMA stimulation induced a slight reduction in overall neutrophil viability relative to untreated controls (Figure S8D). No significant difference in cell viability was detected between the PMA group and the Efoba group. PMA stimulation triggered robust NETosis in neutrophils, accompanied by elevated ROS production. Efoba dose‐dependently inhibited PMA‐induced NETosis, as evidenced by reduced extracellular dsDNA, MPO‐DNA complexes, MPO/CitH3 colocalization, and SYTOX Green‐positive NET‐like structures (Figure S8B,E,F). These findings suggest that Efoba suppresses NET formation without exerting cytotoxic effects on neutrophils. However, directly stimulating isolated neutrophils with OxyHb at the exact same concentration as that used in the co‐culture system failed to elicit canonical NETosis in purified neutrophils. No obvious CitH3‐positive signals were observed after direct OxyHb treatment. Consistently, hardly any SYTOX Green‐positive extracellular NETs‐like structures or MPO/CitH3 double‐positive signals were detected, and the levels of extracellular dsDNA and MPO‐DNA complexes were not significantly elevated (Figure S8G–I).

To further dissect the primary cellular target of Efoba, we conducted compartment‐specific pretreatment washout co‐culture assays and conditioned medium (CM) rescue experiments. Microglia or purified neutrophils were pre‐incubated with Efoba, followed by thorough washing to remove residual drug before establishing the co‐culture system. Three pretreatment groups were set: microglia‐only pretreatment, neutrophil‐only pretreatment, and combined pretreatment of both cell types. When microglia were pre‐incubated with Efoba and stimulated with OxyHb, the medium containing drugs was subsequently washed out before co‐culture with neutrophils; SYTOX Green positive extracellular mesh‐like structures (Figure S9A), MPO/CitH3/NE colocalization (Figure S9A,B), dsDNA (Figure S9D), and MPO‐DNA (Figure S9E) were significantly suppressed. The concentration of secreted CTSC was markedly decreased in the supernatant from Efoba‐pretreated microglia (Figure S9C). By contrast, abundant NETs were still observed when only neutrophils received Efoba pretreatment. The inhibitory effect in the combined pretreatment group was comparable to that of the microglia‐only pretreatment group. In contrast, Efoba pretreatment limited exclusively to neutrophils exerted no obvious effects on these NETosis‐associated proteins. These results suggest that microglia are the predominant cellular target of Efoba, while its direct action on neutrophils only partially mediates the overall suppression of NETosis.

In conditioned medium experiments, CM‐OxyHb (supernatant collected from OxyHb‐challenged microglia) efficiently induced NETosis in primary neutrophils. CM‐Efoba (culture supernatant from Efoba‐treated microglia) exhibited a greatly diminished capacity to trigger NET formation. Critically, exogenous supplementation of recombinant rmCTSC into CM‐Efoba fully restored NET‐associated phenotypes, including elevated SYTOX Green positive extracellular NET‐like structures (Figure 8I), recovery of dsDNA (Figure 8K) and MPO‐DNA complex levels (Figure 8L), and restored CitH3/MPO/NE signals (Figure 8I,J). These findings further confirmed that Efoba suppresses NETosis primarily by targeting microglia and reducing the secretion of microglia‐derived CTSC.

To determine whether Efoba inhibits NET formation via CTSC, we generated CTSC‐knockout (CTSC‐KO) BV2 microglia (Figure S9F,G) and performed Transwell co‐culture with primary neutrophils under OxyHb stimulation. In the co‐culture system consisting of CTSC‐KO cells and neutrophils, OxyHb stimulation failed to induce robust NETosis. SYTOX Green‐positive extracellular‐like structures (Figure 8M), CitH3/MPO/NE positive signals (Figure 8M,N), and MPO‐DNA complexes (Figure 8O) remained at low levels. Under CTSC‐deficient conditions, Efoba treatment produced no additional inhibitory effect on NETosis compared with the vehicle group. Nevertheless, supplementation of recombinant rmCTSC into the co‐culture system effectively restored vigorous NETosis, manifested by abundant SYTOX Green‐positive structures and significantly elevated MPO‐DNA complex levels. Notably, rmCTSC‐mediated NET induction could not be reversed by Efoba; no statistically significant differences in any NET‐related readouts were observed between the rmCTSC group and the rmCTSC+Efoba group.

Then, we constructed a CTSC overexpression lentiviral vector and transfected it into BV2 cells. Western blot analysis confirmed a significant increase in CTSC expression following transfection (Figure 8P,Q). BV2 cells overexpressing CTSC were induced and then co‐cultured with neutrophils, with empty vector‐transfected cells serving as controls. Colocalization of MPO and NE confirmed the involvement of neutrophil‐derived proteins in NET composition, indicating the initiation of NETosis (Figure S9H). SYTOX Green staining, MPO/CitH3 colocalization, SEM ultrastructural images (Figure 8S), and MPO‐DNA complex observations (Figure 8R) revealed that NETosis was significantly enhanced in the CTSC overexpression group compared to the empty vector control group, indicating that CTSC overexpression alone is sufficient to induce robust NET production. In the empty vector control group, Efoba treatment significantly inhibited NETosis. However, in the CTSC overexpression group, the inhibitory effect of Efoba was significantly reversed, with NET levels showing no statistical difference compared to the untreated group. SEM further demonstrated abundant web‐like NET structures under CTSC overexpression conditions, which were not effectively suppressed by Efoba intervention. These results indicate that CTSC overexpression antagonizes the inhibitory effect of Efoba on NETosis, demonstrating that the function of Efoba is CTSC‐dependent and that its mechanism of action is achieved through inhibition of CTSC activity.

### 2.8 Efoba Targets CTSC to Suppress NET Formation in ICH

To verify whether Efoba inhibited NET formation by targeting CTSC, we established the ICH model and administered gradient doses of Efoba and the CTSC‐specific inhibitor AZD7986 (Figure 9A). To characterize the temporal dynamics of CTSC and NET‐related markers following ICH, brain tissue was collected at 2 h, 24 h, 48 h, 72 h, and 7 days post‐ICH for analysis. ELISA results showed that CTSC secretion levels exhibited dynamic changes over time (0h‐7d), beginning to increase at 2 h, peaking between 48 h and 3 days, and remaining elevated at 7 days (Figure S10A). Western blot analysis revealed that the expression of CitH3 and Ly6G was significantly up‐regulated from day 1 to day 3 post‐ICH, with a subsequent decline by day 7 (Figure S10B–D). Western blot analysis of peri‑hematoma brain tissues revealed markedly elevated protein levels of CTSC, CitH3 and PAD4 after ICH injury. Efoba treatment dose‑dependently reduced the abundance of CTSC, CitH3, and PAD4, with comparable inhibitory effects observed in the AZD7986‑treated group (Figure 9B–E). Immunofluorescence staining for CitH3/MPO/NE further demonstrated abundant NET‐like structures in the peri‑hematoma region of ICH mice, which were obviously diminished following Efoba or AZD7986 intervention (Figure 9F,G). We quantified the proportion of CitH3‑positive cells among all Ly6G‑positive neutrophils, which reflects the percentage of neutrophils undergoing NET formation. As shown in Figure 9F,H, the proportion of CitH3^+^ cells within Ly6G^+^ neutrophils was markedly elevated in the ICH group compared with the Sham group. Treatment with Efoba significantly reduced this proportion. The CTSC inhibitor AZD7986 also decreased the percentage of CitH3^+^ neutrophils, consistent with the effect of Efoba. Further immunofluorescence colocalization analysis showed that CTSC expression was increased in the brain tissue of ICH mice, whereas Efoba and the CTSC inhibitor AZD7986 significantly reduced CTSC distribution in the brain (Figure s10 E,F). Moreover, we observed significant colocalization of CTSC with neutrophil NETs in the ICH group, suggesting the involvement of CTSC in neutrophil activation and NETosis. In contrast, CTSC expression was reduced in the Efoba and AZD7986 treatment groups, accompanied by a marked decrease in NET formation (Figure 9I).

**FIGURE 9:**
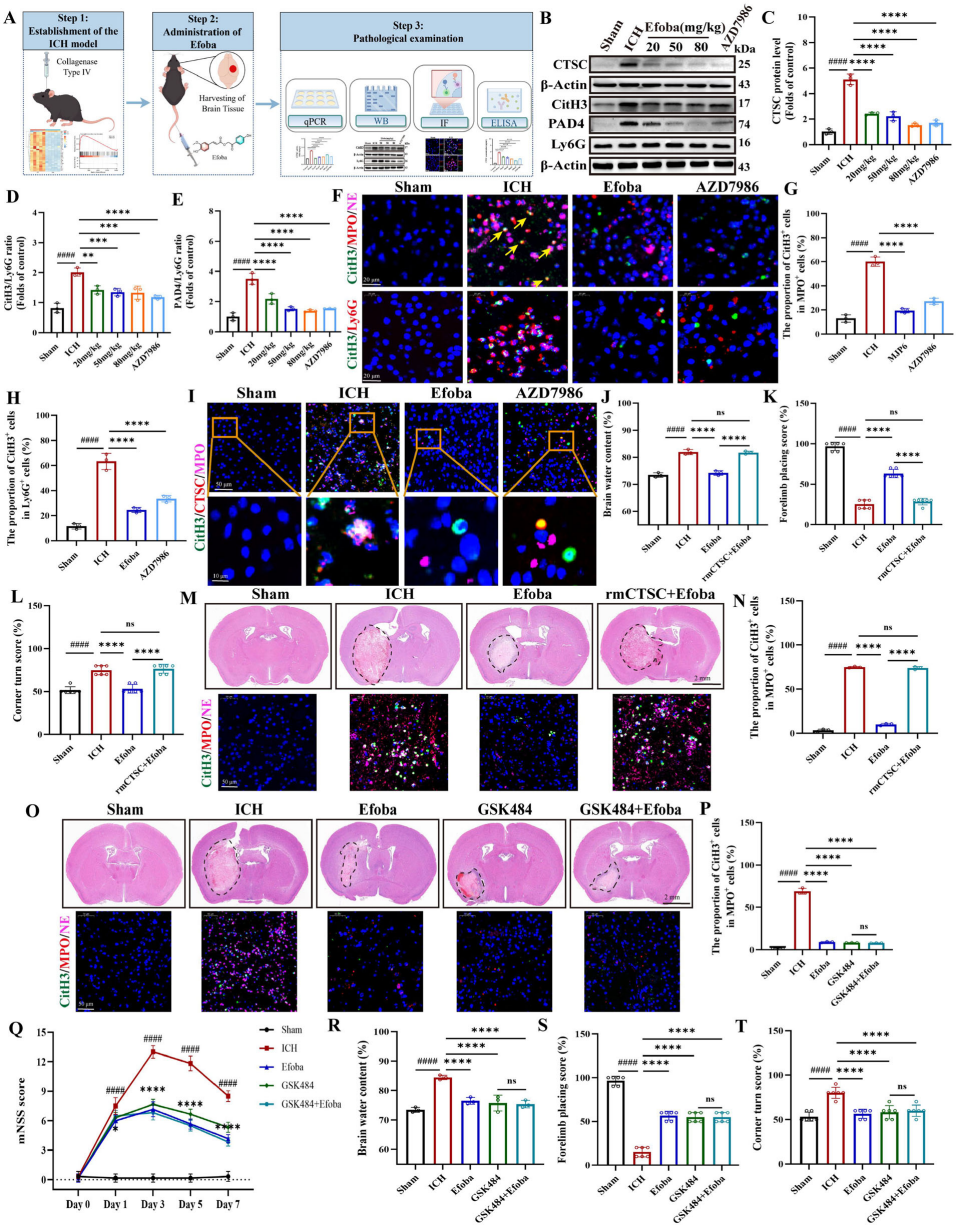
Efoba targets CTSC to suppress NET formation after ICH. (A) Workflow of animal experiments. (B–E) Western blot detection of CitH3, PAD4, Ly6G, and CTSC protein expression in brain tissues. (F,G) Representative images of NET formation stained for MPO (red), CitH3 (green), and NE (pink); the colocalization of Ly6G (red) and CitH3 (green). Nuclei were counterstained with DAPI (blue). Scale bars = 50 µm. (I) Co‐localization of CTSC (red) and NETs in brain tissues. Scale bars: 50 µm (overview) and 10 µm (magnified). (J) Brain water content after rmCTSC supplementation. (K) Forelimb placement test upon rmCTSC supplementation. (L) Corner turn test upon rmCTSC supplementation. (M,N) Representative H& E staining and representative images of NET formation stained for MPO (red), CitH3 (green), and NE (pink) after rmCTSC supplementation. Nuclei were counterstained with DAPI (blue). Scale bars = 50 µm. (O,P) Representative H&E staining and representative images of NET formation stained for MPO (red), CitH3 (green), and NE (pink) following NET inhibition by GSK484. Nuclei were counterstained with DAPI (blue). Scale bars = 50 µm. (Q) mNSS scores upon NET inhibition with GSK484 treatment. (R) Brain water content following NET inhibition by GSK484. (S) Forelimb placement test results in the GSK484‐mediated NET inhibition experiment. (T) Corner turn test following NET inhibition by GSK484. Data are shown as the mean values ± SD (n = 3). Statistical significance was determined by ANOVA. ^#^ ICH group compared with control group, ^*^ Efoba‐treated group compared with ICH group. ^*/#^ *p* < 0.05, ^**/##^ *p* < 0.01, ^***/###^ *p* < 0.001, ^****/####^ *p* < 0.0001, “ns” means no significant difference (*p* > 0.05).

We next performed recombinant CTSC (rmCTSC) rescue experiments in ICH mice; rmCTSC was administered to Efoba‐treated ICH mice to restore CTSC function. As demonstrated by brain water content measurement (Figure 9J), forelimb placing test (Figure 9K), corner turn test (Figure 9L), H&E staining (Figure 9M), MPO/CitH3/NE immunofluorescence (Figure 9M,N), and western blot analysis for CitH3 and PAD4 proteins (Figure S10G–I), rmCTSC supplementation largely abolished the neuroprotective phenotypes of Efoba, including improved sensorimotor function and alleviated brain tissue injury, as well as the down‐regulation of NET‐related CitH3 and PAD4.

To further demonstrate that the neuroprotective effect of Efoba depends on NETosis, we blocked the downstream NETosis pathway using the PAD4‐specific inhibitor GSK484. As shown by mNSS scores (Figure 9Q), H&E staining (Figure 9O), MPO/CitH3/NE immunofluorescence (Figure 9O,P), brain water content measurement (Figure 9R), forelimb placing test (Figure 9S), corner turn test (Figure 9T), and western blot analysis for CitH3 and PAD4 proteins (Figure S10J–L), GSK484 monotherapy markedly ameliorated ICH‐evoked brain injury. Nevertheless, combined administration of GSK484 and Efoba yielded no additional additive protective effects regarding neurological deficits, brain tissue damage, accumulation of NET‐like structures, cerebral edema, sensorimotor functional recovery, and the expression of NET‐associated proteins CitH3 and PAD4. Taken together, these results indicate that Efoba confers neuroprotection by inhibiting CTSC expression and function, which in turn diminishes NET formation following intracerebral hemorrhage.

## 3 Discussion

Intracerebral hemorrhage is characterized by high mortality and disability rates due to its complex pathological processes, and there remains a lack of targeted therapeutic strategies in clinical practice. Compelling evidence has demonstrated that microglial activation and neutrophil infiltration are central to secondary neuroinflammation in stroke and various other nervous system injuries [30, 31], the molecular mechanisms mediating the crosstalk between these two immune cell populations remain poorly understood.

Neuroinflammation serves as a central driver of secondary brain injury following ICH, with excessive microglial activation accompanied by robust neutrophil infiltration and NET formation representing key pathological cascades [32]. Accumulating clinical and preclinical data have detected abundant NETs within the hematoma, peri‐hematomal parenchyma, as well as plasma and drainage samples from ICH patients, highlighting NETs as critical mediators of ICH progression [15, 17]. Peri‐hematomal NETs are predominantly distributed around dense fibrin deposits, where they trigger a cascade of deleterious inflammatory responses, microvascular thrombosis, and neurotoxic reactions, thereby impairing hematoma clearance and neurological recovery, ultimately leading to poor prognosis [1]. During the acute phase of ICH, neutrophil levels persistently rise within injured brain tissues [33], and activated neutrophils release NETs, which subsequently induce fibrin deposition, enhance thrombus formation, and amplify coagulation activity [19, 34], exhibiting dual pro‐inflammatory and procoagulant properties [35, 36].

Consistent with these notions, our results demonstrate that ICH induces significant morphological transformation of microglia into an amoeboid pro‐inflammatory phenotype, accompanied by increased release of pro‐inflammatory cytokines and substantial neutrophil infiltration around the hematoma. RNA‐seq analysis further revealed enrichment of immune‐ and inflammation‐related pathways in injured brain tissue after ICH, underscoring the critical role of dysregulated neuroimmune responses in ICH progression. Notably, Efoba treatment significantly inhibited microglial activation, reduced neutrophil recruitment, and down‑regulated the expression of inflammatory cytokines both in vivo and in vitro, prompting us to further explore its downstream molecular target.

A central finding of this study is that activated microglia constitute the primary source of CTSC in the brain following ICH. We observed abundant accumulation of CTSC^+^TMEM119^+^ double‑positive cells in the peri‑hematomal region following ICH injury. CTSC exhibited only minimal colocalization with other cellular markers, including the neuronal marker NeuN, astrocytic marker GFAP, neutrophil marker MPO, endothelial marker CD31, and oligodendrocyte marker Olig2. This may be attributable to the intrinsically high lysosomal activity and elevated CTSC expression in microglia compared with other neural cell types [37]. These data confirm that the up‑regulated CTSC is predominantly derived from activated brain‑resident microglia, rather than infiltrating neutrophils, endothelial cells, or other cell populations. CTSC is a cysteine exopeptidase that is activated through enzymatic processing of its N‐terminal removable propeptide, pro‐CatC. Following multi‐site proteolytic cleavage, the mature CTSC monomer is released and consists of three tightly associated subunits: a 16 kDa exclusion domain, a 23 kDa catalytic heavy chain, and a 7.5 kDa light chain, which are held together by non‐covalent interactions [38]. CTSC is primarily involved in various cellular processes, including immune responses, inflammatory reactions, and tissue repair [39]. Under pathological conditions, CTSC expression is significantly up‐regulated and widely distributed within the central nervous system. Beyond physiological modulation of neuronal function in discrete brain regions, CTSC is strongly implicated in inflammation associated with diverse neurological disorders [40, 41]. However, its role in orchestrating neuroimmune interactions during ICH remains to be elucidated. This discovery positions microglia‐derived CTSC as a novel mediator in ICH pathology.

To rigorously validate CTSC as the direct functional target of Efoba, we employed a multi‐dimensional experimental approach to establish a robust evidence chain. Initially, Lip‐MS spectrometry screening identified CTSC as a high‐affinity binding partner of Efoba. Molecular docking, SPR, and MST assays further confirmed that Efoba directly binds to CTSC with high affinity. Mechanistically, Efoba alleviates neuroinflammation and neurological deficits by targeting CTSC, disrupting pathological microglia‐neutrophil communication and limiting NET formation. Efoba also restrained microglial activation and downstream pro‐inflammatory cytokine release. Cell‑specific knockout of CTSC in microglia markedly reduced NET generation under ICH‑mimetic conditions, confirming that CTSC ablation substantially diminishes the key NET signaling transmitted from activated microglia to neutrophils. Conversely, microglial CTSC overexpression alone was sufficient to robustly trigger NET formation even in the absence of strong inflammatory stimulation, further verifying the indispensable role of microglia‑derived CTSC in mediating microglia‑neutrophil pathological crosstalk. These loss‐of‐function and gain‑of‑function results provide solid and reciprocal evidence that CTSC acts as a core upstream regulator of NET generation during ICH progression. Collectively, these findings strongly support Efoba as a promising therapeutic candidate for ICH and offer novel mechanistic insights for the intervention of neuroinflammatory neurological diseases.

Our in vitro observations revealed that when purified neutrophils were directly stimulated with PMA, Efoba exhibited partial inhibitory effects on NET formation. This may be attributed to the abundant expression of CTSC in neutrophils [39]. However, direct stimulation of isolated neutrophils with OxyHb at the identical concentration used in the co‑culture system failed to induce evident NET morphology or CitH3‐positive signals, which may be attributed to the low sensitivity of neutrophils toward oxyhemoglobin [42]. Since the hemorrhagic microenvironment is primarily confined to the brain parenchyma, neutrophils are barely detectable within the brain under physiological conditions (Figure 7). Accumulating evidence has demonstrated that hemoglobin‐derived danger signals after intracerebral hemorrhage do not primarily exert their effects on neutrophils; instead, they preferentially activate microglia. Activated microglia subsequently secrete mediators to recruit and prime neutrophils, ultimately driving NET formation and subsequent brain injury [42, 43]. This provides a plausible mechanistic explanation for our in vitro finding that OxyHb monotherapy failed to initiate NETosis in isolated neutrophils, suggesting that microglia likely serve as an indispensable intermediate mediator required for OxyHb‐provoked NET production. In the hemorrhagic microenvironment following ICH, microglia become activated and release CTSC, which further recruits and activates circulating neutrophils to induce NET formation and subsequent neurological injury. Therefore, although Efoba can directly suppress NET production in neutrophils in vitro, it may predominantly exert its neuroprotective effects via regulating microglial function rather than direct neutrophil inhibition during intracerebral hemorrhage.

Previous studies have demonstrated that NETs disrupt blood‑brain barrier integrity via the ERK/MMP‑9 signaling pathway and boost microglial pro‑inflammatory activation through the cGAS‑STING or NLRP3 inflammasome axes [42, 44, 45]. Accordingly, peri‑hematomal brain tissue was selected as the primary region of interest for characterizing NET‑related secondary pathological alterations [46]. NET accumulation within this compartment triggers multiple detrimental injuries, including vasogenic edema, blood‑brain barrier disruption, demyelination, axonal atrophy, neuronal apoptosis, and microvascular thrombosis. Our multi‑dimensional experiments further confirmed that NET‑induced microthrombosis leads to edema and cerebral perfusion impairment, accompanied by tight‑junction disruption, myelin damage, and NET‑mediated neuronal apoptosis in the peri‑hematomal tissue. Collectively, multi‑layer pathological changes driven by peri‑hematomal NET accumulation account for the exacerbation of neurological deficits following intracerebral hemorrhage. Efoba is capable of concurrently ameliorating all aforementioned injuries by blocking the CTSC‑NET pathway.

Spatial proteomics reveals the features of in situ inflammatory remodeling in brain tissues after ICH. ICH triggers architectural disorganization in the peri‑hematomal region and elicits robust immune‑cell infiltration dominated by neutrophils, accompanied by markedly elevated abundance of neutrophils, CTSC‐positive activated microglia, and NET‐related markers within lesion sites [16, 45]. More importantly, the spatial distances among CTSC, the neutrophil marker Ly6G, and the core NET effector NE are significantly shortened following ICH, demonstrating tight in situ spatial coupling between microglia‐derived CTSC and neutrophil activation, which generates an aberrant pathological molecular interaction microenvironment [47]. Efoba intervention effectively reverses these pathological alterations. It not only reduces the abundance of diverse inflammatory and NET‐associated molecules as well as inflammatory cell infiltration in lesion areas, but also disrupts the pathological spatial coupling between CTSC and neutrophils and restores normal intermolecular spatial distances. Collectively, this spatial‑level evidence strongly confirms that Efoba targets the CTSC‑NET pathway to block pathological crosstalk between microglia and neutrophils, remodels the disordered cerebral inflammatory microenvironment after ICH, and ultimately alleviates secondary brain injury.

In terms of neurofunctional protection, a series of behavioral assessments including the mNSS, Morris water maze, open field test, rotarod test, and elevated plus maze demonstrated that Efoba significantly improved post‐ICH impairments in motor function, learning and memory, as well as anxiety and depression‐like behaviors. Additionally, Efoba ameliorated cerebral blood flow, reduced brain edema, preserved tight junction proteins, maintained blood‐brain barrier integrity, and promoted myelin regeneration and axonal repair. These findings indicate that Efoba not only suppresses local inflammation but also achieves comprehensive neuroprotection at both structural and functional levels, highlighting its potential for clinical translation. Compared with fresh garlic, which is limited in clinical application due to its strong pungency, poor tolerability, and limited palatability, aged garlic, which is produced through a mild fermentation process, offers more stable active components and reduced side effects [48]. In this study, we identified for the first time a high‐purity neuroprotective monomer, Efoba, from aged garlic, clarified its direct target as CTSC, elucidated its mechanism of action via the CTSC‐NET pathway, and demonstrated that Efoba can cross the blood‐brain barrier with low cytotoxicity. This work significantly addresses the major bottlenecks in natural product‐based neuroprotective research, including unclear active components, undefined targets, and ambiguous mechanisms [49]. It also provides a high‐quality lead compound for the development of CTSC‐targeted therapeutics for stroke.

Despite these significant findings, our study has inherent limitations. First, the in vivo efficacy and safety of Efoba were evaluated only in a mouse model of ICH, which cannot fully recapitulate the complexity of human ICH; particularly, the impact of age‐related comorbidities such as hypertension and diabetes on therapeutic efficacy remains to be characterized. Second, although CTSC has been identified as a key target, the possibility that Efoba may exert neuroprotective effects through modulation of other parallel pathways cannot be excluded. While our multi‐omics intersection strategy effectively narrowed the candidate pool and identified CTSC as the primary functional target of Efoba, we acknowledge that other top‐ranking LiP‐MS candidate proteins may also contribute to the polypharmacological effects of Efoba, reflecting the complex nature of natural product‐protein interactions. Future studies employing systematic approaches such as CRISPR‐Cas9‐based functional screening, thermal shift assays, or targeted pharmacological inhibition will be pursued to comprehensively map the full target landscape of Efoba and to evaluate the potential contribution of additional candidate proteins to its overall neuroprotective activity. Moreover, the structure‐activity relationship of Efoba and its druggability parameters, including metabolic stability and pharmacokinetic profile, warrant systematic investigation. Future research should focus on structural optimization of Efoba to enhance its potency and druggability, validation of its efficacy in large animal models, exploration of its therapeutic potential in other diseases driven by NETs or CTSC, and investigation of combination strategies to improve clinical outcomes.

In conclusion, this study identifies Efoba as a novel neuroprotective agent derived from aged garlic and delineates its innovative mechanism of action: Efoba exerts neuroprotective effects against ICH by targeting and inhibiting CTSC, thereby suppressing NET formation and mitigating aberrant inflammatory activation. These findings not only provide critical mechanistic insights into ICH intervention but also identify Efoba as a promising lead compound for the development of CTSC‐targeted neuroprotective therapeutics, while establishing a solid experimental foundation for advancing the medicinal application of aged garlic.

## 4 Conclusion

In summary, this study demonstrates that Efoba, a novel bioactive component from aged garlic, exerts neuroprotective effects against brain injury following ICH. Importantly, we established a scalable total synthesis route for Efoba, resolving the common bottleneck in natural product research of limited natural abundance and insufficient sample availability. Mechanistically, Efoba stabilizes microglial lysosomal membranes, inhibits CTSC release, and subsequently suppresses the formation of NETs, thereby effectively disrupting the CTSC‐NET pathway (Figure 10). These findings provide the first elucidation of the neuroprotective mechanism of Efoba and offer critical evidence supporting its therapeutic potential in alleviating secondary brain injury after ICH through CTSC inhibition, although its clinical application warrants further investigation. Notably, CTSC may not be the sole target of Efoba, as this compound may exert synergistic neuroprotective effects through modulation of other parallel pathways. Accordingly, the data presented in this study have been rigorously interpreted, and CTSC has not been overrepresented as the exclusive target of Efoba. This work not only opens new avenues for exploring the therapeutic potential of Efoba but also highlights the importance of elucidating its multi‐target mechanisms for expanding therapeutic applications in ICH.

**FIGURE 10:**
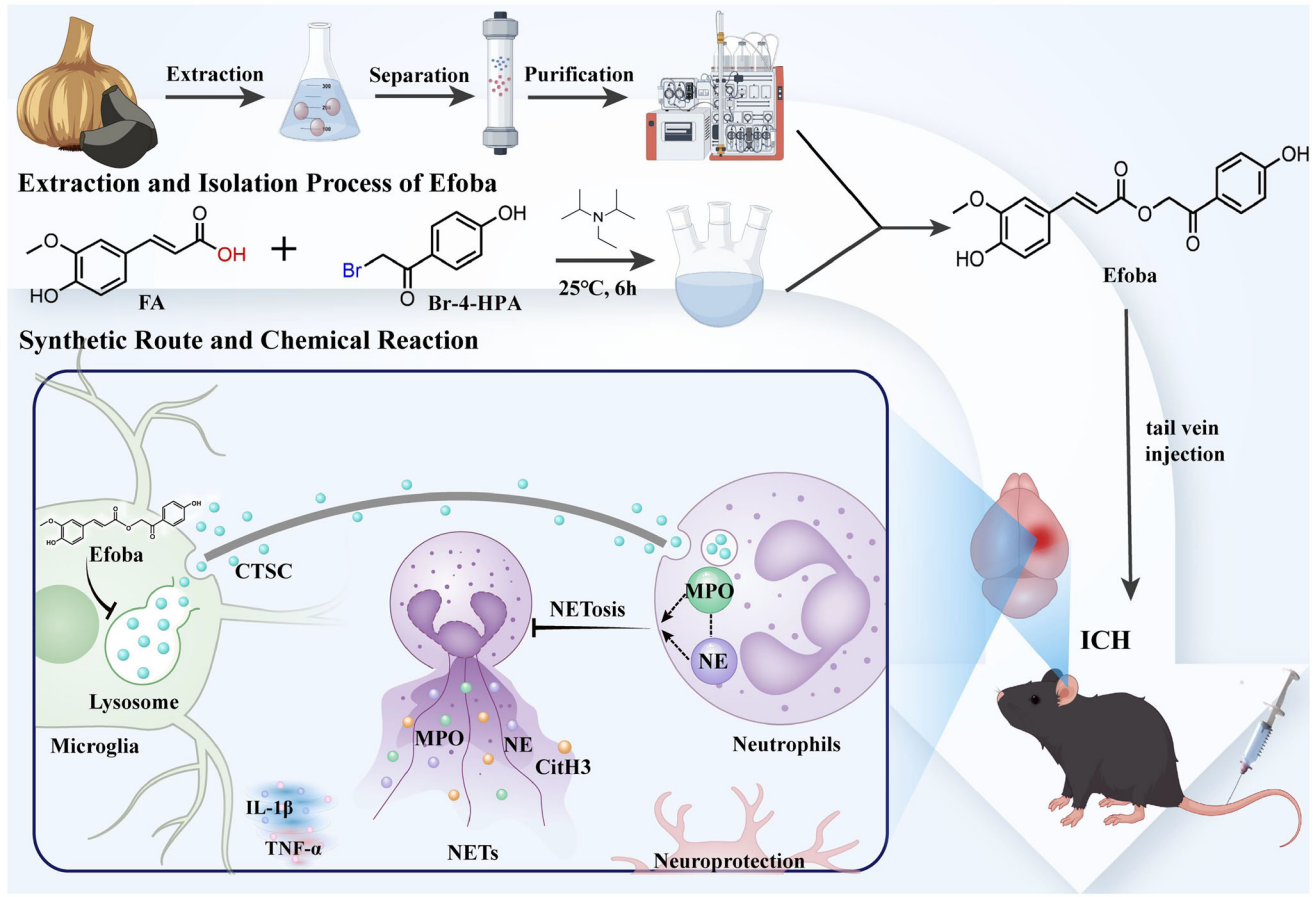
Schematic illustration of the neuroprotective mechanism of Efoba against ICH. Efoba directly targets CTSC, stabilizes microglial lysosomal membranes, and inhibits CTSC release, thereby suppressing NET formation and disrupting the CTSC‐NET pathway, which subsequently inhibits post‐ICH inflammatory responses and confers neuroprotection.

## 5 Experimental Section

### 5.1 Metabolomic Analysis of Garlic and Aged Garlic

Garlic and aged garlic cloves were peeled, ground, and approximately 50 mg of the powdered sample was placed in a cryogenic centrifuge tube. Metabolites were extracted by ultrasonic treatment with an appropriate volume of a 95% ethanol‐water mixture. Metabolite analysis was performed using liquid chromatography‐mass spectrometry (LC‐MS). Separation was achieved using a C18 reverse‐phase column with a gradient solvent system of methanol/water. Metabolite identification was conducted by comparing mass spectrometry data with standard spectra in public databases. Differential metabolites between garlic and aged garlic were assessed using multivariate statistical analysis. Enrichment analysis of metabolic pathways based on metabolite classification was performed to reveal the biological significance of the metabolic changes.

### 5.2 Isolation and Identification of the Novel Small Molecule Efoba From Aged Garlic

Approximately 500 g of ground aged garlic sample was placed in a beaker and extracted by ultrasonic treatment at room temperature for 2 h with an appropriate volume of 95% ethanol‐water mixture. This process was repeated three times, and the filtrates were combined. The combined extract was centrifuged (approx. 10 000 rpm, 10 min) to remove suspended solids. The supernatant was collected, and the solvent was removed using a rotary evaporator to obtain a concentrated crude extract. Liquid‐liquid extraction was performed using solvents of different polarities (e.g., ethyl acetate, n‐butanol) to separate fractions of varying polarity. Fractions were obtained using High‐Performance Liquid Chromatography (HPLC), identifying chromatographic peaks at their respective retention times. The fraction showing dense peaks between 6 and 10 min was further separated by silica gel column chromatography. The components obtained were purified first by preparative thin‐layer chromatography (TLC) and subsequently by semi‐preparative HPLC. The structure of Efoba was ultimately elucidated by combining Mass Spectrometry, 1D and 2D Nuclear Magnetic Resonance (NMR) spectroscopy, and Fourier‐Transform Infrared (FT‐IR) spectroscopy; the ^1^H‐NMR, ^13^C‐NMR, MS, and IR spectra are presented in Figure S1.

The NMR characterization data of Efoba are as follows: ^1^H NMR (600 MHz, DMSO, 25°C, TMS): *δ* = 3.82 (s, 3H, CH_3_), 5.45 (s, 2H, CH_2_), 6.59 (d, ^3^ *J*(H, H) = 15.9 Hz, 1H, CH), 6.80 (d, ^3^ *J*(H, H) = 8.16 Hz, 1H, CH), 6.83 (d, ^3^ *J*(H, H) = 8.58 Hz, 2H, CH×2), 7.15 (d, ^3^ *J*(H, H) = 8.16 Hz, 1H, CH), 7.36 (brs, 1H, CH), 7.60 (d, ^3^ *J*(H, H) = 15.9 Hz, 1H, CH), 7.83 (d, J = 8.58 Hz, 2H, CH×2); ^13^C NMR (150 MHz, DMSO, 25°C, TMS): *δ* = 55.6, 65.7, 111.2, 113.7, 115.4, 115.6, 115.6, 123.3, 124.7, 125.3, 130.2, 130.2, 145.7, 147.9, 149.6, 166.0, 163.7, 190.6.

### 5.3 Design and Validation of the Synthetic Pathway for Efoba

The synthetic route for this novel molecule was designed as follows: 4‐Hydroxy‐3‐methoxycinnamic acid (400 mg, 2.06 mmol), ω‐Bromo‐4‐hydroxyacetophenone (487 mg, 2.27 mmol), and MeCN (5 mL) were added sequentially to a dry, clean 100 mL three‐neck flask, followed by the addition of DIPEA (1.33 g, 10.30 mmol). The mixture was stirred at room temperature for 6 h. TLC (PE/EA = 4:1) indicated substantial reaction completion. Workup: Water (30 mL) was added, and the mixture was extracted with DCM (20 mL × 2). The combined organic phases were washed with saturated brine, dried over anhydrous Na_2_SO_4_, and concentrated to dryness. The crude product was purified by column chromatography (eluent ratio PE/EA = 4:1) to yield 600 mg of a white solid yield 90%.

### 5.4 Primary Microglia

Primary microglia were isolated according to the method of Du et al. [50]. Briefly, essential media were prepared: DMEM basal medium supplemented with 10% Fetal Bovine Serum (FBS) and 1% penicillin/streptomycin; specialized microglial culture medium supplemented with 20% LADMAC conditioned medium; and a digestion buffer containing papain and DNase. Culture flasks were coated with poly‐D‐lysine (PDL). Neonatal mice (postnatal days 1–4) were decapitated, meninges were removed, and the cerebellum and olfactory bulbs were excised. The remaining brain tissue was minced and digested in the digestion buffer for 20 min at 37°C under 5% CO_2_. Digestion was stopped by adding culture medium. After trituration, filtration, and centrifugation, a single‐cell suspension was obtained and plated onto the coated flasks. On day 2, the medium was replaced to remove cell debris and non‐microglial cells. When microglia reached 100% confluence around day 4, they were collected by gentle shaking of the flasks. The collected cells were plated in 6‐well plates. After adherence for 2 h, they were cultured in the specialized microglial medium. Suspended microglia could be collected repeatedly every 2–3 days thereafter.

### 5.5 Microglial Cell Culture

The murine microglial cell line BV2 was purchased from the Chinese Academy of Sciences Cell Bank and cultured in DMEM supplemented with 10% FBS, 100 U mL^−1^ penicillin, and 100 µg mL^−1^ streptomycin. Cell viability was assessed using the MTT assay. Cells were seeded in 96‐well plates at a density of 5 × 10^3^ cells/well, pretreated for 1 h with different concentrations of Efoba or 1% DMSO (vehicle control), followed by stimulation with 20 µм OxyHb for 24 h. Then, 20 µL of MTT solution (Beyotime Biotechnology, Shanghai) was added to each well and incubated at 37°C for 4 h. The formazan crystals formed were dissolved in 150 µL DMSO, and absorbance was measured at 570 nm using a BioTek microplate reader.

### 5.6 Neutrophil Isolation

Neutrophils were isolated from peripheral blood of 8‐week‐old male C57BL/6 mice using a commercial isolation kit (Mouse Peripheral Blood Neutrophil Isolation Kit, Solarbio, China). Briefly, under anesthesia, whole blood was collected via cardiac puncture into anticoagulant tubes. Blood samples were diluted 1:1 with an appropriate buffer and carefully layered over the density gradient medium provided in the kit. Centrifugation was performed at 1500 × g for 30 min at 25°C. The neutrophil layer at the interface was collected. Residual red blood cells were lysed using red blood cell lysis buffer. The isolated neutrophils were washed three times with phosphate‐buffered saline (PBS) and finally resuspended in Mouse Peripheral Blood Neutrophil Complete Medium (CP‐M150, Pricella, China) for subsequent use.

#### 5.6.1 Neutrophil Monoculture and NET‐Induction Assay

For neutrophil monoculture experiments (without microglia), purified neutrophils were seeded onto cell‑culture plates and divided into different treatment groups. Neutrophils were treated with PMA (100 nм) to trigger NET formation in the presence or absence of Efoba. In a parallel group, neutrophils were directly incubated with OxyHb (20 µм) at the identical concentration applied in the microglia‑neutrophil co‑culture system. After the corresponding stimulation, cells and culture supernatants were harvested for detection of NET‑related indicators, including SYTOX‑Green staining, immunofluorescence staining for Cit‑H3/MPO, extracellular dsDNA, and MPO‑DNA complex measurement.

#### 5.6.2 Compartment‑Specific Pre‑Incubation and Washout Co‑Culture Assay

Briefly, Microglia were pre‐incubated with Efoba, stimulated with OxyHb. After thorough washout to remove residual Efoba, the pre‐treated microglia were co‐cultured with untreated neutrophils. Neutrophils were pre‐incubated with Efoba, followed by complete washout of the compound. These neutrophils were then co‐cultured with OxyHb‐activated microglia. Microglia were pre‐incubated with Efoba, stimulated with OxyHb and washed to eliminate residual Efoba. Separately, neutrophils were also pre‐incubated with Efoba and washed. The two cell populations were then co‐cultured.

#### 5.6.3 Conditioned Medium (CM) Assay

Microglia were seeded in 6‑well plates and divided into control group, OxyHb‑stimulated group, and Efoba‑pretreated plus OxyHb‑stimulated group. Microglia were pre‑incubated with or without Efoba for 2 h, followed by OxyHb stimulation for 24 h. Cell culture supernatants were harvested, centrifuged at 3000 × g for 10 min at 4°C to remove cell debris, and filtered through 0.22 µm membranes to obtain conditioned medium. The supernatant collected from OxyHb‑treated microglia was designated CM‑OxyHb, whereas supernatant collected from Efoba‑pretreated and OxyHb‑challenged microglia was designated CM‑Efoba. For NET‑induction assays, freshly isolated primary neutrophils were plated and incubated with different batches of conditioned medium. For the rescue group, recombinant mouse rmCTSC protein was supplemented into CM‑Efoba at the predetermined working concentration. After incubation, cell supernatants and cellular samples were collected for detection of extracellular dsDNA, MPO‑DNA complexes, as well as SYTOX‑Green staining and CitH3‑MPO immunofluorescence co‑staining.

### 5.7 Transwell Co‐Culture of Microglia and Neutrophils With Lentiviral Overexpression of CTSC

Primary microglia or BV2 cells were cultured in Dulbecco's Modified Eagle Medium (DMEM) supplemented with 10% FBS and 1% penicillin‐streptomycin at 37°C under 5% CO_2_. In the Transwell co‐culture system, microglia (primary or BV2 cells), pretreated or untreated with 20 µм OxyHb, were seeded in the upper chamber, while freshly isolated mouse peripheral blood neutrophils were placed in the lower chamber.

The homozygous CTSC knockout BV2 cell line was generated via CRISPR‑Cas9 ribonucleoprotein (RNP) mediated fragment deletion editing targeting exon 2 of mouse CTSC (NCBI Gene ID:13032, transcript NM_009982). Briefly, Cas9‑sgRNA RNPs targeting CTSC exon 2 were electroporated into BV2 cells. Single‑cell clones were isolated, expanded, and genotyped by PCR amplification of the edited genomic region followed by Sanger sequencing to confirm biallelic deletion. Loss of CTSC protein expression was further validated by western blot. Wild‑type BV2 cells served as the isogenic control. The established CTSC knockout BV‑2 cells and control cells were subjected to subsequent Transwell co‑culture and conditioned medium NETs induction assays.

To investigate the function of Cathepsin C (CTSC), microglia were transduced prior to co‐culture using a lentiviral vector encoding the full‐length mouse CTSC (Lv‐m‐Ctsc‐3xflag‐ZsGreen‐PURO, titer 4 × 10^8^ TU mL^−1^) or an empty vector control (HBLV‐ZsGreen‐PURO, titer 3 × 10^8^ TU mL^−1^). The virus was constructed based on the pHBLV‐CMV‐MCS‐3FLAG‐EF1‐ZsGreen‐T2A‐PURO vector and packaged in 293T cells using a three‐plasmid system (including psPAX2 and pMD2G), followed by purification via ultracentrifugation. After 48 h of transduction, cells were treated with OxyHb and then subjected to co‐culture with neutrophils.

### 5.8 Animals and Treatments

The animal experiments were approved by the Ethics Committee of Jining No. 1 People's Hospital (protocol number: JNRM2025DW054). All animal experiments were approved by Shandong Pengyue Experimental Animal Technology Co., Ltd. (License No. SCXK (Lu) 2022‐0006), fully complying with national laboratory animal welfare regulations and ARRIVE 2.0 guidelines. Specific pathogen‐free (SPF) healthy male C57BL/6J mice (8 weeks old, 20 g ± 3 g) were housed in SPF‐grade animal facilities. The standardized housing environment included an ambient temperature of 20°C–25°C, humidity 45%–65%, a 12 h light/dark cycle, and free access to food and water. All mice were randomly divided into sham group, ICH group, Efoba‐L group (20 mg kg^−1^), Efoba‐M group (50 mg kg^−1^), Efoba‐H group (80 mg kg^−1^), AZD7986 group (1 mg kg^−1^), GSK484 group (4 mg kg^−1^), GSK484+Efoba group, and rmCTSC+Efoba group before ICH modeling. Investigators conducting neurological function scoring, brain water content measurement, and histological evaluation were blinded to the group allocation of animals. Sample size was determined based on our preliminary experiments and previously published ICH studies. For ICH surgery, mice were anesthetized via intraperitoneal injection of pentobarbital sodium (25 mg kg^−1^). Mice were excluded from analysis if severe hemorrhage, death, or obvious surgical failure occurred during or after the operation.

To verify that inhibition of NET formation is an essential mechanism underlying the neuroprotective effect of Efoba, experiments were conducted using GSK484 to inhibit NETs. We set up five groups, Sham group (n = 12), ICH group (n = 9), ICH+Efoba group (50 mg kg^−1^, n = 9), ICH+GSK484 (n = 9), and ICH+GSK484+Efoba group (n = 9). ICH+GSK484 and ICH+GSK484+Efoba group received 4 mg kg^−1^ (dissolved in sterile saline) of GSK484 (GLPBIO, USA), a PAD4 inhibitor, via tail vein injection [51].

We established the following experimental groups for recombinant CTSC rescue treatment: Sham group (n = 9), ICH group (n = 9), ICH+Efoba group (50 mg kg^−1^, n = 9), and ICH+rmCTSC+Efoba group (50 mg kg^−1^, n = 9). Recombinant mouse CTSC (rmCTSC) was dissolved in sterile PBS at a final concentration of 0.1 µg µL^−1^. For rescue treatment, 1 µg rmCTSC (10 µL volume) was stereotactically injected into the peri‐hematoma area of ICH mice via tail vein injection [47, 52].

Efoba was dissolved in a vehicle consisting of 2% dimethyl sulfoxide (DMSO), 40% polyethylene glycol 300 (PEG300), 5% Tween‐80, and 53% normal saline to reach the target working concentrations required for subsequent experiments. Treatments were administered once daily for 3–7 consecutive days. The in vivo dose gradients adopted in this study were determined based on published research on garlic bioactive monomers and our team's previous findings. Allicin and alliin are generally used at an effective dose of 50 mg kg^−1^ in mouse models of intracerebral hemorrhage and neurological disorders. Our earlier research confirmed that 50 mg kg^−1^ alliin exerts neuroprotective effects against intracerebral hemorrhage by inhibiting ferroptosis with favorable safety [5, 53]. Based on published literature and our prior work on garlic derived bioactive molecules, Efoba exhibits physicochemical properties similar to alliin, a compound reported to confer neuroprotection and display favorable safety profiles in intracerebral hemorrhage models. Among tested in vivo dosage groups, the 50 mg kg^−^^1^ dose produced prominent therapeutic efficacy comparable to the 80 mg kg^−^^1^ group, together with improved mouse survival. Accordingly, the 50 mg kg^−^^1^ dose was selected for all subsequent animal experiments to investigate its underlying mechanism of action. As a novel small molecule derived from fermented aged garlic, Efoba possesses physicochemical and toxicokinetic properties similar to alliin. Hence, 50 mg kg^−1^ was selected as the core medium therapeutic dose, while a low dose (20 mg kg^−1^) and a high safe dose (80 mg kg^−1^) were also set to observe dose‐effect trends. Acute and subchronic toxicity tests revealed that Efoba caused no visceral toxicity at doses below 480 mg kg^−1^. In vitro MTT assays verified that Efoba exhibited no cytotoxicity against microglia at concentrations ≤ 100 µм, so gradients of 20, 50, 80 and 100 µм were used for cellular functional analyses. For all in vivo animal experiments, Sham and ICH groups were administered an equal volume of this vehicle as the blank vehicle control. For cell experiments, Efoba stock solution was prepared by dissolving Efoba in DMSO, and further diluted with cell culture medium immediately prior to use. The final DMSO concentration in all culture groups was maintained at ≤ 0.1%, and untreated blank cells as well as OxyHb‐stimulated model cells were cultured in medium containing the same concentration of DMSO as solvent controls.

### 5.9 In Vivo Toxicity Evaluation of Efoba

Healthy C57BL/6 mice were randomly assigned to the control group (n = 15) and Efoba‐treated groups. Efoba (40, 80, 240, 480, and 960 mg kg^−1^, n = 15 per group) or vehicle was administered intraperitoneally daily for 5 weeks. Body weight was recorded every 5 weeks. On day 15, 40, 480, and 960 mg kg^−1^ groups were euthanized, and major organs were collected for H&E staining to evaluate histopathological changes. Brain tissue was collected to quantify Efoba concentration using LC‐MS/MS.

#### 5.9.1 Hematological Analysis

Blood samples were collected from anaesthetized mice (control, 40, 480, 960 mg kg^−^^1^ Efoba groups, intragastric administration once daily for 14 consecutive days, n = 6 per group) via retroorbital venous plexus into EDTA coated anticoagulant tubes. Complete blood count parameters, including white blood cells (WBC), red blood cells (RBC), hemoglobin (HGB), hematocrit (HCT), platelet (PLT), lymphocyte percentage, neutrophil percentage, and other routine hematological indicators were immediately measured using an automated hematology analyzer according to the manufacturer's instructions.

#### 5.9.2 Serum Biochemistry for Liver and Kidney Function

Following 14 days repeated intragastric Efoba administration (control, 40, 480, 960 mg kg^−^^1^; n = 6 per group), whole blood was collected and allowed to stand at room temperature for 30 min, followed by centrifugation at 3000 g for 15 min at 4°C to obtain serum. Serum biochemical indicators were quantified by an automatic biochemical analyzer. Liver related markers included alanine aminotransferase, aspartate aminotransferase (AST), alkaline phosphatase (ALP). Kidney‐related markers included creatinine (Cr), blood urea nitrogen (BUN), and uric acid (UA).

#### 5.9.3 Coagulation Function Assay

For repeated dose normal mice: After 14 days Efoba intragastric treatment (control, 40, 480, 960 mg kg^−^^1^, n = 6 per group), whole blood was harvested into 3.2 % sodium citrate anticoagulant tubes (blood‑citrate volume ratio 9:1). Plasma was obtained by centrifugation at 3000 g for 15 min at 4 °C. Coagulation parameters including prothrombin time, activated partial thromboplastin time (APTT), thrombin time (TT) and fibrinogen (FIB) were determined using an automatic coagulation analyzer. For ICH model mice, the mice were divided into Sham, ICH, ICH+Efoba groups (n = 6 per group). Brain tissue and blood samples were collected within 6 h after ICH induction. Plasma was prepared as described above, and PT, APTT, TT, and FIB were measured to evaluate early stage coagulation status after intracerebral hemorrhage.

#### 5.9.4 Quantification of Initial Hematoma Volume

Mice were randomly assigned to Sham, ICH, and ICH+Efoba groups (n = 7 per group). At 6 h after ICH surgery, mice were perfused with pre‐cooled phosphate‐buffered saline, and whole brain tissues were harvested. Serial coronal brain sections were cut at 200 µm intervals throughout the hematoma‐containing brain region. Sections were stained with hematoxylin and eosin. Hematoma area on each slice was traced and calculated using ImageJ software. Hematoma volume was calculated by summing the area of hematoma across all sections multiplied by section interval thickness. The investigators performing volume quantification were blinded to experimental group assignments.

At indicated time points after ICH modeling, whole blood samples were collected from mice in the control group and Efoba groups (40, 480, 960 mg kg^−^^1^) for hematological analysis. Serum was separated to detect liver and renal function indices, and plasma was harvested for coagulation parameter detection and measurement of circulating TNF‐α and IL‐6.

### 5.10 ICH Model Establishment

Male SPF‐grade C57BL/6J mice, approximately 8 weeks old, were randomly assigned to groups (Sham, ICH, Efoba treatment) using a random number table. The ICH model was induced by intrastriatal injection of collagenase. Mice were fasted for 8 h before surgery and anesthetized with sodium pentobarbital (25 mg kg^−1^). The mouse's head was fixed in a stereotaxic apparatus in a prone position. After disinfection and skin preparation, a midline incision was made on the dorsal scalp, subcutaneous tissue was separated, and the bregma was exposed. Using the bregma as the origin (coordinates: anterior 0.3 mm, lateral right 2.3 mm), a small burr hole was drilled. A solution of Type IV collagenase (C2399, Sigma; 0.2 U µL^−1^ in saline; volume 0.2 µL) was drawn into a microsyringe. The needle was inserted vertically to a depth of 3.8 mm slowly. Upon reaching the target site, the collagenase was injected at a rate of 0.4 µL min^−1^. The syringe was left in place for 10 min post‐injection to prevent backflow, then slowly withdrawn. The burr hole was sealed with bone wax, local pressure was applied for hemostasis, and the scalp was sutured. Mice were transferred to a 37°C heating pad until fully conscious. Post‐surgery, mice had free access to water and solid food under constant light conditions. Efoba at corresponding concentrations was administered via tail vein injection within 0.5 h after collagenase injection. Control and model group mice received an equal volume of saline.

### 5.11 Lip‐MS Spectrometry

Target identification using Lip‐MS spectrometry. Based on the limited proteolysis‐based small molecule interaction mapping technology, the principle is that when a small molecule binds to a protein, it induces changes in the local structure of the protein. This structural alteration affects the accessibility of protease to specific cleavage sites, thereby changing the peptide map generated after cleavage. By comparing the peptide abundance with and without the small molecule presence via mass spectrometry, these conformotypic peptides can be identified, enabling the unbiased discovery of small molecule‐protein interactions [54]. The cell lysate was aliquoted into samples, each containing 100 µg of proteome, which were incubated with Efoba (final concentration: 33 nmol) at 25°C for 10 min. Proteinase K from Tritirachium album (Sigma–Aldrich) was added simultaneously to all proteome‐Efoba samples at an enzyme‐to‐substrate mass ratio of 1:100, followed by incubation at 25°C for 5 min. Digestion was terminated by heating the samples at 96°C for 5 min, followed by addition of deoxycholate (Sigma–Aldrich) to a final concentration of 2%. Protein fragments from the Proteinase K limited hydrolysis step were reduced with 10 mм DTT at 37°C for 30 min, then alkylated with 40 mм iodoacetamide in the dark at 25°C for 45 min. Samples were digested with trypsin (enzyme‐to‐substrate ratio 1:50) overnight at 37°C with shaking. Deoxycholate was precipitated by adding trifluoroacetic acid (TFA) to a final concentration of 2%. The peptide mixture pH was adjusted to below 3 using formic acid. Desalting was performed using C18 columns (CNW). Finally, samples were dried in a vacuum centrifuge, reconstituted in 0.1% formic acid, and immediately analyzed by mass spectrometry. Peptide separation was performed using a nano‐UPLC system (nanoElute2) coupled to a timsTOF Pro2 mass spectrometer equipped with a nano‐electrospray ion source. Raw data files were processed using SpectroMine software (version 4.2.230428.52329; Biognosys AG) with the Pulsar search engine for database searching, followed by qualitative analysis. The Lip‐MS data are presented in Table S6.

### 5.12 High‐Throughput Virtual Screening (HTVS)

The molecular structure and SMILES of Efoba were uploaded to the WeMol intelligent drug target prediction platform (https://wemol.wecomput.com/ui/#/). Based on the small molecule topological properties, pharmacophore features, and ligand‐protein interaction patterns, the platform performed systematic target fishing and prediction using built‐in machine learning and reverse docking algorithms. A series of potential target proteins were generated and output. Candidate targets were further filtered and prioritized according to multiple quantitative indicators, including binding affinity prediction scores, drug‐target relevance, ligand compatibility, and literature‐supported biological plausibility. Finally, the most reliable target with the highest comprehensive ranking was selected for subsequent mechanistic validation. The results of high‐throughput virtual screening are shown in Table S7.

### 5.13 RNA Sequencing and Bioinformatics Analyses

At 7 days post‐ICH, mice were anesthetized with pentobarbital, perfused transcardially with PBS, and brain tissues were rapidly frozen in liquid nitrogen. Total RNA was extracted using TRIzol reagent according to the manufacturer's instructions. RNA purity and concentration were determined using a NanoDrop 2000 spectrophotometer, and RNA integrity was assessed using an Agilent 2100 Bioanalyzer. Library sequencing was performed on the Illumina NovaSeq 6000 platform. Differential expression gene analysis was performed using DESeq2 software, with genes meeting the thresholds of q‐value < 0.05 and |fold change| > 2 defined as DEGs. We thank OE Biotech, Inc. (Shanghai, China) for assisting in sequencing and/or bioinformatics analysis.

### 5.14 LC‐MS/MS‐Based Proteomic Analysis

Brain tissues were ground in liquid nitrogen and centrifuged to obtain clarified lysates. Protein concentration was determined using the BCA/BSA method. Enzymatic digestion proceeded as follows: proteins were reduced with 5 mм dithiothreitol (DTT), alkylated with 15 mм iodoacetamide (IAA), and then digested overnight with trypsin using a precipitation or filter‐aided sample preparation (FASP) method. The resulting peptides were acidified, desalted, and quantified. Peptides were separated by nanoflow liquid chromatography (Vanquish Neo system, Thermo Fisher Scientific) and analyzed using an Astral mass spectrometer (Thermo Fisher Scientific) in data‐independent acquisition mode over a scan range of 380–980. DIA data were processed using DIA‐NN software, searching against a protein database with trypsin specificity, carbamidomethylation as a fixed modification, oxidation/acetylation as variable modifications, and applying a 1% false discovery rate (FDR) filter at the protein level.

### 5.15 Spatial Proteomics

Formalin‐fixed, paraffin‐embedded (FFPE) tissue sections (5 µm thick) were mounted onto anti‐offset slides, ensuring the tissue was centered within an 18 mm × 35 mm area, and incubated at 60°C overnight to melt the paraffin thoroughly. Sections were deparaffinized in xylene and rehydrated through a graded ethanol series. Antigen retrieval was performed using 10× Tris‐EDTA buffer (pH = 9, Solarbio, C1038) in a pressure cooker. Sections were then incubated in Staining Buffer (Akoya Biosciences, 240198) at room temperature for 20 min. Antibodies were prepared on ice. Staining involved room temperature blocking and antibody incubation, followed by sequential fixation with 1.6% PFA (CITOTEST, 80011–0063), ice‐cold methanol (Sigma, 34860–1L‐R), and Fixative Reagent (Akoya Biosciences). Nuclei were stained using DAPI according to the PCF standard. Tissue regions were identified based on brightfield images, aiming to capture all areas using the PCF's tissue recognition sensitivity for final fluorescent quantitative image acquisition.

For the PCF staining cycle, each round involved staining tissue samples with DAPI and up to three additional antibodies, imaging the labeled biomarkers, then dissociating and washing off the fluorescent chromophores from that round using a dissociation buffer. This cycle was repeated until all proteins in the panel were imaged. Fully stitched and precisely aligned images were generated through automated image acquisition and processing. Truly single‐cell, highly multiplexed protein immunofluorescence analysis was performed using the HALO digital pathology image analysis platform. Specific Regions of Interest were defined to extract target information, determine the abundance and localization of specific proteins, enabling qualitative and quantitative analysis of spatial contexts [55].

### 5.16 Molecular Dynamics Simulation

Molecular dynamics simulations were performed on the WeMol platform (https://wemol.wecomput.com/ui/#/). The simulation system utilized the ff19SB force field and the OPC water model, with the complex solvated in a cubic water box. The integration time step was set to 2 fs. The system was maintained at 300 K and 1 bar using the V‐rescale thermostat and Parrinello‐Rahman barostat, respectively. The system underwent energy minimization, followed by sequential equilibration (200 ps NVE ensemble and 100 ps NPT ensemble), before a final 100 ns production simulation. Trajectory analysis, including Root Mean Square Deviation (RMSD) and Solvent Accessible Surface Area (SASA) calculations, was performed using built‐in modules in AmberTools.

### 5.17 Molecular Docking

Molecular docking analysis was used to study the binding affinity and interactions between the active compound and its potential target. The 3D structure of Efoba was energy‐minimized using ChemDraw 3D software, and the resulting conformation was used for docking simulations. The 3D structure of Cathepsin C (CTSC) was obtained from the Protein Data Bank (PDB ID: 4cdd). The protein structure was prepared using AutoDock Tools software, including removal of water molecules and addition of hydrogen atoms. A grid box was set in blind docking mode to identify potential binding sites accurately. Docking was performed using AutoDock Vina 1.2.2 software. Binding interactions were evaluated based on binding energy, and receptor‐ligand interactions were visualized using Discovery Studio software.

### 5.18 Microscale Thermophoresis (MST)

The Escherichia coli codon table was used for codon optimization. Pro‐CTSC protein was expressed and purified. Pro‐CTSC (200 nм) was incubated with varying concentrations of Efoba in ligand buffer (100% DMSO) at room temperature for 5 min. Samples were then loaded into hydrophilic capillaries (NanoTemper Technologies, Germany) and analyzed using an MST‐NT.115 instrument. Binding data were analyzed using thermophoresis or temperature‐related intensity change analysis as previously described. Data were normalized to ΔFnorm [%] (10^*^(Fnorm(bound)—Fnorm(unbound))) or binding fraction (ΔFnorm [%] / amplitude). Dissociation constants were calculated by nonlinear regression analysis.

### 5.19 Surface Plasmon Resonance Analysis

SPR experiments were performed on a pre‐treated CM5 chip. The chip surface was activated by injecting a mixture of 400 mм EDC and 100 mм NHS at a flow rate of 10 µL min^−1^ for 420 s. Subsequently, CTSC protein diluted to 50 µg mL^−1^ in immobilization buffer was injected over the sample flow cell (Fc4) at 10 µL min^−1^, achieving an immobilization level of approximately 13 100 Response Units (RU). The reference flow cell (Fc3) was left unmodified. Remaining reactive groups were blocked by injecting 1 м ethanolamine‐HCl (10 µL min^−1^, 420 s). Analyte Efoba was diluted in running buffer to seven concentrations (50–0 µм) and injected using a multi‐cycle kinetics mode at a flow rate of 30 µL min^−1^, with association and dissociation times set to 120 and 300 s, respectively. Chip regeneration relied on natural dissociation between cycles.

### 5.20 Acridine Orange (AO) Staining

Acridine Orange (AO) is a metachromatic fluorophore that permeates cell and lysosomal membranes. When the lysosomal membrane is intact, AO within lysosomes emits orange‐red fluorescence upon excitation at 488 nm. Conversely, if the lysosomal membrane is compromised (Lysosomal Membrane Permeabilization, LMP), AO in the cytoplasm exhibits green fluorescence. Additionally, a Calcein‐AM/PI double‐staining kit (Yeasen, China) was used to assess BV2 cell membrane permeability. Briefly, BV2 cells seeded in 6‐well plates were washed with PBS and resuspended in 1 mL of AO working solution (5 µg mL^−1^, Sigma, USA) or 1 mL of Calcein‐AM working solution (containing 1 µм Propidium Iodide and 1 µм Calcein‐AM), followed by incubation at 37°C for 30 min. Fluorescence intensity was observed, and images were captured using an Olympus microscope.

### 5.21 Neutrophil Extracellular Traps Detection

NET formation was analyzed by quantifying double‐stranded DNA (dsDNA) in cell supernatants using the PicoGreen dsDNA Quantification Kit (Solarbio, China) and Citrullinated Histone H3 (CitH3) using an ELISA kit (Cayman Chemical, USA). All procedures were strictly followed according to the manufacturers’ instructions.

### 5.22 Detection of Efoba Penetration Across the Blood‐Brain Barrier Into Brain Tissue

Eight mice were evenly divided into two groups: normal mice and ICH model mice. After successful ICH model induction, Efoba was administered via tail vein injection at a dose of 50 mg kg^−1^ for three consecutive days. On day 4, brain tissues were collected from both groups. LC‐MS analysis was performed on the brain tissues, using pure Efoba as a control, to analyze the distribution of Efoba within the brain.

### 5.23 Brain Water Content Measurement

Brain water content was calculated using the wet‐dry weight method. Mice were sacrificed 3 days post‐ICH, and brain tissues were harvested. The wet weight was immediately measured. Tissues were then dried in an oven at 100°C for 2 days to determine the dry weight. Brain water content was calculated as: [(Wet Weight—Dry Weight) / Wet Weight] × 100%.

### 5.24 Brain Sectioning

Mice treated with different concentrations of Efoba were used to observe the inhibitory effect of Efoba on hematoma formation. Three days post‐ICH model induction, animals were anesthetized and sacrificed, and whole brains were rapidly removed and frozen in liquid nitrogen. The frozen brain was mounted on a specimen disc in a cryostat pre‐cooled to approximately −20°C. Coronal sections were cut at a set thickness. Sections were thaw‐mounted onto pre‐chilled, coated glass slides. Sections were allowed to adhere briefly, then stored at −80°C. For each brain, five sequential coronal sections from anterior to posterior were prepared for photography.

### 5.25 H&E Staining

For H&E staining of brain tissue, deeply anesthetized mice were perfused transcardially, and brains were removed carefully and fixed by immersion in ice‐cold 4% paraformaldehyde for 6 h, followed by post‐fixation of smaller tissue blocks for approximately 18 h. After fixation, tissues were rinsed under running water for 1 day, dehydrated through a graded ethanol series, cleared in xylene, infiltrated with molten paraffin, and embedded. Sections (5 µm thick) were cut using a microtome, mounted on slides, and dried. Sections were deparaffinized, rehydrated, stained with Harris hematoxylin for 10 min, rinsed, differentiated in acid alcohol, blued, counterstained with eosin for 5 min, dehydrated, cleared, and mounted with neutral balsam for microscopic examination.

### 5.26 TUNEL Staining

Deeply anesthetized mice were perfused, and brains were removed and fixed in 4% paraformaldehyde for 24–48 h at 4°C. Sections were permeabilized with 0.1% Triton X‐100 in PBS for 10 min at room temperature. The TUNEL reaction mixture (containing TdT enzyme and biotin‐labeled dUTP in buffer) was prepared according to the kit instructions and applied to the sections, which were incubated in a humidified chamber at 37°C for 60 min in the dark. After washing, sections for brightfield detection were incubated with streptavidin‐HRP conjugate for 30 min at room temperature, washed again, and developed with DAB substrate until brown precipitate formed (5–10 min), monitored under a microscope. Finally, sections were counterstained, dehydrated, cleared, and mounted for observation and quantification of apoptotic cells under a light microscope.

### 5.27 Cerebral Blood Flow

Cerebral cortical blood flow was monitored for 2 h, 1, 3, 5, and 7 days post‐ICH using a non‐invasive laser speckle imager with a 70 mW built‐in laser and a 1388 × 1038‐pixel CCD camera (PeriCam PSI System, Perimed, Sweden) as recently described. After anesthetizing mice with sodium barbital‐free anesthesia, place them on a stereotaxic instrument and expose the skull. The CCD camera was installed 10 cm above the skull to capture the CBF images in a scanning area of 2.0 × 2.0 cm. The ipsilateral cortical blood flow (perfusion unit, PU) was calculated and presented as mean perfusion values using the vendor‐supplied PIMSoft software.

### 5.28 T2‐Weighted Magnetic Resonance Imaging

Mice were anesthetized with sodium pentobarbital, and T2^*^‐weighted gradient echo sequence images were acquired using an MRI scanner at 1, 3, and 7 days post‐ICH. Twenty coronal slices (1.0 mm thick) were obtained from the frontal pole to the brainstem. The T2^*^ lesion volume, representing the hematoma, was delineated along the hypointense (dark) signal boundary, including the central isointense or hyperintense areas. The total T2^*^ lesion volume was calculated by multiplying the slice thickness by the sum of the lesion areas across all slices.

### 5.29 Transmission Electron Microscopy

At 72 h post‐ICH, mice were anesthetized and perfused transcardially with 0.1 mol L^−1^ Sorensen's buffer (pH 7.4) containing 4% paraformaldehyde and 2.5% glutaraldehyde. Brain tissues were dissected and trimmed into small pieces (1 mm^3^). Samples were post‐fixed in 1% osmium tetroxide, dehydrated through a graded ethanol series, infiltrated with propylene oxide, embedded in Epon resin, and sectioned. Stained sections were observed under a transmission electron microscope (7800, Hitachi), and digital images were acquired using a Hamamatsu ORCA‐HR camera.

### 5.30 Scanning Electron Microscopy

At 72 h post‐ICH, animals were deeply anesthetized and sacrificed, and brain tissues were rapidly dissected. Small tissue blocks (1 mm^3^) were fixed in 2.5% glutaraldehyde, post‐fixed in 1% osmium tetroxide for 2 h, and dehydrated through a graded ethanol series (30%, 50%, 70%, 80%, 90%, 100%). After critical point drying and gold sputtering, samples were imaged using a scanning electron microscope (Sigma360, Zeiss).

### 5.31 Modified Neurological Severity Score (mNSS)

The mNSS is a composite score (max 18 points) assessing motor, sensory, balance, and reflex functions, with higher scores indicating more severe neurological impairment. Tests included: raising the tail (assessing forelimb/hindlimb flexion and head movement, max 3 points), floor walking (from normal walking to falling toward the paretic side, max 3 points), sensory tests (visual/tactical placing test, proprioceptive test, 1 point each), beam balance test (performance on a 1.5 cm‐wide beam, scored 0–6), and reflex tests (absence of pinna, corneal, startle reflexes, 1 point each; seizures/myoclonus, 1 point). The mNSS is widely used to quantify neurological deficits in models of cerebral ischemia and brain injury.

### 5.32 Rotarod Test

Mice were placed on the rotarod at a constant low speed (4 rpm) for 2 min for acclimatization. This was repeated 2–3 times with 10 min intervals to ensure stable performance. Baseline motor ability was assessed at a constant speed. Motor deficits were detected using an accelerating mode (starting at 4 rpm, increasing by 2 rpm per minute). The latency to fall (seconds) or the maximum speed (rpm) endured was recorded. Each mouse was tested three times with at least 10 min intervals, and the average was calculated. A maximum test duration (300 s) was set. The compartment was wiped with 75% ethanol after each test.

### 5.33 Morris Water Maze Test

During training, the platform was submerged 1 cm below the water surface in the center of the pool. Each animal underwent three consecutive trials: first placed on the platform for 20 s. Starting points (North, South, East, West) were randomized. Animals were gently introduced into the water facing the pool wall. They were allowed to swim/search for the platform for a maximum of 60 s. Latency to find the platform was recorded. If an animal failed to find the platform within 60 s, it was guided there and allowed to rest for 15 s.

### 5.34 Open Field Test

The test was conducted in a square open arena (50 × 50 × 35 cm), divided into a center zone and a periphery zone, with a camera mounted above. Animals were habituated to the test room for 1–2 days before testing. During the test, the animal was placed in the center of the arena, and its behavior was recorded for 5–10 min. Parameters measured included total distance moved, velocity, time spent in the center vs. periphery, and rearing frequency. The arena was cleaned between trials to remove odor cues. Data were analyzed using specialized software to assess locomotor activity, anxiety‐like behavior, and motor coordination.

### 5.35 Elevated Plus Maze

Anxiety‐like behavior post‐injury was assessed using the Elevated Plus Maze as previously described [56]. The maze consists of two open arms and two enclosed arms. Mice were placed in the central area facing an open arm and allowed to explore freely for 5 min. The number of entries into and time spent in the open arms were recorded using the SMART tracking system for analysis.

### 5.36 Forelimb Placing Test

Behavioral tests were performed on day 3 after ICH induction by an investigator blinded to experimental groups. All mice were acclimated to the testing room for 30 min prior to measurements. Mice were held by the torso to suspend forelimbs freely. The whiskers on one side were gently brought into contact with the edge of a table to trigger the forelimb placing reflex. Ten trials were performed for each mouse. The forelimb placing score was calculated as the percentage of successful contralateral forelimb placements. Lower percentages indicate more severe sensorimotor dysfunction.

### 5.37 Corner Turn Test

Behavioral tests were performed on day 3 after ICH induction by an investigator blinded to experimental groups. All mice were acclimated to the testing room for 30 min prior to measurements. A 30° corner arena was built with two plastic boards. Each mouse was placed facing inward toward the corner. After the whiskers contacted the boards, the mouse reared and turned to exit. Ten valid trials were performed with an interval of at least 30 s between trials. The corner turn score was defined as the percentage of turns toward the ipsilateral (hematoma) side. Higher scores represent severe motor asymmetry.

### 5.38 Immunofluorescence Staining

For in vitro NETs analysis, neutrophils (5 × 10^5^ cells) were seeded onto poly‐L‐lysine (Sigma, USA) coated coverslips in 24‐well plates. Cells were fixed with 4% PFA for 10 min and blocked with immunostaining blocking buffer for 30 min. Primary antibodies were applied overnight at 4°C: anti‐Histone H3 (1:1000, ab5103, Abcam), anti‐Myeloperoxidase (MPO) (1:1000, 22225‐1‐AP, Proteintech), anti‐Ly6G (1:1000; sc‐53515, Santa Cruz Biotechnology), and anti‐Neutrophil Elastase (NE) (1:50, 27642‐1‐AP, Proteintech). Appropriate secondary antibodies (1:200, Proteintech) were applied. To visualize NETs morphology, neutrophil‐coated coverslips were incubated with 1 µм SYTOX Green dye (Beyotime, China) for 15 min. To assess the effect of extracellular histones on LMP, BV2 cells were stained with anti‐CTSC (1:100, sc‐74590, Santa Cruz Biotechnology), anti‐LAMP1 (1:100, sc‐20011, Santa Cruz Biotechnology), and anti‐Galectin‐3 (1:50, 14979‐1‐AP, Proteintech) antibodies followed by appropriate secondary antibodies.

For immunofluorescence on paraffin‐embedded brain sections, sections were deparaffinized and underwent antigen retrieval. To assess neutrophil infiltration, sections were incubated with anti‐Ly6G antibody (1:100, sc‐53515, Santa Cruz Biotechnology) overnight at 4°C. To analyze NET components in brain tissue, sections were incubated with anti‐Histone H3 (1:1000, ab5103, Abcam), anti‐MPO (1:1000, 22225‐1‐AP, Proteintech). To elucidate CTSC distribution, sections were stained with anti‐CTSC, anti‐Iba1 (1:100, sc32725, Santa Cruz Biotechnology), anti‐IL‐1β (1:1000, ab234437, Abcam), anti‐TMEM119 (1:100, ab210405, Abcam), anti‐CD31 (1:100, ab222783, Abcam), anti‐Olig2 (1:100, ab109186, Abcam) with corresponding secondaries. Finally, cells or sections were counterstained with Hoechst 33342 and imaged using an Olympus microscope.

### 5.39 Western Blotting

Cells were lysed on ice for 15 min using RIPA lysis buffer containing protease inhibitors. Frozen tissues (20 mg) were homogenized in RIPA buffer using a high‐speed low‐temperature tissue grinder, followed by further lysis on ice for 15 min. Lysates were centrifuged at 12 000 g for 15 min at 4°C, and supernatants were collected. Protein concentration was determined using a BCA protein assay kit (Beyotime, China). Proteins were separated by SDS‐PAGE and transferred to PVDF membranes. After blocking, membranes were incubated with primary antibodies overnight at 4°C. Proteins from neutrophils and brain tissue were probed with anti‐Histone H3 and anti‐PAD4 (1:1000, 17373‐1‐AP, Proteintech). Proteins from BV2 cells and brain tissue were probed with anti‐TNF‐α (1:1000, ab183218, Abcam), anti‐IL‐1β, anti‐CTSC, anti‐CTSS (1:1000, sc74429, Santa Cruz Biotechnology), anti‐CitH3, and anti‐PAD4 antibodies. To assess CTSC expression in lysosomal or cytoplasmic fractions, a Lysosome Isolation Kit (Sigma, USA) was used based on differential centrifugation density gradient separation, following the manufacturer's instructions. After obtaining lysosomal and cytoplasmic fractions, protein extraction was performed as above, and samples were probed with anti‐CTSC antibody. Anti‐GAPDH (total protein loading control), anti‐β‐actin (total protein loading control), and anti‐LAMP1 (lysosomal marker) were used as internal controls. For Western blot analysis, equal amounts of protein were separated by SDS‐PAGE on a single gel and transferred to a PVDF membrane. To achieve optimal signal for proteins, the membrane was horizontally cut into two separate regions. Each region was incubated with the corresponding primary antibody, followed by an HRP‐conjugated secondary antibody, and visualized with enhanced chemiluminescence (ECL) using appropriate exposure times. The blots were then spliced together for figure presentation, with splicing boundaries clearly marked in the figure and noted in the legend.

### 5.40 Quantitative Real‐Time qPCR

Total RNA was extracted from mouse brain tissues using TRIzol reagent (15596026, Invitrogen) according to the manufacturer's instructions. RNA purity and concentration were determined using a OneDrop spectrophotometer (Thermo, USA). First‐strand cDNA was synthesized using an S1000 Thermal Cycler (CS012229, BiO‐Rad, USA) following the provided protocol. PCR amplification and real‐time fluorescence detection were performed using a C1000 Touch Thermal Cycler (CT009555, BiO‐Rad, USA). Relative expression of target genes was calculated using the 2^(‐ΔΔCT) method, with ACTB as the reference gene. The qRT‐PCR primers used in this study were synthesized by Tsingke Biotechnology Co., Ltd. (Beijing) and are listed in Table S5.

### 5.41 ELISA Analysis

Levels of Tumor Necrosis Factor‐alpha (TNF‐α), Interleukin‐1 beta (IL‐1β) and IL‐6 in cell cultures, mouse brain tissues and blood were detected using mouse TNF‐α (JL10484, Jonln), IL‐1β (JL18442, Jonln) ELISA kits and IL‐6 (MM‐1011M2, MEIMIAN) respectively. Additionally, CTSC levels in mouse brain tissue were measured using a mouse CTSC ELISA kit (JL41913, Jonlnbio). Cell‑free culture supernatants were harvested, centrifuged at 3000 × g for 10 min at 4°C to remove cell debris, and stored at −80°C to quantify NET‑related markers. Extracellular dsDNA was measured using the PicoGreen dsDNA assay kit (P9740, Solarbio), while MPO‑DNA complex levels were detected by a commercial sandwich ELISA kit (MM‐1177M2, MEIMIAN). All procedures were performed strictly according to the manufacturers’ instructions.

### 5.42 Statistical Analysis

Statistical analysis was performed using GraphPad Prism 10 software. For comparisons among multiple groups, one‐way ANOVA was used in combination with Dunnett's multiple comparison test; for comparisons between two groups affected by a single variable, Student's *t*‐test was employed; for comparisons among multiple groups involving two factors, two‐way ANOVA was utilized. Specific information on sample size (representing biological replicates) is provided in the corresponding figure legends. Survival analysis was conducted using the Kaplan‐Meier method, and survival curves were generated in Prism software using the Gehan–Breslow–Wilcoxon test. Data are presented as mean ± standard deviation or individual data points. All experiments were independently repeated at least three times, and the statistical significance threshold was set at *p* < 0.05. A *p*‐value > 0.05 indicates no significant difference, and the specific significance levels are marked with asterisks (^*^ *p* < 0.05, ^**^ *p* < 0.01, ^***^ *p* < 0.001), which were determined by the appropriate tests in GraphPad Prism 10.

## Author Contributions

**Junjun Meng** conceived the project and designed the study. **Junjun Meng**, **Jianhua Wang**, **Tao Zhang**, **Xiaofan Fan**, **Jinxiu Guo**, **Shiyuan Zhao**, and **Wenxue Sun** participated in the experiments. **Junjun Meng**, **Jianhua Wang**, and **Xiaofan Fan** were responsible for data acquisition. **Junjun Meng**, **Jianhua Wang**, and **Tao Zhang** performed the data analysis. **Junjun Meng** and **Pei Jiang** wrote the manuscript.

## Conflicts of Interest

The authors declare no conflicts of interest.

## Supporting information




**Supporting File**: advs78538‐sup‐0001‐SuppMat.doc.

## Data Availability

The data that support the findings of this study are available from the corresponding author upon reasonable request.
