# Supplementary material for: A Novel Bioactive Component, Efoba, From Aged Garlic Targets CTSC to Suppress NET‐Driven Neuroinflammation and Ameliorate Secondary Brain Injury After Intracerebral Hemorrhage
Source: Adv Sci (Weinh). 2026 Oct 29:e78538. Online ahead of print. doi: 10.1002/advs.78538 (PMC13720264; doi:10.1002/advs.78538)
Supplement: Supplementary file 1 [file ADVS-9999-e78538-s001.doc]

**Supplementary Material**

**A Novel Bioactive Component Efoba from Aged Garlic Targets CTSC to Suppress NETs-Driven Neuroinflammation and Ameliorate Secondary Brain Injury after Intracerebral Hemorrhage**

Junjun Meng, Jianhua Wang, Tao Zhang, Xiaofan Fan, Jinxiu Guo, Shiyuan zhao, Wenxue Sun, Pei Jiang*

Translational Pharmaceutical Laboratory, Jining NO.1 People’s Hospital, Shandong First Medical University, Jiankang Road, Jining 272000, China.

**Content**

1. Figure S1 to S10;
2. Table S1 to S7;

**FIGURE S1** Structural elucidation and molecular data of Efoba. (A) High-resolution mass spectrum (HR-MS) of Efoba. (B) Infrared spectrum (IR) of Efoba. (C) ^1^H NMR (600 MHz, DMSO, 25℃, TMS) spectrum of Efoba. (D) ^13^C NMR (150 MHz, DMSO, 25℃, TMS) spectrum of Efoba. (E) 2D COSY spectrum of Efoba. (F) 2D HSQC spectrum of Efoba. (G) 2D HMBC spectrum of Efoba. (H) 2D NOESY spectrum of Efoba.

**FIGURE S2** In vivo toxicity evaluation of Efoba. (A) Workflow of the mouse toxicity study. (B) Body weight changes of mice in each group over 5 weeks (n = 6 per group). (C) H&E staining of major organs (heart, liver, spleen, lung, kidneys) from mice in each group indicates that Efoba exhibits negligible toxicity. Scale bar = 50, 100 and 200 μm. (D, E) Serial brain sections were prepared to evaluate hematoma volume following ICH. (F) Detection of Efoba content in brain tissue confirms its ability to cross the blood-brain barrier and enter the brain (n = 6 per group). (G) Representative chromatogram of Efoba detected in brain tissue (n = 4 per group). Data are shown as the mean values ± SD. Statistical significance was determined by ANOVA. ^#^ ICH group compared with control group, * Efoba-treated group compared with ICH group. *^/#^*p* < 0.05, **^/##^*p* < 0.01, ***^/###^*p* < 0.001, ****^/####^*p* < 0.0001, “ns” means no significant difference (*p* > 0.05).

**FIGURE S3** Efoba protects blood-brain barrier integrity and reduces white matter injury after ICH. (A) Flowchart of the mouse experimental protocol. (B) Survival curve of mice within 7 days after ICH (n = 8 per group). (C-D) Monitoring of cerebral blood flow in mice within 7 days after ICH (n = 6 per group). (E-G) Western blot analysis of ZO-1 and Occludin protein expression in mouse brain tissue (n = 3 per group). (H) Transmission electron microscopy (TEM) observation of tight junction structures and BBB disruption in brain tissue (n = 3 per group). Scale bar = 2 and 5 μm. (I-K) TEM observation of myelin sheath damage in brain tissue (n = 3 per group). Scale bar = 2 μm. (L) Measurement of brain water content in mice (n = 4 per group). Data are shown as the mean values ± SD. Statistical significance was determined by ANOVA. ^#^ ICH group compared with control group, * Efoba-treated group compared with ICH group. *^/#^*p* < 0.05, **^/##^*p* < 0.01, ***^/###^*p* < 0.001, ****^/####^*p* < 0.0001, “ns” means no significant difference (*p* > 0.05).

**FIGURE S4** Infiltration of immune cells and inflammatory factors in brain tissue following ICH. (A) Volcano plot of differential genes among ICH group and sham group (n = 5 per group). (B) KEGG enrichment analysis of differential genes among ICH group and sham group (n = 5 per group). (C) Heatmap showing gene expression patterns across ICH group and sham group based on mRNA sequencing data (n = 5 per group). (D, E) Representative immunofluorescence staining for MPO (red) in the cortex at 3 days after ICH. Nuclei were counterstained with DAPI (blue). Scale bar = 50 μm, scale bar = 10 μm (for magnified images). (F, G) Representative immunofluorescence staining for IL-1β (green) in the cortex at 3 days after ICH. Nuclei were counterstained with DAPI (blue). Scale bar = 50 μm, scale bar = 10 μm (for magnified images). Data are shown as the mean values ± SD (n = 3 per group). Statistical significance was determined by ANOVA. ^#^ ICH group compared with control group, * Efoba-treated group compared with ICH group. *^/#^*p* < 0.05, **^/##^*p* < 0.01, ***^/###^*p* < 0.001, ****^/####^*p* < 0.0001, “ns” means no significant difference (*p* > 0.05).

**FIGURE S5** Proteomic analysis of mouse brain tissue following ICH. **(**A) Heatmap showing protein expression patterns across ICH group and sham group based on proteomics data. (B) Volcano plot of differential proteins among ICH group and sham group. (C) GO (Gene Ontology) analysis of differential proteins among ICH group and sham group. (D) KEGG enrichment analysis of differential proteins among ICH group and sham group. (E) Heatmap showing protein expression patterns across Efoba group and ICH group based on proteomics data. (F) GO (Gene Ontology) analysis of differential proteins among Efoba group and ICH group.

**FIGURE S6** Efoba preserves microglial cell viability and suppresses CTSC expression after ICH. (A) Immunohistochemistry (IHC) showing the distribution and expression of CTSC in brain tissue following ICH. Scale bar = 2 mm, scale bar = 50 μm (for magnified images). (B) Effect of Efoba on BV2 cell viability assessed by MTT assay. (C) Effect of Efoba on primary microglial cell viability. (D-E) Expression of CTSC in microglia in an in vitro model (OxyHb stimulated BV2 cells). Data are shown as the mean values ± SD (n = 3 per group). Statistical significance was determined by ANOVA. ^#^ ICH group compared with control group, * Efoba-treated group compared with ICH group. *^/#^*p* < 0.05, **^/##^*p* < 0.01, ***^/###^*p* < 0.001, ****^/####^*p* < 0.0001, “ns” means no significant difference (*p* > 0.05).

**FIGURE S7** Efoba disrupts the CTSC-NET inflammatory microenvironment and reshapes the cellular landscape after ICH. (A) Nearest neighbor distance analysis of inflammation-related molecules. (B) Enrichment analysis of inflammatory pathways and molecular colocalizations. (C) Cellular subpopulation composition analysis. Data are shown as the mean values ± SD (n = 3 per group). Statistical significance was determined by ANOVA. ^#^ ICH group compared with control group, * Efoba-treated group compared with ICH group. *^/#^*p* < 0.05, **^/##^*p* < 0.01, ***^/###^*p* < 0.001, ****^/####^*p* < 0.0001, “ns” means no significant difference (*p* > 0.05).

**FIGURE S8** Efoba confers neuroprotection by inhibiting microglia-derived CTSC-mediated NET formation. (A) Representative images of NET formation stained with SYTOX Green, MPO (red) and CitH3 (green). Nuclei were counterstained with DAPI (blue). Scale bars: 50 μm. (B) Representative images of NET formation stained with SYTOX Green, MPO (red) and CitH3 (green), as well as the detection of ROS production. Nuclei were counterstained with DAPI (blue). Scale bars: 50 μm (overview). (C, D) Neutrophil viability was determined using the CCK-8 assay. (E) ELISA quantification of dsDNA in the supernatants of neutrophil treated with PMA (n = 3). (F) ELISA quantification of MPO-DNA complexes in the supernatants of of neutrophil treated with PMA (n = 3). (G) Representative images of NET formation stained with SYTOX Green, MPO (red) and CitH3 (green) of neutrophil treated with OxyHb (n = 3). Nuclei were counterstained with DAPI (blue). Scale bars: 50 μm. (H) ELISA quantification of dsDNA in the supernatants of neutrophil treated with OxyHb (n = 3). (I) ELISA quantification of MPO-DNA complexes in the supernatants of neutrophil treated with OxyHb (n = 3). Data are shown as the mean values ± SD (n = 3). Statistical significance was determined by ANOVA. ^#^ ICH group compared with control group, * Efoba-treated group compared with ICH group. *^/#^*p* < 0.05, **^/##^*p* < 0.01, ***^/###^*p* < 0.001, ****^/####^*p* < 0.0001, “ns” means no significant difference (*p* > 0.05).

**FIGURE S9** Efoba confers neuroprotection by inhibiting microglia-derived CTSC-mediated NET formation. (A, B) Representative images of NET formation stained with SYTOX Green, MPO (red), CitH3 (green), and NE (pink) of pretreatment experiments. Nuclei were counterstained with DAPI (blue). Scale bars: 50 μm (overview). (C) The levels of CTSC in the pretreatment experiments were detected using ELISA (n = 3). (D) ELISA quantification of dsDNA in the supernatants of pretreatment experiments (n = 3). (E) ELISA quantification of MPO-DNA complexes in the supernatants of pretreatment experiments (n = 3). (F-H) Protein levels of CitH3 and PAD4 were detected using western blot of pretreatment experiments. (I-K) Protein levels of CitH3 and PAD4 were detected using western blot of conditioned medium (CM) experiments. (L, M) Western blot validation of CTSC protein expression in CTSC‑KO samples. (N-P) Protein levels of CitH3 and PAD4 were detected using western blot of CTSC-KO experiments. (Q) Representative images of NET formation after CTSC overexpression, stained with MPO (red), and NE (green). Nuclei were counterstained with DAPI (blue). Scale bars: 50 μm. Data are shown as the mean values ± SD (n = 3). Statistical significance was determined by ANOVA. ^#^ ICH group compared with control group, * Efoba-treated group compared with ICH group. *^/#^*p* < 0.05, **^/##^*p* < 0.01, ***^/###^*p* < 0.001, ****^/####^*p* < 0.0001, “ns” means no significant difference (*p* > 0.05).

**FIGURE S10** CTSC expression in brain tissue following ICH. (A) The content of CTSC in mice brain tissues at different time points after ICH were detected by ELISA (n = 4). (B-D) Western blot detection of CitH3 and Ly6G protein expression in brain tissues at different time points after ICH (n = 3). (E, F) Representative images of CTSC expression in brain tissue (n = 3), Scale bars: 50 μm. Data are shown as the mean values ± SD (n = 3). Statistical significance was determined by ANOVA. # ICH group compared with control group, * Efoba-treated group compared with ICH group. */#p < 0.05, **/##p < 0.01, ***/###p < 0.001, ****/####p < 0.0001, “ns” means no significant difference (p > 0.05).

**Table S1. Characteristic IR absorption peaks and their corresponding functional groups/vibration modes.**

| **Functional Group Type** | **Characteristic Peak Wavenumber (cm⁻¹)** | **Vibration Mode** | **Corresponding Molecular Structure Position** |
| --- | --- | --- | --- |
| Phenolic Hydroxyl Ar-OH | 3531.74, 3368.93 | O-H Stretching Vibration | Hydroxyl groups on the two benzene rings |
| Aromatic C-H | 3071.13, 3028.24 | C-H Stretching Vibration | Hydrogen atoms on the two benzene rings |
| Methoxy Group -OCH₃ | 2935.88, 2845.35 | C-H Stretching Vibration | Methoxy groups on the benzene ring |
| Ester Group C=O | 1704.91 | C=O Stretching Vibration | Ester carbonyl group (-COO-) |
| Carbon-Carbon Double Bond | 1680.1 | C=C Stretching Vibration | Carbon-carbon double bond |
| Benzene Ring Skeleton | 1600.63, 1514.40 | Skeleton Stretching Vibration | The two benzene rings |
| Ester Group C-O | 1283.58, 1166.05 | C-O Stretching Vibration | C-O bond of the ester group |
| Aromatic C-H Out-of-Plane Bending | 981.34, 841.34 | Out-of-Plane Bending Vibration | Ortho-substituted C-H on the benzene ring |

| **Table S2. Hematological parameters** | | | | | |
| --- | --- | --- | --- | --- | --- |
| Parameter | Abbreviation | Control | 40 mg/kg | 480 mg/kg | 960 mg/kg |
| White blood cell count | WBC (10^9^/L) | 7.62 ± 0.26 | 10.17 ± 0.16 | 7.62 ± 0.17 | 6.23 ± 0.12 |
| Lymphocyte count | Lymph# (10^9^/L) | 5.65 ± 0.16 | 8.07 ± 0.16 | 6.15 ± 0.24 | 4.73 ± 0.12 |
| Monocyte count | Mon# (10^9^/L) | 0.28 ± 0.08 | 0.27 ± 0.05 | 0.20 ± 0.00 | 0.15 ± 0.05 |
| Granulocyte count | Gran# (10^9^/L) | 1.83 ± 0.14 | 1.83 ± 0.05 | 1.27 ± 0.08 | 1.35 ± 0.05 |
| Lymphocyte percentage | Lymph (%) | 75.07 ± 2.82 | 79.33 ± 0.33 | 80.68 ± 1.36 | 75.90 ± 0.67 |
| Monocyte percentage | Mon (%) | 3.23 ± 0.78 | 2.50 ± 0.43 | 2.62 ± 0.08 | 2.53 ± 0.75 |
| Granulocyte percentage | Gran (%) | 22.30 ± 1.64 | 18.17 ± 0.66 | 16.70 ± 1.32 | 21.57 ± 1.30 |
| Red blood cell count | RBC (10^12^/L) | 9.12 ± 0.07 | 9.96 ± 0.10 | 9.24 ± 0.41 | 8.57 ± 0.22 |
| Hemoglobin | HGB (g/L) | 128.00 ± 0.89 | 141.00 ± 1.67 | 128.00 ± 5.10 | 119.80 ± 2.64 |
| Hematocrit | HCT (%) | 46.65 ± 0.43 | 50.33 ± 0.81 | 47.03 ± 2.54 | 44.40 ± 1.10 |
| Mean corpuscular volume | MCV (fL) | 49.12 ± 0.89 | 50.57 ± 0.32 | 50.88 ± 0.49 | 51.85 ± 0.05 |
| Mean corpuscular hemoglobin | MCH (pg) | 13.95 ± 0.08 | 14.13 ± 0.05 | 13.82 ± 0.08 | 13.95 ± 0.05 |
| Mean corpuscular hemoglobin concentration | MCHC (g/L) | 275.50 ± 1.23 | 280.20 ± 1.47 | 272.30 ± 4.03 | 269.70 ± 0.82 |
| Red cell distribution width | RDW (%) | 13.20 ± 0.06 | 13.88 ± 0.18 | 13.45 ± 0.29 | 14.00 ± 0.17 |
| Platelet count | PLT (10^9^/L) | 963.80 ± 7.52 | 1025.00 ± 37.52 | 1142.00 ± 28.43 | 1186.00 ± 2.32 |
| Mean platelet volume | MPV (fL) | 6.05 ± 0.05 | 6.00 ± 0.13 | 6.15 ± 0.05 | 6.20 ± 0.09 |
| Platelet distribution width | PDW | 16.67 ± 0.21 | 16.82 ± 0.13 | 16.37 ± 0.18 | 16.68 ± 0.15 |
| Plateletcrit | PCT (%) | 0.59 ± 0.02 | 0.61 ± 0.03 | 0.70 ± 0.02 | 0.74 ± 0.01 |

| **Table S3. Changes of biochemical parameters in serum of mice.** | | | | |
| --- | --- | --- | --- | --- |
| **Parameter** | **Control** | **40 mg/kg** | **480 mg/kg** | **960 mg/kg** |
| ALT (U/L) | 40.00 ± 5.53 | 45.94 ± 2.83 | 46.95 ± 2.34 | 58.66 ± 2.22 |
| AST (U/L) | 149.80 ± 1.99 | 148.80 ± 1.95 | 154.60 ± 1.78 | 230.40 ± 4.99 |
| ALP (U/L) | 147.30 ± 8.46 | 143.50 ± 7.49 | 154.50 ± 8.01 | 165.00 ± 5.37 |
| ALB (U/L) | 27.29 ± 3.05 | 27.67 ± 3.56 | 27.63 ± 3.64 | 27.16 ± 1.75 |
| BUN (mg/dl) | 25.05 ± 3.57 | 25.70 ± 2.82 | 26.53 ± 3.06 | 26.14 ± 4.05 |
| Cr (μmol/L) | 35.15 ± 6.35 | 29.32 ± 2.94 | 32.69 ± 6.06 | 31.03 ± 5.47 |
| UA (μmol/L) | 59.75 ± 4.14 | 59.63 ± 0.90 | 60.85 ± 2.55 | 59.50 ± 3.82 |

| **Table S4. Changes of coagulation parameters and cytokine levels in plasma of mice.** | | | | |
| --- | --- | --- | --- | --- |
| **Parameter** | **Control** | **40 mg/kg** | **480 mg/kg** | **960 mg/kg** |
| PT (s) | 13.18 ± 1.15 | 12.70 ± 0.63 | 13.08 ± 1.10 | 12.78 ± 1.11 |
| APTT (s) | 42.58 ± 6.26 | 44.03 ± 6.59 | 44.47 ± 5.05 | 43.70 ± 4.50 |
| TT (s) | 28.64 ± 9.01 | 27.75 ± 6.13 | 28.10 ± 5.31 | 28.23 ± 6.91 |
| FIB (g/L) | 1.88 ± 0.26 | 1.80 ± 0.41 | 1.86 ± 0.14 | 1.88 ± 0.26 |
| TNF-α (pg/mL) | 9.65 ± 0.72 | 9.03 ± 0.78 | 9.04 ± 1.15 | 9.41 ± 0.88 |
| IL-6 (pg/mL) | 8.26 ± 0.23 | 8.27 ± 0.25 | 8.18 ± 0.22 | 8.20 ± 0.32 |

**Table S5. Primer sequences used in qRT-PCR analysis.**

| **Gene** | **Forward (5’---3’)** | **Reverse (5’---3’)** |
| --- | --- | --- |
| CD86 | GGAACAACTGGACTCTACGAC | GTTTCGGGTGACCTTGCTTA |
| iNOS | GTGTCAGTGGCTTCCAGCTC | CTCATGCGGCCTCCTTTGAG |
| TNF-α | GGACTAGCCAGGAGGGAGAACAG | GCCAGTGAGTGAAAGGGACAGAAC |
| IL-1β | TCGCAGCAGCACATCAACAAGAG | AGGTCCACGGGAAAGACACAGG |
| CTSC | CTGCTGGTGCTTTTGGGAGTCTG | TGTCGCTTCGGGAACTTCTAGGG |

| **Table S6. Differential peptide profiles identified via Lip-MS spectrometry.** | | | | | |
| --- | --- | --- | --- | --- | --- |
| **Gene Name** | **Accession** | **Sequence** | **P-VALUE** | **Fold Change** | **Log_Fold Change** |
| Eps15 | P42567 | AQLEEQLQEVR_27868 | 0.000613345 | 1808.685868 | 10.82072615 |
| Arhgef2 | Q60875 | GHFNDESPLGLR_28933 | 0.180704224 | 868.2539156 | 9.761973202 |
| Rpl7 | P14148 | AGNFYVPAEPK_27768 | 0.033883068 | 471.1545808 | 8.88005666 |
| Ppp4c | P97470 | EILVEESNVQR_27883 | 0.033478391 | 398.8432176 | 8.639677934 |
| Ampd2 | Q9DBT5 | STDDPLQFHFTK_28439 | 0.000261961 | 373.7482517 | 8.545923021 |
| Hadha | Q8BMS1 | THINYGVK_29019 | 0.001088229 | 347.5781747 | 8.441193684 |
| Adsl | P54822 | CYVGDNTDLIILR_27277 | 0.089849799 | 337.7216122 | 8.399690696 |
| Hspe1 | Q64433 | PEYGGTK_29371 | 0.169293364 | 286.0987448 | 8.160369358 |
| Nup160 | Q9Z0W3 | TLQQEFWCK_28841 | 0.033975013 | 211.274763 | 7.722976636 |
| Pdia4 | P08003 | GRPFDYNGPR_28100 | 0.015108116 | 204.6529239 | 7.677035469 |
| Fermt3 | Q8K1B8 | DEAPGDPTQQLNLK_28227 | 0.002050684 | 201.5139771 | 7.654736098 |
| Gnai2 | P08752 | SAYDLVLAEDEEMNR_29242 | 0.083337135 | 197.9728164 | 7.629158538 |
| Asl | Q91YI0 | NDQVVTDLR_28780 | 0.025059562 | 194.4474087 | 7.603236199 |
| Gvin1 | Q80SU7 | VLVIDTEGLR_28941 | 0.179455515 | 188.761516 | 7.560420853 |
| Psma3 | O70435 | AVENSSTAIGIR_28354 | 0.116184532 | 188.0074735 | 7.554646201 |
| Vcp | Q01853 | RSVSDNDIR_27335 | 0.122975171 | 173.8245573 | 7.441488105 |
| Ctsc | P97821 | ILNLPESWDWR_28719 | 0.015747843 | 166.7457608 | 7.381506274 |
| Plod3 | Q9R0E1 | LLLLDYPPDR_28488 | 0.034319839 | 141.3386771 | 7.1430125 |
| Rrm2 | P11157 | EALKPDER_28198 | 0.039131613 | 136.0399977 | 7.087887077 |
| Hint1 | P70349 | QVAQPGGDTIFGK_28195 | 0.159101593 | 135.686001 | 7.084128073 |
| Rsu1 | Q01730 | LTTVPPNVAELK_27597 | 0.01427952 | 128.7619575 | 7.008562605 |
| Gsk3b | Q9WV60 | NLLLDPDTAVLK_28945 | 0.117667685 | 125.3212111 | 6.969486807 |
| Bin2 | D3Z6Q9 | EGAGSSGPEEPR_27913 | 0.001136668 | 124.7865684 | 6.963318846 |
| Timeless | Q9R1X4 | AGLGPTEEEATGEEEWNSAPK_27757 | 0.002517856 | 124.5688865 | 6.960799962 |
| **Table S6. (Continued) Differential peptide profiles identified via Lip-MS spectrometry.** | | | | | |
| **Gene Name** | **Accession** | **Sequence** | **P-VALUE** | **Fold Change** | **Log_Fold Change** |
| Rrbp1 | Q99PL5 | LQSSEVEVK_27243 | 0.000739299 | 121.2449284 | 6.921780591 |
| Ppa1 | Q9D819 | VDDPDAANYK_27909 | 0.000563858 | 115.931784 | 6.857132342 |
| Hk2 | O08528 | SHEQLLEVK_28251 | 0.005479469 | 107.7796478 | 6.751940967 |
| Pgk1 | P09411 | SGIPAGWMGLDCGTESSK_27447 | 0.097330776 | 107.0512882 | 6.742158347 |
| Get3 | O54984 | QLVFPDPEKPCK_28759 | 0.001047833 | 105.407883 | 6.719838954 |
| Cdk6 | Q64261 | ILDIIGLPGEEDWPR_27488 | 0.014231646 | 103.6480344 | 6.695548947 |
| Naa15 | Q80UM3 | GCPPVFNTLR_27931 | 0.118062321 | 100.9263024 | 6.657158394 |
| Iars1 | Q8BU30 | FLYNEHTVR_28172 | 0.022338541 | 100.5567714 | 6.651866424 |
| Psme3 | P61290 | YPHVEDYR_29378 | 0.004383624 | 95.14685553 | 6.572084073 |
| Gtf2i | Q9ESZ8 | TPVKEDWNVR_28042 | 0.061802292 | 94.51283841 | 6.56243841 |
| Sec63 | Q8VHE0 | YSGDQILIR_29529 | 0.015081274 | 93.51071333 | 6.547059756 |
| Cct2 | P80314 | FDHPELVK_27283 | 0.050104596 | 90.46938716 | 6.499357793 |
| Rbm8a | Q9CWZ3 | NIHLNLDR_28532 | 0.215944201 | 89.57028833 | 6.484948345 |
| Msh2 | P43247 | QSLQEDLLR_28283 | 0.001850678 | 86.85729718 | 6.440575155 |
| Pfkl | P12382 | QQGGAPTPFDR_28090 | 0.051251523 | 85.73630549 | 6.421834345 |
| Numa1 | E9Q7G0 | LSQLEDQATR_27997 | 0.040329859 | 81.90780034 | 6.355928946 |
| Shmt2 | Q9CZN7 | EIPYTFEDR_27966 | 0.045646731 | 80.6647544 | 6.333866536 |
| Mrpl45 | Q9D0Q7 | SLEPAQVVHVR_29150 | 0.01672298 | 80.25954326 | 6.326601041 |
| Sh3bgrl | Q9JJU8 | KIGFEEK_28580 | 0.067737519 | 79.64033671 | 6.315427415 |
| Fkbp5 | Q64378 | VLAVNPQNR_28791 | 0.060336704 | 78.54684049 | 6.29548134 |
| Oxct1 | Q9D0K2 | DEADADLINAGK_28136 | 0.03880988 | 75.84998854 | 6.245077057 |
| Pfn1 | P62962 | SLLQDGEFTMDLR_27431 | 0.032348582 | 72.17642556 | 6.173455792 |
| Trpv2 | Q9WTR1 | ILPLLQIDR_29274 | 0.00054626 | 71.71937134 | 6.164290937 |
| Flii | Q9JJ28 | GYLELPQINYK_29249 | 0.125204434 | 68.58944956 | 6.099914773 |
| **Table S6. (Continued) Differential peptide profiles identified via Lip-MS spectrometry.** | | | | | |
| **Gene Name** | **Accession** | **Sequence** | **P-VALUE** | **Fold Change** | **Log_Fold Change** |
| Aifm2 | Q8BUE4 | VSNLEELPR_29615 | 0.065822724 | 66.49731234 | 6.055224126 |
| Ppm1a | P49443 | TGFLEIDEHMR_29248 | 0.063807705 | 66.34378937 | 6.051889512 |
| Cse1l | Q9ERK4 | DLEGSDIDTR_27217 | 0.063821495 | 65.64010148 | 6.036505564 |
| Ctsz | Q9WUU7 | TYPRPHEYLSPADLPK_28720 | 0.130942047 | 64.33155292 | 6.00745461 |
| Cstf2 | Q8BIQ5 | AGLPVRDPAVDR_21381 | 0.049367433 | 63.45180287 | 5.987589251 |
| Icam1 | P13597 | TFDLPATIPK_27429 | 0.094582234 | 61.80114493 | 5.949561661 |
| Thoc6 | Q5U4D9 | SPEVLSGGEDGAVR_29216 | 0.099137957 | 61.75436886 | 5.9484693 |
| Flna | Q8BTM8 | YGGHQVPGSPFK_27629 | 0.032309804 | 59.46131735 | 5.893879521 |
| Capg | P24452 | NTLLGERPVQHR_28223 | 0.000143684 | 59.19860018 | 5.887491157 |
| Ddx52 | Q8K301 | FTEDDKPLLR_28701 | 0.005015149 | 53.57943897 | 5.74360757 |
| Smc3 | Q9CW03 | IKLEGIITR_29165 | 0.053815689 | 51.19725463 | 5.677994545 |
| Taok3 | Q8BYC6 | SDLLEVHK_29186 | 0.066824399 | 48.02300155 | 5.585653673 |
| Farp1 | F8VPU2 | SEIGDFDEALDR_29198 | 0.042530989 | 47.9313294 | 5.582897049 |
| Rab18 | P35293 | EVDRNEGLK_29016 | 0.044721384 | 45.82855934 | 5.518175029 |
| Eef1akmt4-Ece2 | P0DPD9 | DFPSGSFDVVLEK_27954 | 0.076590689 | 45.05726258 | 5.493687761 |
| Mcm4 | P49717 | IDVIHYR_27830 | 0.019491063 | 44.56187115 | 5.477737907 |
| Gsn | P13020 | KHVVPNEVVVQR_27752 | 0.00116741 | 43.26133624 | 5.435006322 |
| Fbxo3 | Q9DC63 | NVVSGGFPIIR_27337 | 0.015902491 | 43.22105259 | 5.433662302 |
| Abcf1 | Q6P542 | GYEGQKPLFK_28963 | 0.041591635 | 43.03197616 | 5.42733719 |
| Eif5 | P59325 | ALNRPPTYPTK_28040 | 0.031160139 | 40.32686512 | 5.333669354 |
| Gak | Q99KY4 | FGKPELPR_28542 | 0.003927587 | 39.69761341 | 5.310980371 |
| Pold2 | O35654 | HISPTAPDTLGCYPFYK_29122 | 0.049461478 | 39.15416492 | 5.291093873 |
| Ganab | Q8BHN3 | NHGLYVK_27572 | 0.021448079 | 39.11515515 | 5.289655782 |
| Atic | Q9CWJ9 | YVTGTIGEGEDLVK_27563 | 0.03897139 | 38.29745568 | 5.259176644 |
| **Table S6. (Continued) Differential peptide profiles identified via Lip-MS spectrometry.** | | | | | |
| **Gene Name** | **Accession** | **Sequence** | **P-VALUE** | **Fold Change** | **Log_Fold Change** |
| Rac1 | P63001 | WDTAGQEDYDR_28381 | 0.005732544 | 36.81684444 | 5.202294074 |
| Uqcrh | P99028 | GDPKEEEEEELVDPLTTVR_29193 | 0.003119724 | 36.49097275 | 5.189467705 |
| Gdi2 | Q61598 | RTDDYLDQPCCETINR_28263 | 0.028426718 | 35.89341511 | 5.165647291 |
| Hdgf | P51859 | AAEPEVEPEAHEGDGDK_27252 | 0.000861961 | 35.81831117 | 5.162625411 |
| Aarsd1 | Q3THG9 | DRLPVSVR_28729 | 0.003942585 | 32.88676481 | 5.039435187 |
| Mrpl41 | Q9CQN7 | APAGIDTPLTAK_28130 | 0.167033508 | 32.88565954 | 5.0393867 |
| Pkn1 | P70268 | NLGADSDSSSQK_27319 | 0.127529003 | 30.69014284 | 4.939703455 |
| Rab3gap1 | Q80UJ7 | LTEPAPVPIHK_27305 | 0.097450079 | 28.59494854 | 4.837688404 |
| Snx4 | Q91YJ2 | EEQINEGEQQLK_28858 | 0.025570272 | 26.01184859 | 4.701097026 |
| Ubap2 | Q91VX2 | ESTAGDGPSTVSR_26507 | 0.00354902 | 20.33021511 | 4.345553575 |
| Chd4 | Q6PDQ2 | PPPPPVEVPIRK_28177 | 0.010051476 | 19.04036079 | 4.250988911 |
| Ybx3 | Q9JKB3 | LAGSPGGDAAPGPAPASSAPAGGEDAEK_27186 | 0.002683159 | 18.7278668 | 4.227114673 |
| Dok3 | Q9QZK7 | LLELELDEAGSAGR_16628 | 0.001733537 | 18.07866881 | 4.176216546 |
| Blvra | Q9CY64 | FTASPLEEEK_13658 | 0.000822177 | 10.69217266 | 3.418483135 |
| Eif4g1 | Q6NZJ6 | VVTLEQPANLLR_6644 | 0.008461188 | 10.63333966 | 3.410522877 |
| Mapk3 | Q63844 | PVVPGEVEVVK_26779 | 0.025894726 | 9.995160875 | 3.321229788 |
| Hsp90ab1 | P11499 | LKEFDGK_24909 | 0.001759944 | 9.390297322 | 3.231170838 |
| Uggt1 | Q6P5E4 | LFVDADQIVR_5051 | 0.029478584 | 8.466482049 | 3.081762633 |
| Mrps7 | Q80X85 | EPLIDKEYYR_26479 | 0.03002461 | 8.269313428 | 3.047767552 |
| Trmt5 | Q9D0C4 | DSELFSPPSDVR_22332 | 0.001620887 | 7.52492623 | 2.911677439 |
| Supt6h | Q62383 | GALEEILENPER_20588 | 0.004356592 | 7.293752878 | 2.86666132 |
| Ttll12 | Q3UDE2 | DFAYGEADPLIR_2126 | 0.014229009 | 7.189387467 | 2.845868859 |
| Strap | Q9Z1Z2 | SDSIYSSTPEVK_25941 | 0.006083626 | 6.764313926 | 2.757943616 |
| Tpd52 | Q62393 | EKHLAELK_26432 | 0.021458798 | 6.720192038 | 2.74850246 |
| **Table S6. (Continued) Differential peptide profiles identified via Lip-MS spectrometry.** | | | | | |
| **Gene Name** | **Accession** | **Sequence** | **P-VALUE** | **Fold Change** | **Log_Fold Change** |
| Ndufv2 | Q9D6J6 | AVLPVLDLAQR_1338 | 0.022209793 | 6.64133584 | 2.731473455 |
| Mthfd1 | Q922D8 | GINYVPDDTKPNGR_22421 | 0.036658043 | 6.445119796 | 2.688207174 |
| Isg15 | Q64339 | IGVPAFQQR_16383 | 0.010318556 | 6.381381433 | 2.67386877 |
| Fam3c | Q91VU0 | SPFEQHIK_20231 | 0.016145753 | 6.057273578 | 2.598668572 |
| Cdk7 | Q03147 | APELLFGAR_8973 | 0.010620206 | 5.794943135 | 2.534794504 |
| Smap1 | Q91VZ6 | KEEQQLEPK_19141 | 0.011078195 | 5.709766257 | 2.513431687 |
| Ep300 | B2RWS6 | HDCPVCLPLK_26729 | 0.013627166 | 5.574077255 | 2.478732998 |
| Ssb | P32067 | NANNGNLLLR_15183 | 0.02042013 | 5.541641341 | 2.470313341 |
| Gmfg | Q9ERL7 | TTDDLTETWLK_23280 | 0.009740814 | 5.501231824 | 2.4597547 |
| Nme2 | Q01768 | QHYIDLK_23089 | 0.020585772 | 5.316227555 | 2.410402859 |
| Ppie | Q9QZH3 | KFDDENFILK_17728 | 0.034423709 | 5.115109254 | 2.354765055 |
| Pnkp | Q9JLV6 | LQEHLDPALQR_17053 | 0.024175783 | 5.076569531 | 2.343853932 |
| Eif3l | Q8QZY1 | LDLTEQEFR_16214 | 0.0205877 | 5.017503193 | 2.32696963 |
| Clpp | O88696 | PLIPIVVEQTGR_19991 | 0.043550588 | 5.000929136 | 2.322196162 |
| Oxr1 | Q4KMM3 | LWLDGDLYHGR_23598 | 0.027923357 | 4.982966347 | 2.317004831 |
| Idh3g | P70404 | SNADEEDIR_26794 | 0.019522326 | 4.963841894 | 2.311457164 |
| Basp1 | Q91XV3 | AESTGAADGAAPEEGEAK_20685 | 0.012192992 | 4.954548111 | 2.30875348 |
| Ctps1 | P70698 | LVPQPSSTGEQK_25984 | 0.023785628 | 4.934473328 | 2.30289611 |
| Nsfl1c | Q9CZ44 | EAEPTTNIQIR_12384 | 0.02303714 | 4.89422076 | 2.291079178 |
| Gpi | P06745 | LIHQGTK_21869 | 0.034430467 | 4.818551083 | 2.2685994 |
| Ddx20 | Q9JJY4 | LFILDEADK_24813 | 0.022740187 | 4.786144049 | 2.258863818 |
| Lman2 | Q9DBH5 | DNVDDPTGNFR_5973 | 0.023344557 | 4.631832649 | 2.211583129 |
| Snrnp200 | Q6P4T2 | EEEKLELQK_24841 | 0.034996183 | 4.517432011 | 2.175502888 |
| Ubfd1 | Q78JW9 | LEQDQLWIGTK_19395 | 0.005549931 | 4.513536663 | 2.174258326 |
| **Table S6. (Continued) Differential peptide profiles identified via Lip-MS spectrometry.** | | | | | |
| **Gene Name** | **Accession** | **Sequence** | **P-VALUE** | **Fold Change** | **Log_Fold Change** |
| Rida | P52760 | AAGCDFNNVVK_735 | 0.031161097 | 4.50893166 | 2.172785644 |
| Acad10 | Q8K370 | GGLVISPEGLSPAVR_23969 | 0.032132465 | 4.457588306 | 2.156263378 |
| Hsp90b1 | P08113 | ELMNDIKPIWQR_25534 | 0.032400674 | 4.452602037 | 2.154648673 |
| Nck2 | O55033 | IYDLNIPAFVK_26918 | 0.029038004 | 4.419677724 | 2.143941174 |
| Usb1 | Q91W78 | FLDLVSEVDR_17280 | 0.031429961 | 4.337627165 | 2.116906053 |
| Cad | B2RQC6 | GTSPEAIDSAENR_5785 | 0.032533716 | 4.099303501 | 2.035378807 |
| Gfm1 | Q8K0D5 | AFVQIPIGLEGDFK_20638 | 0.042508978 | 4.089546738 | 2.031940952 |
| Arhgap25 | Q8BYW1 | LPGQDNLVK_11142 | 0.004999106 | 3.984017445 | 1.994223965 |
| Rnh1 | Q91VI7 | ASNELKDEGAR_21729 | 0.029737586 | 3.931221635 | 1.974977702 |
| Arfgef2 | A2A5R2 | TTDLHSPQVK_6876 | 0.015425422 | 3.851938963 | 1.945584843 |
| Ctss | O70370 | QYIIDNGGIEADASYPYK_26198 | 0.045600594 | 3.84758411 | 1.943952865 |
| Cct8 | P42932 | AVDDGVNTFK_21473 | 0.047355451 | 3.829127551 | 1.937015718 |
| Eif3e | P60229 | QLQAETEPIVK_11540 | 0.001806027 | 3.381550206 | 1.757684774 |
| Ddx21 | Q9JIK5 | YEQVDLIGK_23387 | 0.041420445 | 3.286030777 | 1.716345993 |
| Borcs6 | Q9D6W8 | LTPAPTPTIPPIDPDVLR_24480 | 0.028498937 | 3.181146081 | 1.669546623 |
| Vps4b | P46467 | NSLTEADFQELGR_16230 | 0.039709834 | 3.166272008 | 1.6627852 |
| Plpbp | Q9Z2Y8 | GHDLSQGPNPDFQR_17862 | 0.035486424 | 3.109679606 | 1.636765945 |
| Eef1b | O70251 | KPAVVAK_23251 | 0.030259262 | 3.10660535 | 1.635338979 |
| Pfkp | Q9WUA3 | VDGGSNIVEAK_2956 | 0.009053272 | 3.090912077 | 1.628032617 |
| Lsm4 | Q9QXA5 | IPDEIIDMVR_19002 | 0.024670382 | 2.942522367 | 1.557053382 |
| Psmd3 | P14685 | FNQVLDQFGEK_15954 | 0.043218953 | 2.919318941 | 1.545631837 |
| Pcbp2 | Q61990 | SQCGSLIGK_20713 | 0.034208327 | 2.891304006 | 1.531720309 |
| St13 | Q99L47 | ALNDGELQK_6447 | 0.01012147 | 2.685574054 | 1.425230504 |
| Chchd4 | Q8VEA4 | GSDCIDQFR_18295 | 0.035032419 | 2.661080211 | 1.412011997 |
| **Table S6. (Continued) Differential peptide profiles identified via Lip-MS spectrometry.** | | | | | |
| **Gene Name** | **Accession** | **Sequence** | **P-VALUE** | **Fold Change** | **Log_Fold Change** |
| Idi1 | P58044 | AELGIPLEEVDLNEMDYLTR_3866 | 0.000944725 | 2.640241847 | 1.400670087 |
| Snrnp70 | Q62376 | YDERPGPSPLPHR_4857 | 0.001385461 | 2.636588958 | 1.398672674 |
| Prpf38b | Q80SY5 | LDDEEDLDVK_24339 | 0.032069712 | 2.631404247 | 1.395832898 |
| Gsto1 | O09131 | EYLDEAYPEK_15114 | 0.029919088 | 2.619426808 | 1.389251151 |
| Papola | Q61183 | TIPEDLDLRDDSLLK_10508 | 0.002436222 | 2.608003415 | 1.382945759 |
| Glrx3 | Q9CQM9 | HAPELTK_13902 | 0.04358679 | 2.603058445 | 1.380207704 |
| Bag6 | Q9Z1R2 | LQEDPNYSPQR_10259 | 0.026587605 | 2.585330099 | 1.370348498 |
| Vps35 | Q9EQH3 | CFSEENHEPLR_20747 | 0.040398644 | 2.54636465 | 1.348439034 |
| Gapdh | P16858 | IALNDNFVK_9769 | 0.035134931 | 2.443604803 | 1.289010981 |
| Rtn4 | Q99P72 | GPLPAAPPTAPER_8020 | 0.040556176 | 2.38844781 | 1.256073353 |
| Plec | Q9QXS1 | SIQEELQHLR_8510 | 0.045736484 | 2.283343224 | 1.191147737 |
| Cdk1 | P11440 | LESEEEGVPSTAIR_7982 | 0.035064723 | 2.192484115 | 1.13256639 |
| Nolc1 | E9Q5C9 | QNETADEATTPQAK_5806 | 0.004835382 | 2.173421321 | 1.11996787 |
| Mettl16 | Q9CQG2 | EDFGLSIDIPLER_11735 | 0.016931734 | 2.089182746 | 1.062938694 |
| Etfa | Q99LC5 | SDRPELTGAK_14726 | 0.035491386 | 1.984626388 | 0.988867441 |
| Ap1b1 | O35643 | DIANENEAQFQIR_13011 | 0.040724815 | 1.97509226 | 0.981920046 |
| Spcs3 | Q6ZWQ7 | ADLENIFDWNVK_2583 | 0.040485216 | 1.934940149 | 0.952288942 |
| Atl3 | Q91YH5 | IYQGEDLPHPK_15540 | 0.035782406 | 1.890281364 | 0.918600992 |
| Rps24 | P62849 | QMVIDVLHPGK_19241 | 0.00469891 | 1.865448096 | 0.899522219 |
| Vps26a | P40336 | LVLVDEEDRR_15778 | 0.044352044 | 1.859077545 | 0.894586948 |
| Supt16h | Q920B9 | DEVELIHFER_12530 | 0.012237369 | 1.844675241 | 0.883366849 |
| Rangap1 | P46061 | GSGEEPATPSR_9558 | 0.046549757 | 1.818496149 | 0.86274587 |
| Plcg2 | Q8CIH5 | VVNDNGLSPVWAPTQEK_12945 | 0.033173675 | 1.753764921 | 0.810455378 |
| Septin11 | Q8C1B7 | NENHCDFVK_10408 | 0.035795814 | 1.725587968 | 0.787088023 |
| **Table S6. (Continued) Differential peptide profiles identified via Lip-MS spectrometry.** | | | | | |
| **Gene Name** | **Accession** | **Sequence** | **P-VALUE** | **Fold Change** | **Log_Fold Change** |
| Cct3 | P80318 | AMTGVEQWPYR_1860 | 0.020066176 | 1.717563417 | 0.780363368 |
| Iqgap1 | Q9JKF1 | QHYQDTLIR_6785 | 0.006268724 | 1.715109888 | 0.778301014 |
| Mcm6 | P97311 | FLEEFQGSDGEIK_64 | 0.018923789 | 1.713414869 | 0.776874513 |
| Uba7 | Q9DBK7 | TCPLDPTTEDIYDDSFFSR_9482 | 0.00343547 | 1.705084769 | 0.769843465 |
| Mfge8 | P21956 | AFGWYPHLGR_4817 | 0.044529651 | 1.686301753 | 0.753862721 |
| Yars2 | Q8BYL4 | SFSELVLDPGTSVIDTCR_20168 | 0.041719342 | 1.68552279 | 0.753196134 |
| Ogfr | Q99PG2 | QEQVPGEPDPQGVSEVEK_8376 | 0.00843369 | 1.673784393 | 0.7431137 |
| Hdlbp | Q8VDJ3 | VFHVPLEER_3288 | 0.038129672 | 1.569032532 | 0.649875265 |
| Lgals3 | P16110 | VEADHFK_4830 | 0.017235291 | 1.567380279 | 0.64835525 |
| Drg2 | Q9QXB9 | EHEDVIQIVK_10236 | 0.039103451 | 1.557723703 | 0.639439362 |
| Cct5 | P80316 | RTSLGPNGLDK_5201 | 0.04521121 | 1.552435252 | 0.634533099 |
| Plaa | P27612 | TFLGHEDCVR_15667 | 0.036506883 | 1.550124252 | 0.632383861 |
| Usp9x | P70398 | ILTDEAVSGWK_8402 | 0.0332934 | 1.508956849 | 0.59355155 |
| Rpl23 | P62830 | VHPAVVIR_15043 | 0.024974769 | 1.496393793 | 0.581489887 |
| Vim | P20152 | SLPLVDTHSK_1021 | 0.010378032 | 1.478573703 | 0.56420616 |
| Rpn2 | Q9DBG6 | TVDKDQSIAPK_9952 | 0.02007563 | 1.47314516 | 0.558899597 |
| Ruvbl2 | Q9WTM5 | GTEVQVDDIKR_13379 | 0.017116359 | 1.469287765 | 0.55511698 |
| Rap1b | Q99JI6 | YDPTIEDSYR_9610 | 0.022220116 | 1.445662674 | 0.531730958 |
| Vps16 | Q920Q4 | ADLEDWEELEK_1233 | 0.036598626 | 1.438959328 | 0.525025815 |
| Phgdh | Q61753 | QDCEGLIVR_5994 | 0.004979295 | 1.411870436 | 0.497607702 |
| Srsf7 | Q8BL97 | FDRPPAR_5073 | 0.015527663 | 1.399926566 | 0.485351151 |
| Actr3 | Q99JY9 | GVDDLDFFIGDEAIEKPTYATK_4226 | 0.046728814 | 1.389236793 | 0.474292526 |
| Rpl3 | P27659 | KYCQVIR_11440 | 0.010103402 | 1.380886546 | 0.465594792 |
| Grb2 | Q60631 | HDGAFLIR_7799 | 0.032114609 | 1.376161098 | 0.460649366 |
| **Table S6. (Continued) Differential peptide profiles identified via Lip-MS spectrometry.** | | | | | |
| **Gene Name** | **Accession** | **Sequence** | **P-VALUE** | **Fold Change** | **Log_Fold Change** |
| Ipo11 | Q8K2V6 | VTEDEEPPTEQDKR_8182 | 0.018120643 | 1.364564217 | 0.44844029 |
| Ide | Q9JHR7 | EVNAVDSEHEK_3584 | 0.02841135 | 1.350642256 | 0.433645599 |
| Atg7 | Q9D906 | HVTFVDNAK_11163 | 0.012421125 | 1.345123793 | 0.427738952 |
| Hsp90aa1 | P07901 | LGIHEDSQNRK_9036 | 0.012324058 | 1.266760535 | 0.341143827 |
| Myo1e | E9Q634 | AEQEEYVQEGIR_4284 | 0.038636271 | 1.258929551 | 0.332197553 |
| Rpl34 | Q9D1R9 | SACGVCPGR_11420 | 0.047119766 | 1.252702166 | 0.32504345 |
| Rpl10 | Q6ZWV3 | EHVIEALR_7271 | 0.042310942 | 1.249692371 | 0.321573 |
| Xpnpep1 | Q6P1B1 | SLDEVYLIDSGAQYK_11248 | 0.042780214 | 1.246290442 | 0.31764032 |
| Slc6a6 | O35316 | DILKPSPGK_13114 | 0.049378081 | 1.243608547 | 0.314532437 |
| Sar1b | Q9CQC9 | LVDCADHER_18724 | 0.006053631 | 1.243512564 | 0.314421085 |
| Tufm | Q8BFR5 | YEEIDNAPEER_10500 | 0.03496561 | 1.231695862 | 0.300646061 |
| Eno2 | P17183 | CNCLLLK_1837 | 0.024355977 | 1.228265242 | 0.296622142 |
| Actn1 | Q7TPR4 | LDHLAEK_6071 | 0.015738545 | 1.225243385 | 0.293068357 |
| Cog2 | Q921L5 | LQEWLPDLLETVK_19659 | 0.01512093 | 1.223165775 | 0.290619945 |
| Gmps | Q3THK7 | DFMTGVPATPGNEIPVEVVLK_12614 | 0.000781027 | 1.213027834 | 0.278612655 |

| **Table S7. Results of high-throughput virtual screening.** | | | | | | |
| --- | --- | --- | --- | --- | --- | --- |
| **NO.** | **Gene name** | **Maximum similarity** | **Activity (nм)** | **overall** | **Number of similar ligands** | **Organism** |
| 1 | PDK4 | 0.5512 | 760 | 3.37288 | 1 | Homo sapiens (Human) |
| 2 | NR1I2 | 0.55406 | 434 | 3.52521 | 1 | Homo sapiens (Human) |
| 3 | NR1H3 | 0.55406 | 2920 | 3.06651 | 1 | Homo sapiens (Human) |
| 4 | NR1H2 | 0.55406 | 7500 | 2.83952 | 1 | Homo sapiens (Human) |
| 5 | FGFR4 | 0.56017 | 0.7 | 5.12826 | 1 | Homo sapiens (Human) |
| 6 | MEP1B | 0.5758 | 57 | 4.17117 | 1 | Homo sapiens (Human) |
| 7 | ECE1 | 0.57731 | 1700 | 3.3308 | 2 | Homo sapiens (Human) |
| 8 | CASP3 | 0.57781 | 30.97 | 4.33878 | 1 | Homo sapiens (Human) |
| 9 | KLKB1 | 0.57816 | 7600 | 2.95971 | 1 | Homo sapiens (Human) |
| 10 | DPP7 | 0.58022 | 3500 | 3.16562 | 1 | Homo sapiens (Human) |
| 11 | CCR2 | 0.58052 | 6.1 | 4.76876 | 1 | Homo sapiens (Human) |
| 12 | PTGER4 | 0.58168 | 4400 | 3.1158 | 1 | Homo sapiens (Human) |
| 13 | PTPN2 | 0.58184 | 550.3 | 3.64196 | 1 | Homo sapiens (Human) |
| 14 | PTPRF | 0.58184 | 624.7 | 3.60992 | 1 | Homo sapiens (Human) |
| 15 | ROCK1 | 0.58292 | 60 | 4.20973 | 2 | Homo sapiens (Human) |
| 16 | CCR1 | 0.58292 | 270 | 3.829 | 1 | Homo sapiens (Human) |
| 17 | PARP2 | 0.58295 | 1 | 5.24651 | 1 | Homo sapiens (Human) |
| 18 | TNKS | 0.58295 | 50 | 4.25611 | 1 | Homo sapiens (Human) |
| 19 | TNKS2 | 0.58295 | 120 | 4.03446 | 1 | Homo sapiens (Human) |
| 20 | GRM2 | 0.58454 | 1823 | 3.35482 | 3 | Homo sapiens (Human) |
| 21 | CCNB1 | 0.58483 | 8100 | 2.97767 | 1 | Homo sapiens (Human) |
| 22 | PPARD | 0.58561 | 17.1 | 4.5484 | 1 | Homo sapiens (Human) |
| 23 | PPARA | 0.58561 | 1000 | 3.51364 | 1 | Homo sapiens (Human) |
| 24 | KCNN3 | 0.58581 | 1000 | 3.51485 | 1 | Homo sapiens (Human) |
| **Table S7. (Continued) Results of high-throughput virtual screening.** | | | | | | |
| **NO.** | **Gene name** | **Maximum similarity** | **Activity (nм)** | **overall** | **Number of similar ligands** | **Organism** |
| 25 | CHEK1 | 0.58591 | 199.53 | 3.9256 | 2 | Homo sapiens (Human) |
| 26 | SLC6A4 | 0.58635 | 21 | 4.50191 | 4 | Homo sapiens (Human) |
| 27 | SLC6A2 | 0.58635 | 300 | 3.82472 | 2 | Homo sapiens (Human) |
| 28 | PPID | 0.58636 | 230 | 3.89243 | 1 | Homo sapiens (Human) |
| 29 | ST14 | 0.58682 | 191 | 3.94282 | 1 | Homo sapiens (Human) |
| 30 | NOS3 | 0.58695 | 8400 | 2.97918 | 3 | Homo sapiens (Human) |
| 31 | KIF11 | 0.58777 | 6900 | 3.03359 | 3 | Homo sapiens (Human) |
| 32 | CSNK1G1 | 0.5878 | 520 | 3.69371 | 2 | Homo sapiens (Human) |
| 33 | RPS6KA1 | 0.5878 | 780 | 3.59021 | 2 | Homo sapiens (Human) |
| 34 | AURKB | 0.5878 | 6000 | 3.06939 | 2 | Homo sapiens (Human) |
| 35 | AOC1 | 0.58789 | 5500 | 3.09209 | 1 | Homo sapiens (Human) |
| 36 | ABL1 | 0.58793 | 6600 | 3.04575 | 1 | Homo sapiens (Human) |
| 37 | CDK1 | 0.58793 | 7400 | 3.01654 | 2 | Homo sapiens (Human) |
| 38 | MME | 0.58826 | 140 | 4.03185 | 1 | Homo sapiens (Human) |
| 39 | DUSP22 | 0.5888 | 6600 | 3.05026 | 3 | Homo sapiens (Human) |
| 40 | NLRP3 | 0.58884 | 1540 | 3.4226 | 1 | Homo sapiens (Human) |
| 41 | HSD17B1 | 0.58884 | 2830 | 3.26699 | 1 | Homo sapiens (Human) |
| 42 | NMT1 | 0.58904 | 29.2 | 4.43818 | 1 | Homo sapiens (Human) |
| 43 | ODC1 | 0.5892 | 3600 | 3.20743 | 1 | Homo sapiens (Human) |
| 44 | BRD4 | 0.58939 | 6600 | 3.05332 | 2 | Homo sapiens (Human) |
| 45 | PTPRZ1 | 0.58954 | 5000 | 3.12519 | 1 | Homo sapiens (Human) |
| 46 | HTR5A | 0.58966 | 1920 | 3.37093 | 1 | Homo sapiens (Human) |
| 47 | CPA1 | 0.58984 | 1200 | 3.49237 | 1 | Homo sapiens (Human) |
| 48 | HDAC4 | 0.59066 | 170 | 3.99848 | 3 | Homo sapiens (Human) |
| **Table S7. (Continued) Results of high-throughput virtual screening.** | | | | | | |
| **NO.** | **Gene name** | **Maximum similarity** | **Activity (nм)** | **overall** | **Number of similar ligands** | **Organism** |
| 49 | ANO1 | 0.59068 | 890 | 3.57395 | 1 | Homo sapiens (Human) |
| 50 | TACR3 | 0.59083 | 9400 | 2.97001 | 2 | Homo sapiens (Human) |
| 51 | PPIA | 0.59158 | 370 | 3.80493 | 2 | Homo sapiens (Human) |
| 52 | KCNQ2 | 0.59167 | 690 | 3.64534 | 2 | Homo sapiens (Human) |
| 53 | TRPV4 | 0.59192 | 53 | 4.30663 | 1 | Homo sapiens (Human) |
| 54 | KAT2B | 0.592 | 30 | 4.45354 | 1 | Homo sapiens (Human) |
| 55 | ADAM17 | 0.5926 | 14.2 | 4.65052 | 1 | Homo sapiens (Human) |
| 56 | ADAM9 | 0.5926 | 700 | 3.64737 | 1 | Homo sapiens (Human) |
| 57 | SLC6A3 | 0.59294 | 1520 | 3.4498 | 3 | Homo sapiens (Human) |
| 58 | PDE7A | 0.59326 | 1700 | 3.42282 | 2 | Homo sapiens (Human) |
| 59 | PDE2A | 0.59394 | 7270 | 3.05195 | 1 | Homo sapiens (Human) |
| 60 | CNR1 | 0.59411 | 5700 | 3.11561 | 1 | Homo sapiens (Human) |
| 61 | KDM4C | 0.59433 | 2000 | 3.38706 | 2 | Homo sapiens (Human) |
| 62 | MALT1 | 0.59442 | 3390 | 3.25134 | 2 | Homo sapiens (Human) |
| 63 | NCOR1 | 0.59478 | 9520 | 2.98662 | 1 | Homo sapiens (Human) |
| 64 | PLA2G4B | 0.59488 | 4300 | 3.19246 | 1 | Homo sapiens (Human) |
| 65 | TNFRSF1A | 0.59549 | 8000 | 3.03516 | 1 | Homo sapiens (Human) |
| 66 | SSTR5 | 0.59558 | 1.1 | 5.33556 | 1 | Homo sapiens (Human) |
| 67 | ABCB1 | 0.59565 | 3790 | 3.22921 | 1 | Homo sapiens (Human) |
| 68 | CTSK | 0.59578 | 3 | 5.07778 | 1 | Homo sapiens (Human) |
| 69 | STS | 0.59601 | 220 | 3.96797 | 5 | Homo sapiens (Human) |
| 70 | IGF1R | 0.59646 | 61 | 4.30325 | 1 | Homo sapiens (Human) |
| 71 | ACSS2 | 0.59659 | 2.66 | 5.1158 | 1 | Homo sapiens (Human) |
| 72 | PKM | 0.5968 | 220 | 3.97327 | 1 | Homo sapiens (Human) |
| **Table S7. (Continued) Results of high-throughput virtual screening.** | | | | | | |
| **NO.** | **Gene name** | **Maximum similarity** | **Activity (nм)** | **overall** | **Number of similar ligands** | **Organism** |
| 73 | CHRM5 | 0.59695 | 42.5 | 4.40047 | 2 | Homo sapiens (Human) |
| 74 | CHRM4 | 0.59695 | 146.3 | 4.07999 | 1 | Homo sapiens (Human) |
| 75 | NOX4 | 0.5971 | 3700 | 3.2433 | 1 | Homo sapiens (Human) |
| 76 | BMP1 | 0.59725 | 80 | 4.23864 | 2 | Homo sapiens (Human) |
| 77 | DCTPP1 | 0.59805 | 990 | 3.59093 | 1 | Homo sapiens (Human) |
| 78 | DPP8 | 0.59869 | 555 | 3.74523 | 3 | Homo sapiens (Human) |
| 79 | IKBKB | 0.59893 | 6600 | 3.10275 | 1 | Homo sapiens (Human) |
| 80 | NPY5R | 0.59919 | 2900 | 3.31806 | 3 | Homo sapiens (Human) |
| 81 | HRH2 | 0.59973 | 1000 | 3.5984 | 3 | Homo sapiens (Human) |
| 82 | PLA2G7 | 0.60015 | 3800 | 3.25296 | 2 | Homo sapiens (Human) |
| 83 | ALOX15 | 0.6002 | 85 | 4.24376 | 3 | Homo sapiens (Human) |
| 84 | ALOX12 | 0.6002 | 2200 | 3.39567 | 2 | Homo sapiens (Human) |
| 85 | NOS1 | 0.60033 | 3300 | 3.29068 | 6 | Homo sapiens (Human) |
| 86 | GRIK1 | 0.60148 | 3700 | 3.26711 | 3 | Homo sapiens (Human) |
| 87 | DPP4 | 0.60248 | 610 | 3.7442 | 10 | Homo sapiens (Human) |
| 88 | CA14 | 0.60341 | 53.7 | 4.38684 | 1 | Homo sapiens (Human) |
| 89 | FKBP1A | 0.60366 | 47 | 4.42352 | 3 | Homo sapiens (Human) |
| 90 | AHR | 0.60397 | 7600 | 3.09186 | 2 | Homo sapiens (Human) |
| 91 | TYK2 | 0.60426 | 7 | 4.9277 | 2 | Homo sapiens (Human) |
| 92 | PDE4D | 0.60426 | 62 | 4.35528 | 3 | Homo sapiens (Human) |
| 93 | GCGR | 0.60481 | 4800 | 3.21683 | 2 | Homo sapiens (Human) |
| 94 | CXCR4 | 0.60529 | 91.2 | 4.26124 | 2 | Homo sapiens (Human) |
| 95 | CDC25C | 0.60529 | 3900 | 3.27399 | 1 | Homo sapiens (Human) |
| 96 | PRDX1 | 0.60566 | 950 | 3.64745 | 1 | Homo sapiens (Human) |
| **Table S7. (Continued) Results of high-throughput virtual screening.** | | | | | | |
| **NO.** | **Gene name** | **Maximum similarity** | **Activity (nм)** | **overall** | **Number of similar ligands** | **Organism** |
| 97 | SPR | 0.60588 | 550 | 3.7926 | 4 | Homo sapiens (Human) |
| 98 | CCR6 | 0.60603 | 1900 | 3.46728 | 2 | Homo sapiens (Human) |
| 99 | PREP | 0.60608 | 7 | 4.9425 | 3 | Homo sapiens (Human) |
| 100 | LSS | 0.60622 | 40 | 4.48477 | 1 | Homo sapiens (Human) |
| 101 | GRM7 | 0.60652 | 1584.89 | 3.51783 | 3 | Homo sapiens (Human) |
| 102 | SPIN1 | 0.60707 | 600 | 3.77707 | 1 | Homo sapiens (Human) |
| 103 | ESR2 | 0.60848 | 0.14 | 5.99588 | 2 | Homo sapiens (Human) |
| 104 | ESR1 | 0.60848 | 5.2 | 5.04065 | 2 | Homo sapiens (Human) |
| 105 | PLAT | 0.60851 | 35 | 4.53701 | 1 | Homo sapiens (Human) |
| 106 | MMP13 | 0.60868 | 3600 | 3.31348 | 2 | Homo sapiens (Human) |
| 107 | NOTUM | 0.60883 | 7900 | 3.10648 | 1 | Homo sapiens (Human) |
| 108 | CTH | 0.60898 | 2000 | 3.47053 | 1 | Homo sapiens (Human) |
| 109 | XDH | 0.60915 | 223 | 4.05189 | 3 | Homo sapiens (Human) |
| 110 | CXCR3 | 0.60924 | 5011.87 | 3.22898 | 2 | Homo sapiens (Human) |
| 111 | MMP8 | 0.60977 | 20 | 4.69457 | 16 | Homo sapiens (Human) |
| 112 | CBX7 | 0.60978 | 6100 | 3.17981 | 3 | Homo sapiens (Human) |
| 113 | CCR5 | 0.60982 | 980 | 3.66428 | 3 | Homo sapiens (Human) |
| 114 | PNP | 0.60997 | 6 | 5.01508 | 1 | Homo sapiens (Human) |
| 115 | CES1 | 0.61024 | 2000 | 3.47776 | 2 | Homo sapiens (Human) |
| 116 | KCNA3 | 0.6104 | 1400 | 3.57319 | 2 | Homo sapiens (Human) |
| 117 | KCNA4 | 0.6104 | 1500 | 3.5549 | 1 | Homo sapiens (Human) |
| 118 | KCNA6 | 0.6104 | 1600 | 3.53779 | 1 | Homo sapiens (Human) |
| 119 | KCNA5 | 0.6104 | 6100 | 3.18302 | 1 | Homo sapiens (Human) |
| 120 | PYGM | 0.61047 | 2100 | 3.46613 | 7 | Homo sapiens (Human) |
| **Table S7. (Continued) Results of high-throughput virtual screening.** | | | | | | |
| **NO.** | **Gene name** | **Maximum similarity** | **Activity (nм)** | **overall** | **Number of similar ligands** | **Organism** |
| 121 | BACE1 | 0.61048 | 333 | 3.95444 | 2 | Homo sapiens (Human) |
| 122 | MAPK10 | 0.61056 | 8890 | 3.08401 | 4 | Homo sapiens (Human) |
| 123 | ADRA2C | 0.61065 | 19.6 | 4.70672 | 3 | Homo sapiens (Human) |
| 124 | ADRA2B | 0.61065 | 72.8 | 4.35873 | 4 | Homo sapiens (Human) |
| 125 | ADRA2A | 0.61065 | 146.1 | 4.17399 | 5 | Homo sapiens (Human) |
| 126 | CFTR | 0.61066 | 9000 | 3.08126 | 5 | Homo sapiens (Human) |
| 127 | PYGL | 0.61074 | 6400 | 3.17206 | 6 | Homo sapiens (Human) |
| 128 | CTRB1 | 0.61146 | 3800 | 3.31425 | 4 | Homo sapiens (Human) |
| 129 | QPCT | 0.61233 | 7.3 | 4.98233 | 5 | Homo sapiens (Human) |
| 130 | CHRM3 | 0.61237 | 25.12 | 4.65398 | 4 | Homo sapiens (Human) |
| 131 | CHRM2 | 0.61237 | 100 | 4.28657 | 3 | Homo sapiens (Human) |
| 132 | CHRM1 | 0.61237 | 630.96 | 3.79667 | 3 | Homo sapiens (Human) |
| 133 | CDK4 | 0.61308 | 1200 | 3.62991 | 13 | Homo sapiens (Human) |
| 134 | PTGS1 | 0.61487 | 240 | 4.07033 | 6 | Homo sapiens (Human) |
| 135 | ADORA2B | 0.61516 | 46.77 | 4.50911 | 3 | Homo sapiens (Human) |
| 136 | CTSL | 0.61543 | 560 | 3.84758 | 5 | Homo sapiens (Human) |
| 137 | CTSS | 0.61543 | 1600 | 3.56699 | 2 | Homo sapiens (Human) |
| 138 | CTSB | 0.61543 | 3000 | 3.39897 | 4 | Homo sapiens (Human) |
| 139 | PROKR1 | 0.61547 | 3690 | 3.34383 | 3 | Homo sapiens (Human) |
| 140 | PNMT | 0.61565 | 7000 | 3.17359 | 1 | Homo sapiens (Human) |
| 141 | PTGS2 | 0.61635 | 175 | 4.16466 | 8 | Homo sapiens (Human) |
| 142 | LIPE | 0.61659 | 205 | 4.12389 | 6 | Homo sapiens (Human) |
| 143 | CCKBR | 0.61723 | 3100 | 3.40012 | 3 | Homo sapiens (Human) |
| 144 | HPN | 0.61743 | 272 | 4.05372 | 2 | Homo sapiens (Human) |
| **Table S7. (Continued) Results of high-throughput virtual screening.** | | | | | | |
| **NO.** | **Gene name** | **Maximum similarity** | **Activity (nм)** | **overall** | **Number of similar ligands** | **Organism** |
| 145 | MAPK14 | 0.61757 | 967 | 3.7144 | 6 | Homo sapiens (Human) |
| 146 | PLCG2 | 0.61781 | 2000 | 3.52086 | 3 | Homo sapiens (Human) |
| 147 | PLAU | 0.61813 | 770 | 3.77894 | 6 | Homo sapiens (Human) |
| 148 | HDAC6 | 0.61816 | 37 | 4.59406 | 6 | Homo sapiens (Human) |
| 149 | HDAC3 | 0.61816 | 1300 | 3.63854 | 3 | Homo sapiens (Human) |
| 150 | MAPKAPK2 | 0.61837 | 2300 | 3.48655 | 7 | Homo sapiens (Human) |
| 151 | ITGB3 | 0.61863 | 37 | 4.59751 | 7 | Homo sapiens (Human) |
| 152 | CAPN1 | 0.61996 | 5000 | 3.2864 | 6 | Homo sapiens (Human) |
| 153 | CTRC | 0.61999 | 0.69 | 5.67979 | 1 | Homo sapiens (Human) |
| 154 | ELANE | 0.61999 | 100 | 4.3399 | 4 | Homo sapiens (Human) |
| 155 | PLG | 0.61999 | 2900 | 3.43324 | 1 | Homo sapiens (Human) |
| 156 | PRMT5 | 0.62022 | 109 | 4.31833 | 1 | Homo sapiens (Human) |
| 157 | GHSR | 0.62028 | 90 | 4.37031 | 2 | Homo sapiens (Human) |
| 158 | TRPM8 | 0.62052 | 0.7 | 5.68085 | 6 | Homo sapiens (Human) |
| 159 | C1R | 0.62123 | 5100 | 3.28784 | 18 | Homo sapiens (Human) |
| 160 | THRA | 0.62182 | 3000 | 3.43424 | 1 | Homo sapiens (Human) |
| 161 | THRB | 0.62182 | 3800 | 3.3704 | 1 | Homo sapiens (Human) |
| 162 | PTGER2 | 0.62186 | 2 | 5.40952 | 2 | Homo sapiens (Human) |
| 163 | PTPN1 | 0.62189 | 8000 | 3.1697 | 3 | Homo sapiens (Human) |
| 164 | DUSP3 | 0.62189 | 8500 | 3.15332 | 1 | Homo sapiens (Human) |
| 165 | CYP19A1 | 0.6227 | 4600 | 3.32351 | 10 | Homo sapiens (Human) |
| 166 | CHRNA7 | 0.62274 | 2000 | 3.54901 | 1 | Homo sapiens (Human) |
| 167 | GABRG2 | 0.62309 | 426.58 | 3.96906 | 8 | Homo sapiens (Human) |
| 168 | CASR | 0.6233 | 2300 | 3.51432 | 16 | Homo sapiens (Human) |
| **Table S7. (Continued) Results of high-throughput virtual screening.** | | | | | | |
| **NO.** | **Gene name** | **Maximum similarity** | **Activity (nм)** | **overall** | **Number of similar ligands** | **Organism** |
| 169 | MMP3 | 0.62371 | 700 | 3.83889 | 10 | Homo sapiens (Human) |
| 170 | TDP1 | 0.62392 | 1080 | 3.72264 | 1 | Homo sapiens (Human) |
| 171 | F7 | 0.62426 | 2170 | 3.53551 | 10 | Homo sapiens (Human) |
| 172 | MMP2 | 0.62432 | 27 | 4.72524 | 7 | Homo sapiens (Human) |
| 173 | MMP15 | 0.62432 | 29 | 4.70586 | 4 | Homo sapiens (Human) |
| 174 | MMP16 | 0.62432 | 40 | 4.61867 | 4 | Homo sapiens (Human) |
| 175 | MMP12 | 0.62432 | 65 | 4.48703 | 4 | Homo sapiens (Human) |
| 176 | MMP26 | 0.62432 | 146 | 4.26762 | 4 | Homo sapiens (Human) |
| 177 | MMP14 | 0.62432 | 867 | 3.7846 | 4 | Homo sapiens (Human) |
| 178 | PHGDH | 0.62432 | 6800 | 3.22617 | 6 | Homo sapiens (Human) |
| 179 | ALDH2 | 0.62504 | 7000 | 3.22201 | 1 | Homo sapiens (Human) |
| 180 | F2RL1 | 0.62525 | 6309.57 | 3.25129 | 4 | Homo sapiens (Human) |
| 181 | OPRL1 | 0.62526 | 6 | 5.14076 | 14 | Homo sapiens (Human) |
| 182 | IDO1 | 0.62529 | 2310 | 3.52437 | 5 | Homo sapiens (Human) |
| 183 | CA7 | 0.62572 | 1.9 | 5.45706 | 6 | Homo sapiens (Human) |
| 184 | CA4 | 0.62572 | 377.7 | 4.01891 | 9 | Homo sapiens (Human) |
| 185 | FGFR1 | 0.62713 | 600 | 3.90192 | 3 | Homo sapiens (Human) |
| 186 | MET | 0.62713 | 760 | 3.83754 | 3 | Homo sapiens (Human) |
| 187 | KIT | 0.62713 | 5900 | 3.27937 | 3 | Homo sapiens (Human) |
| 188 | KDR | 0.62713 | 8600 | 3.17674 | 5 | Homo sapiens (Human) |
| 189 | CCR3 | 0.6283 | 8300 | 3.19235 | 4 | Homo sapiens (Human) |
| 190 | SLC18A3 | 0.62835 | 30 | 4.72699 | 14 | Homo sapiens (Human) |
| 191 | P2RX7 | 0.62839 | 63.1 | 4.52439 | 1 | Homo sapiens (Human) |
| 192 | HTR1A | 0.62978 | 38 | 4.67313 | 51 | Homo sapiens (Human) |
| **Table S7. (Continued) Results of high-throughput virtual screening.** | | | | | | |
| **NO.** | **Gene name** | **Maximum similarity** | **Activity (nм)** | **overall** | **Number of similar ligands** | **Organism** |
| 193 | HTR2B | 0.62978 | 140 | 4.31646 | 8 | Homo sapiens (Human) |
| 194 | HTR1D | 0.62978 | 140 | 4.31646 | 4 | Homo sapiens (Human) |
| 195 | CYP3A43 | 0.62978 | 1900 | 3.60315 | 1 | Homo sapiens (Human) |
| 196 | HTR1F | 0.62978 | 7200 | 3.23877 | 1 | Homo sapiens (Human) |
| 197 | CTSG | 0.63014 | 4500 | 3.3692 | 26 | Homo sapiens (Human) |
| 198 | PSMB5 | 0.63028 | 2010 | 3.5906 | 2 | Homo sapiens (Human) |
| 199 | PSMB1 | 0.63028 | 2640 | 3.51597 | 2 | Homo sapiens (Human) |
| 200 | KAT6A | 0.63031 | 390 | 4.0396 | 6 | Homo sapiens (Human) |
| 201 | HDAC1 | 0.63111 | 4200 | 3.39332 | 8 | Homo sapiens (Human) |
| 202 | HDAC2 | 0.63111 | 8100 | 3.21331 | 2 | Homo sapiens (Human) |
| 203 | BCHE | 0.63153 | 5000 | 3.34778 | 5 | Homo sapiens (Human) |
| 204 | MAP2K1 | 0.63164 | 3200 | 3.47076 | 3 | Homo sapiens (Human) |
| 205 | PARP1 | 0.63177 | 389.05 | 4.04967 | 6 | Homo sapiens (Human) |
| 206 | CTSC | 0.63279 | 790 | 3.86154 | 2 | Homo sapiens (Human) |
| 207 | RASGRP3 | 0.63321 | 23.8 | 4.8272 | 9 | Homo sapiens (Human) |
| 208 | PRKCE | 0.63321 | 328 | 4.10579 | 6 | Homo sapiens (Human) |
| 209 | PRKCA | 0.63321 | 1020 | 3.79379 | 9 | Homo sapiens (Human) |
| 210 | TBXA2R | 0.63325 | 7760 | 3.236 | 6 | Homo sapiens (Human) |
| 211 | MTAP | 0.63395 | 0.27 | 6.06601 | 5 | Homo sapiens (Human) |
| 212 | HRH4 | 0.63396 | 25.12 | 4.81808 | 17 | Homo sapiens (Human) |
| 213 | METAP2 | 0.63419 | 420 | 4.04408 | 4 | Homo sapiens (Human) |
| 214 | INCENP | 0.63438 | 7400 | 3.25485 | 3 | Homo sapiens (Human) |
| 215 | F2 | 0.63454 | 3000 | 3.5045 | 36 | Homo sapiens (Human) |
| 216 | CMA1 | 0.63459 | 2700 | 3.53382 | 43 | Homo sapiens (Human) |
| **Table S7. (Continued) Results of high-throughput virtual screening.** | | | | | | |
| **NO.** | **Gene name** | **Maximum similarity** | **Activity (nм)** | **overall** | **Number of similar ligands** | **Organism** |
| 217 | EHMT2 | 0.63567 | 450 | 4.03444 | 1 | Homo sapiens (Human) |
| 218 | EHMT1 | 0.63567 | 1000 | 3.814 | 1 | Homo sapiens (Human) |
| 219 | TRPV1 | 0.6363 | 4000 | 3.4347 | 38 | Homo sapiens (Human) |
| 220 | RAPGEF4 | 0.63684 | 3400 | 3.48257 | 3 | Homo sapiens (Human) |
| 221 | RAPGEF3 | 0.63684 | 6400 | 3.30763 | 4 | Homo sapiens (Human) |
| 222 | HRH3 | 0.6376 | 87 | 4.50176 | 19 | Homo sapiens (Human) |
| 223 | MMP1 | 0.63768 | 215 | 4.25178 | 12 | Homo sapiens (Human) |
| 224 | CDK2 | 0.63815 | 6100 | 3.32773 | 9 | Homo sapiens (Human) |
| 225 | ACHE | 0.63873 | 6000 | 3.33537 | 21 | Homo sapiens (Human) |
| 226 | RIPK1 | 0.6391 | 10 | 5.11279 | 2 | Homo sapiens (Human) |
| 227 | ADRA1A | 0.64054 | 3.981 | 5.38055 | 9 | Homo sapiens (Human) |
| 228 | ADRA1D | 0.64054 | 158.49 | 4.35568 | 7 | Homo sapiens (Human) |
| 229 | ESRRB | 0.64081 | 2700 | 3.56842 | 3 | Homo sapiens (Human) |
| 230 | MGAM2 | 0.6409 | 1100 | 3.8189 | 21 | Homo sapiens (Human) |
| 231 | MAPKAP1 | 0.64127 | 1 | 5.77142 | 1 | Homo sapiens (Human) |
| 232 | MLST8 | 0.64127 | 1 | 5.77142 | 1 | Homo sapiens (Human) |
| 233 | MCHR1 | 0.64128 | 1 | 5.77156 | 7 | Homo sapiens (Human) |
| 234 | PTAFR | 0.64223 | 300 | 4.18916 | 6 | Homo sapiens (Human) |
| 235 | CRHR1 | 0.64378 | 1403 | 3.768 | 22 | Homo sapiens (Human) |
| 236 | TNF | 0.64443 | 2130 | 3.65498 | 8 | Homo sapiens (Human) |
| 237 | SCN5A | 0.64454 | 5250 | 3.40305 | 19 | Homo sapiens (Human) |
| 238 | JAK1 | 0.64497 | 3380 | 3.52866 | 2 | Homo sapiens (Human) |
| 239 | PSMB2 | 0.645 | 6710 | 3.33677 | 2 | Homo sapiens (Human) |
| 240 | HSD11B1 | 0.64524 | 1090 | 3.84729 | 19 | Homo sapiens (Human) |
| **Table S7. (Continued) Results of high-throughput virtual screening.** | | | | | | |
| **NO.** | **Gene name** | **Maximum similarity** | **Activity (nм)** | **overall** | **Number of similar ligands** | **Organism** |
| 241 | SLC6A9 | 0.6456 | 530 | 4.05159 | 4 | Homo sapiens (Human) |
| 242 | SLC6A5 | 0.6456 | 8300 | 3.28023 | 1 | Homo sapiens (Human) |
| 243 | ALOX5 | 0.64618 | 767.4 | 3.95138 | 2 | Homo sapiens (Human) |
| 244 | UTS2R | 0.64704 | 62 | 4.66363 | 14 | Homo sapiens (Human) |
| 245 | MMP9 | 0.64744 | 8500 | 3.28289 | 11 | Homo sapiens (Human) |
| 246 | PGR | 0.64751 | 521 | 4.0684 | 19 | Homo sapiens (Human) |
| 247 | ESRRG | 0.64766 | 4400 | 3.46924 | 10 | Homo sapiens (Human) |
| 248 | SCN2A | 0.64906 | 3870 | 3.5129 | 23 | Homo sapiens (Human) |
| 249 | TMEM97 | 0.64962 | 350 | 4.19388 | 12 | Homo sapiens (Human) |
| 250 | ADRA1B | 0.64985 | 1.16 | 5.80676 | 23 | Homo sapiens (Human) |
| 251 | HRH1 | 0.65002 | 718 | 3.99367 | 7 | Homo sapiens (Human) |
| 252 | TACR1 | 0.65115 | 1530 | 3.78666 | 37 | Homo sapiens (Human) |
| 253 | OPRD1 | 0.65121 | 846 | 3.95453 | 35 | Homo sapiens (Human) |
| 254 | KCNH2 | 0.65121 | 1840 | 3.73479 | 25 | Homo sapiens (Human) |
| 255 | GSK3B | 0.65162 | 2350 | 3.66792 | 13 | Homo sapiens (Human) |
| 256 | HTR2A | 0.65295 | 0.51 | 6.06746 | 45 | Homo sapiens (Human) |
| 257 | HTR7 | 0.65295 | 8.76 | 5.26112 | 16 | Homo sapiens (Human) |
| 258 | HTR2C | 0.65295 | 16.6 | 5.07986 | 43 | Homo sapiens (Human) |
| 259 | HTR6 | 0.65295 | 48.9 | 4.77349 | 8 | Homo sapiens (Human) |
| 260 | ADORA2A | 0.65297 | 2.5 | 5.61684 | 19 | Homo sapiens (Human) |
| 261 | ADORA1 | 0.65297 | 21.3 | 5.0093 | 12 | Homo sapiens (Human) |
| 262 | ADORA3 | 0.65297 | 100 | 4.57076 | 12 | Homo sapiens (Human) |
| 263 | SRC | 0.65346 | 3500 | 3.56524 | 5 | Homo sapiens (Human) |
| 264 | F10 | 0.65387 | 270 | 4.29505 | 40 | Homo sapiens (Human) |
| **Table S7. (Continued) Results of high-throughput virtual screening.** | | | | | | |
| **NO.** | **Gene name** | **Maximum similarity** | **Activity (nм)** | **overall** | **Number of similar ligands** | **Organism** |
| 265 | PRSS1 | 0.65387 | 700 | 4.02452 | 11 | Homo sapiens (Human) |
| 266 | DHFR | 0.65392 | 44 | 4.81062 | 4 | Homo sapiens (Human) |
| 267 | CDY1 | 0.65499 | 1000 | 3.92996 | 3 | Homo sapiens (Human) |
| 268 | CDYL2 | 0.65499 | 7200 | 3.36841 | 4 | Homo sapiens (Human) |
| 269 | PPARG | 0.65689 | 2098 | 3.72994 | 7 | Homo sapiens (Human) |
| 270 | GRIN3B | 0.65833 | 7 | 5.36859 | 13 | Homo sapiens (Human) |
| 271 | SIGMAR1 | 0.65856 | 0.17 | 6.43382 | 134 | Homo sapiens (Human) |
| 272 | CDC25B | 0.66063 | 2800 | 3.66836 | 11 | Homo sapiens (Human) |
| 273 | CDC25A | 0.66063 | 7800 | 3.37442 | 3 | Homo sapiens (Human) |
| 274 | GRIN2B | 0.66072 | 1 | 5.94649 | 34 | Homo sapiens (Human) |
| 275 | AR | 0.66298 | 5950 | 3.46438 | 8 | Homo sapiens (Human) |
| 276 | CDYL | 0.66394 | 1200 | 3.93106 | 4 | Homo sapiens (Human) |
| 277 | MAOB | 0.66478 | 510 | 4.18305 | 26 | Homo sapiens (Human) |
| 278 | EPHX2 | 0.66495 | 700 | 4.09272 | 5 | Homo sapiens (Human) |
| 279 | CYP1B1 | 0.66586 | 5200 | 3.51839 | 13 | Homo sapiens (Human) |
| 280 | CYP1A1 | 0.66586 | 6200 | 3.46753 | 11 | Homo sapiens (Human) |
| 281 | MYOC | 0.66698 | 99 | 4.6718 | 1 | Homo sapiens (Human) |
| 282 | PDE4B | 0.66762 | 70.79 | 4.77348 | 8 | Homo sapiens (Human) |
| 283 | ADRB3 | 0.67011 | 30 | 5.04115 | 28 | Homo sapiens (Human) |
| 284 | ADRB1 | 0.67011 | 900 | 4.05132 | 26 | Homo sapiens (Human) |
| 285 | CYP3A4 | 0.67084 | 5100 | 3.55039 | 16 | Homo sapiens (Human) |
| 286 | CA12 | 0.67225 | 7.6 | 5.45811 | 16 | Homo sapiens (Human) |
| 287 | CDC7 | 0.67456 | 30 | 5.07465 | 15 | Homo sapiens (Human) |
| 288 | HCRTR2 | 0.67563 | 2100 | 3.83607 | 12 | Homo sapiens (Human) |
| **Table S7. (Continued) Results of high-throughput virtual screening.** | | | | | | |
| **NO.** | **Gene name** | **Maximum similarity** | **Activity (nм)** | **overall** | **Number of similar ligands** | **Organism** |
| 289 | YARS1 | 0.67784 | 900 | 4.09806 | 7 | Homo sapiens (Human) |
| 290 | MAOA | 0.67919 | 4160 | 3.65466 | 12 | Homo sapiens (Human) |
| 291 | OPRM1 | 0.68171 | 1290 | 4.01485 | 52 | Homo sapiens (Human) |
| 292 | DYRK1A | 0.6826 | 600 | 4.24702 | 26 | Homo sapiens (Human) |
| 293 | LCK | 0.68405 | 1700 | 3.94668 | 17 | Homo sapiens (Human) |
| 294 | CYP1A2 | 0.68414 | 3700 | 3.7161 | 16 | Homo sapiens (Human) |
| 295 | KDM1A | 0.68421 | 6200 | 3.56308 | 31 | Homo sapiens (Human) |
| 296 | HSD17B2 | 0.68496 | 1570 | 3.97559 | 6 | Homo sapiens (Human) |
| 297 | ADRB2 | 0.68656 | 180 | 4.63068 | 33 | Homo sapiens (Human) |
| 298 | AURKA | 0.6875 | 3300 | 3.76854 | 2 | Homo sapiens (Human) |
| 299 | TTK | 0.6875 | 5400 | 3.6215 | 4 | Homo sapiens (Human) |
| 300 | DRD4 | 0.68758 | 91.8 | 4.83859 | 128 | Homo sapiens (Human) |
| 301 | DRD2 | 0.68758 | 300 | 4.48499 | 169 | Homo sapiens (Human) |
| 302 | DRD3 | 0.68758 | 400 | 4.39908 | 101 | Homo sapiens (Human) |
| 303 | IDH1 | 0.69139 | 913 | 4.17566 | 15 | Homo sapiens (Human) |
| 304 | APP | 0.69377 | 8410 | 3.52101 | 6 | Homo sapiens (Human) |
| 305 | OPRK1 | 0.69472 | 6600 | 3.59899 | 30 | Homo sapiens (Human) |
| 306 | LY96 | 0.70444 | 4220 | 3.78614 | 2 | Homo sapiens (Human) |
| 307 | NQO1 | 0.70777 | 320 | 4.59685 | 5 | Homo sapiens (Human) |
| 308 | NOS2 | 0.71048 | 1010 | 4.2598 | 10 | Homo sapiens (Human) |
| 309 | MIF | 0.71306 | 1600 | 4.13283 | 6 | Homo sapiens (Human) |
| 310 | CXCL12 | 0.7222 | 203 | 4.83334 | 34 | Homo sapiens (Human) |
| 311 | CYP2C9 | 0.72777 | 1800 | 4.18085 | 25 | Homo sapiens (Human) |
| 312 | CYP2D6 | 0.72777 | 2000 | 4.14755 | 20 | Homo sapiens (Human) |
| **Table S7. (Continued) Results of high-throughput virtual screening.** | | | | | | |
| **NO.** | **Gene name** | **Maximum similarity** | **Activity (nм)** | **overall** | **Number of similar ligands** | **Organism** |
| 313 | ERBB2 | 0.72909 | 2884.03 | 4.03915 | 2 | Homo sapiens (Human) |
| 314 | EGFR | 0.72909 | 3019.95 | 4.02457 | 10 | Homo sapiens (Human) |
| 315 | Mchr1 | 0.5764 | 540 | 3.61266 | 1 | Mus musculus (Mouse) |
| 316 | Htr3b | 0.58552 | 3470 | 3.19677 | 1 | Mus musculus (Mouse) |
| 317 | Oprm1 | 0.58928 | 2.1 | 5.11365 | 1 | Mus musculus (Mouse) |
| 318 | Sstr5 | 0.59558 | 21 | 4.57272 | 1 | Mus musculus (Mouse) |
| 319 | Maoa | 0.6036 | 40 | 4.4654 | 1 | Mus musculus (Mouse) |
| 320 | Ctsg | 0.604 | 690 | 3.72134 | 1 | Mus musculus (Mouse) |
| 321 | Prkca | 0.60637 | 102.8 | 4.23728 | 5 | Mus musculus (Mouse) |
| 322 | Scd1 | 0.60775 | 590 | 3.78577 | 4 | Mus musculus (Mouse) |
| 323 | P2rx7 | 0.60972 | 3170 | 3.35281 | 1 | Mus musculus (Mouse) |
| 324 | Oprd1 | 0.61284 | 35.48 | 4.56567 | 5 | Mus musculus (Mouse) |
| 325 | Ptgs1 | 0.61435 | 9010 | 3.09959 | 1 | Mus musculus (Mouse) |
| 326 | Prtn3 | 0.61543 | 4550 | 3.28765 | 2 | Mus musculus (Mouse) |
| 327 | Kcnq2 | 0.62004 | 940 | 3.73688 | 2 | Mus musculus (Mouse) |
| 328 | Nlrp3 | 0.62077 | 5000 | 3.29073 | 1 | Mus musculus (Mouse) |
| 329 | Il5 | 0.62133 | 5000 | 3.29369 | 2 | Mus musculus (Mouse) |
| 330 | Ache | 0.62242 | 94 | 4.37363 | 11 | Mus musculus (Mouse) |
| 331 | Ptges | 0.6278 | 1700 | 3.6221 | 1 | Mus musculus (Mouse) |
| 332 | Tpsb2 | 0.63012 | 2310 | 3.55158 | 4 | Mus musculus (Mouse) |
| 333 | Elane | 0.63279 | 7930 | 3.2277 | 3 | Mus musculus (Mouse) |
| 334 | Ptgs2 | 0.63595 | 7920 | 3.24417 | 8 | Mus musculus (Mouse) |
| 335 | Alox5 | 0.63772 | 5900 | 3.33475 | 9 | Mus musculus (Mouse) |
| 336 | Htr1a | 0.65929 | 1000 | 3.95573 | 4 | Mus musculus (Mouse) |
